# Glutamine metabolism in health and disease

**DOI:** 10.1038/s41392-026-02981-1

**Published:** 2026-10-06

**Authors:** Junsheng Zhao, Yajie Lin, Rong Gao, Hainv Gao, Lanjuan Li

**Affiliations:** 1 Bioinformatic Center, Key Laboratory of Artificial Organs and Computational Medicine of Zhejiang Province, Shulan (Hangzhou) Hospital, Shulan International Medical College, Zhejiang Shuren University, Hangzhou, China; 2 State Key Laboratory for Diagnosis and Treatment of Infectious Diseases, National Clinical Research Center for Infectious Diseases, National Medical Center for Infectious Diseases, Collaborative Innovation Center for Diagnosis and Treatment of Infectious Diseases, The First Affiliated Hospital, Zhejiang University School of Medicine, Hangzhou, China; 3 Department of Infection, Key Laboratory of Artificial Organs and Computational Medicine in Zhejiang Province, Shulan (Hangzhou) Hospital Affiliated to Shulan International Medical College, Zhejiang Shuren University, Hangzhou, China

**Keywords:** Structural biology, Metabolic disorders

## Abstract

Glutamine is the most abundant free amino acid in the body. It plays a central role in cellular metabolism, connecting synthetic and catabolic pathways, and maintaining life activities under physiological and pathological conditions. This review systematically explores the dual role of glutamine metabolism in health and disease. Glutamine is not only the main energy and nitrogen source for rapidly proliferating cells, such as immune cells, intestinal epithelial cells, and cancer cells but also participates in several key biological processes, such as nucleotide synthesis, REDOX balance, and immune regulation. Glutamine metabolism is often abnormally activated or disrupted in disease states, such as “glutamine addiction” in cancer and excitotoxicity and mitochondrial dysfunction in neurodegenerative diseases. Disorders of glutamine metabolism are also present in metabolic diseases (such as diabetes and MAFLD) and cardiovascular diseases. In addition, gut microbes regulate host glutamine metabolism through metabolites and signaling pathways, further affecting the disease process. Therapeutic strategies to target glutamine metabolism include inhibition of key enzymes or transporters, as well as exogenous glutamine supplementation. However, the effects of these strategies are context-dependent and need to be applied individually according to the specific pathological status of patients. Future research should focus on tissue-specific metabolic profiling, integrated multiomics analysis, and the development of combined treatment strategies to fully realize the potential of glutamine metabolism in disease treatment.

## Introduction

Glutamine, traditionally classified as a nutritionally nonessential amino acid (NEAA), is the most abundant free amino acid and occupies a central position in cellular metabolism, bridging anabolic and catabolic pathways to sustain life under both physiological and pathological conditions.^1^ Beyond its role as a simple building block for proteins, glutamine fuels biosynthetic and bioenergetic processes, regulates redox balance, modulates gene expression and signaling, and acts as a key substrate for immune cell function.^2,3^ This metabolic versatility underpins its designation as a conditionally essential amino acid (EAA) during stress, growth, and specific pathological states, where its demand can outstrip endogenous synthesis.^2,4^

In physiological contexts, glutamine metabolism is tightly regulated and fundamental to interorgan metabolic communication. The gut-liver axis is a prime example, where glutamine serves as a primary respiratory fuel for enterocytes, a precursor for mucosal glutathione (GSH) synthesis crucial for barrier integrity, and a substrate for hepatic ureagenesis and gluconeogenesis.^1,2^ Skeletal muscle acts as a major reservoir, releasing glutamine into circulation, particularly during stress, to support the needs of immune cells and visceral organs.^5^ Furthermore, glutamine is integral to renal ammoniagenesis for acid‒base homeostasis and is a precursor for the synthesis of citrulline and arginine, highlighting its systemic role in nitrogen handling.^1^ This complex homeostasis is essential for normal growth, development, and the response to physiological challenges, including exercise and pregnancy.^2,6,7^ Glutamine can be used to produce UDP-GlcNAc with glucose, a key molecule for protein glycosylation. Glutamine availability modulates the hexosamine biosynthesis pathway (HBP) flux by serving as a nitrogen source for UDP-GlcNAc production and contributing to acetyl-CoA generation.^8–11^

However, the pathways that sustain life in health are often hijacked in disease. Cancer cells, for example, exhibit a voracious appetite for glutamine, a phenomenon termed “glutamine addiction”.^3,12,13^ Beyond oncology, dysregulated glutamine metabolism is implicated in a broad spectrum of diseases. In cardiovascular and metabolic disorders, glutamine influences endothelial function and nitric oxide synthesis and mitigates risk factors such as hypertension and hyperlipidemia, although aberrant glutaminolysis can contribute to pathologies such as pulmonary arterial hypertension.^14^ Within the nervous system, the glutamate-glutamine cycle between astrocytes and neurons is fundamental for neurotransmission, and its disruption is linked to neurodegenerative and neuropsychiatric conditions.^15,16^ Glutamine is positioned as a key nutrient in immunonutrition for critical illness, sepsis, and postsurgical recovery.^17^ Moreover, it is implicated in gastrointestinal diseases and catabolic stress conditions due to its essential role in maintaining intestinal barrier function.^2^

Hyperpolarized magnetic resonance spectroscopy (MRS) using 13C-labeled glutamine allows real-time, noninvasive assessment of metabolic fluxes in vivo, offering profound insights into tumor metabolism and neurological function.^15^ The development of positron emission tomography (PET) tracers such as 18F-(2S,4R)-4-fluoroglutamine (18F-FGln) enables the imaging of glutamine uptake in tumors, providing a potential tool for diagnosis, staging, and monitoring therapeutic response.^15^ Concurrently, the exploration of pharmacological agents to deplete or disrupt glutamine metabolism, either as monotherapies or in rational combination with chemotherapy, immunotherapy, or radiotherapy, represents an active frontier in translational research.^16,18,19^

This review aims to provide a comprehensive synthesis of the multifaceted roles of glutamine in human physiology and pathogenesis. We will delve into the fundamentals of its metabolism and transport, its reprogramming in cancer biology, its immunomodulatory functions, and its involvement in metabolic, cardiovascular, fibrotic, and neurological disorders. We will also critically evaluate the clinical applications of glutamine supplementation in nutritional support and critical care and explore the emerging diagnostic and therapeutic frontiers that target this pivotal metabolic pathway. By integrating insights from molecular studies, preclinical models, and clinical trials, this work underscores glutamine’s position as a critical metabolic nexus and a promising target for therapeutic intervention across a wide spectrum of human diseases.

## Fundamentals of glutamine metabolism and homeostasis

### Absorption, transport, and utilization of glutamine

Glutamine is predominantly obtained from dietary protein. In the gastrointestinal tract, proteolytic enzymes, including pepsin and trypsin, hydrolyze proteins into oligopeptides and free amino acids. Once inside enterocytes, glutamine serves as the primary energy source for enterocytes.^20^ Approximately 30% of dietary glutamine is utilized by the gut, while the remainder enters systemic circulation. Glutamine circulates in plasma at 500–800 μM, serving as a nitrogen carrier and being transported to peripheral tissues, where its uptake is mediated by transporters, which are upregulated in proliferating cells such as cancer and immune cells.^21^

Transamination reactions represent a critical metabolic nexus linking glutamine-derived glutamate to the synthesis of other amino acids and the generation of key metabolic intermediates. Through transaminases such as glutamate pyruvate transaminase (GPT) and glutamate oxaloacetate transaminase (GOT), the amino group from glutamate can be transferred to various ketoacids, producing alanine, aspartate, and other NEAAs while regenerating α-ketoglutarate (α-KG).^3,22^ This process is vital for maintaining amino acid homeostasis. The production of aspartate from glutamate is crucial for pyrimidine synthesis, directly linking glutaminolysis to the nucleotide demand of proliferating cells.^23^ The nitrogen from glutamine is essential for the de novo synthesis of nucleotides.^24^

**Fig. 1:**
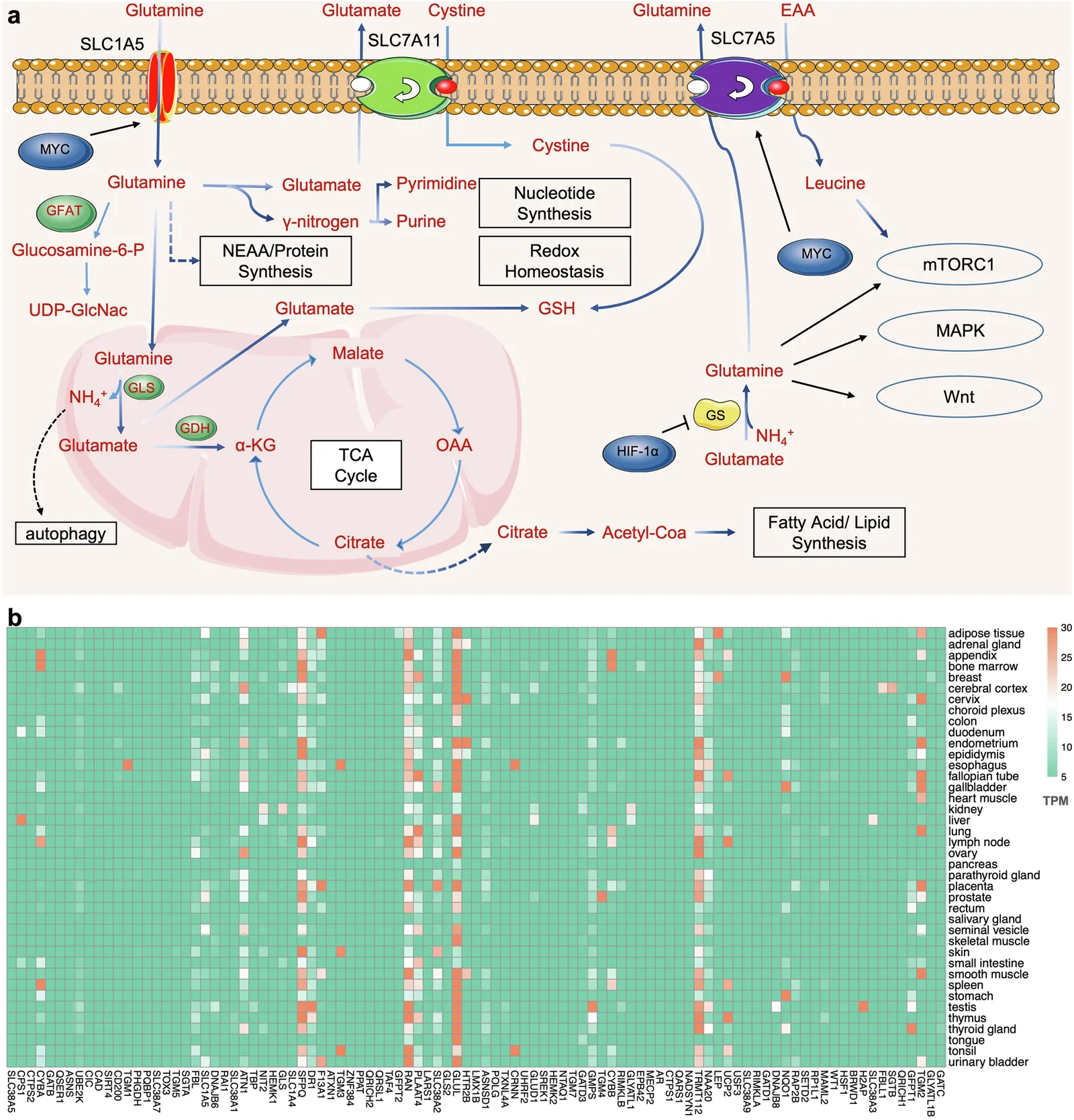
Overview of the glutamine metabolic network in cells and related expression in tissues. **a** Overview of glutamine metabolism in cells. **b** Heatmap of glutamine metabolism-related gene expression levels (transcripts per million, TPM) in tissues (data from The Human Protein Atlas)

Glutamine in the cytosol is imported into mitochondria via a variant of SLC1A5 (SLC1A5_var).^22,25^ Glutamine within mitochondria is involved in maintaining the integrity of the electron transport chain as a protein amino acid.^26^ Mitochondrial glutamine transport is important for rapidly proliferating cancer cells because glutamine is not synthesized in mitochondria.^26^ For glutaminolysis, glutamine is deaminated by GLS to glutamate in mitochondria, which can be further converted to α-KG via glutamate dehydrogenase (GDH) or transaminases.^21^ α-KG replenishes tricarboxylic acid (TCA) cycle intermediates (anaplerosis reactions), fueling ATP production and biosynthesis of lipids and NEAAs (Fig. 1a). The process of converting glutamine into α-KG is essential for the maintenance of the stemness of muscle stem cells and hair follicle stem cells.^27,28^ Deleting GDH caused mitochondrial glutamate accumulation and inhibited the malate-aspartate shuttle in muscle stem cells. High α-KG levels inhibit GDH, whereas ammonia promotes autophagy and compensates for pH imbalance. Ammonium produced during glutamine breakdown increases lysosomal pH and destroys lysosomal function.^1,2^ Excess ammonia within mitochondria can also perturb proton and REDOX balance, leading to severe mitochondrial damage and cellular ammonia death.^14^

GSH is an antioxidant tripeptide in cells. Glutamine is the primary source of GSH and is the rate-limiting factor for GSH de novo synthesis in the cytosol.^6^ Glutamine starvation decreases GSH levels in cells (Fig. 1). Most of the circulating GSH is synthesized in the liver, whereas the kidneys, lungs, and intestines consume most of the circulating GSH.^29^ Additionally, glutamine can be used in other biosynthetic pathways, such as NADPH, and is involved in maintaining cellular integrity and function.^30^ The metabolic fate of glutamine is based on catalysis by specialized subcellular enzymes. The expression levels of glutamine-related genes in tissues are shown in a heatmap based on the data from HPA (The Human Protein Atlas) (https://www.proteinatlas.org/) (Fig. 1b and Table 1).

**Table 1:** Glutamine-related gene expression level (TPM) in tissues (from HPA)

ID	SLC38A5	CPS1	CTPS2	CYBA	GATB	QSER1	ASNS	UBE2K
adipose tissue	0.413866	0.231432	1.562399	8.313336	1.218819	1.004487	0.899276	6.698951
adrenal gland	0.273861	0.046559	2.024387	4.700845	1.445609	0.945385	0.963252	6.136636
appendix	1.944129	0.054307	1.389336	31.90046	0.77423	1.239414	1.978561	8.090356
bone marrow	3.365656	0.019228	1.162786	31.84711	0.504579	0.640259	0.882943	3.668249
breast	0.277916	0.1706	4.592624	2.489143	1.378091	1.780324	1.578446	5.311824
cerebral cortex	2.995478	0.279617	2.044434	3.064801	3.054765	1.460702	4.662402	11.23008
cervix	1.399686	0.0933	3.6272	5.258082	0.765163	1.801786	1.021507	6.169456
choroid plexus	0.220045	0.040996	0.898881	3.836766	0.781124	0.378641	0.588764	1.92476
colon	1.313792	0.07015	2.005247	12.36495	0.908024	0.8115	1.17849	7.700549
duodenum	1.420486	16.31405	3.720982	13.53342	0.625057	0.587337	0.76839	6.789153
endometrium	0.819593	0.293704	4.553404	4.955209	0.843112	2.267798	2.021877	6.066138
epididymis	0.605481	0.429451	3.115168	3.296109	1.521805	1.486652	2.839047	7.154893
esophagus	0.835213	0.13094	1.243353	3.707818	1.194403	1.317708	0.905627	7.109825
fallopian tube	0.746999	0.111911	3.429905	9.212581	1.267244	1.617879	1.096295	4.848656
gallbladder	0.537708	0.197965	2.598099	14.81038	0.771566	1.4778	1.222194	6.059245
heart muscle	0.167306	0.066526	0.675277	1.769814	3.235912	0.596382	0.622605	3.099654
kidney	0.146624	0.155151	1.456998	6.302479	1.949631	0.947856	0.585023	6.915314
liver	0.034576	52.42728	0.867917	1.552786	0.913889	0.483186	0.140556	5.568887
lung	2.120949	0.09912	2.378435	12.46049	0.727874	1.103652	1.305973	5.559713
lymph node	1.110755	0.054035	2.038822	27.19651	0.859115	1.051695	2.437753	6.043468
ovary	0.776841	0.282311	3.380302	2.368228	1.097926	2.232258	1.077212	3.751722
pancreas	5.28547	0.014691	0.404148	1.201935	0.134313	0.227788	1.631295	0.986654
parathyroid gland	0.04677	0.028322	3.728375	0.970796	1.602846	1.399626	2.014944	5.991488
placenta	0.155141	0.28407	2.401129	5.578462	1.108666	1.32033	0.533003	8.456441
prostate	0.694179	0.114849	4.30889	2.715339	0.934005	1.243223	1.699673	6.52674
rectum	1.681573	0.058833	2.942335	6.369247	0.952206	0.990332	1.625921	7.3348
salivary gland	0.429647	0.03415	0.646843	3.406922	0.316889	0.693822	1.084944	1.834496
seminal vesicle	1.263317	0.120398	2.460549	3.452349	1.287617	1.153265	1.407463	5.107449
skeletal muscle	0.039054	0.026252	0.482608	0.918627	0.912412	0.247221	0.153096	2.459208
skin	3.267551	0.231477	4.48182	1.817711	1.72395	2.470251	2.197675	5.298979
small intestine	1.020654	10.37665	2.982162	14.39939	0.773799	0.756609	0.964919	6.314637
smooth muscle	0.867691	0.249323	2.762984	5.762384	0.946887	1.548992	1.842586	6.956771
spleen	0.902337	0.153547	1.778009	33.09109	0.976578	1.389356	1.70461	5.820039
stomach	1.024489	0.064746	1.919796	14.64343	0.612248	0.853305	2.552502	6.565914
testis	0.165886	0.89686	0.991468	1.458218	1.393301	2.815093	3.718163	10.14368
thymus	1.207262	0.04567	1.772839	8.784193	1.423454	1.855033	1.353926	7.246365
thyroid gland	3.27888	0.261096	2.861329	5.783704	1.136784	2.401833	3.10111	8.122857
tongue	0.057275	0.021856	0.386159	0.626363	1.362353	0.478504	0.218582	2.397234
tonsil	0.807284	0.036878	1.936066	13.3345	0.782255	0.975727	1.708272	5.714406
urinary bladder	1.14173	0.043592	1.475875	12.51964	0.747932	1.169609	1.181746	6.639687

ID	CIC	CAD	SIRT4	CD200	TGM1	PHGDH	PQBP1	SLC38A7
adipose tissue	2.079364	1.120345	0.289971	1.901423	0.070856	2.420968	2.173682	0.570177
adrenal gland	1.992599	1.214671	1.279256	2.021404	0.048798	0.149349	3.486826	0.689282
appendix	3.291816	2.202957	0.540982	3.052132	0.13722	0.589899	3.012963	0.717556
bone marrow	2.60284	0.778384	0.067463	0.25193	0.053824	0.358042	2.470625	0.180255
breast	1.031557	1.173523	0.470268	1.503726	0.30036	1.609216	2.795164	0.388539
cerebral cortex	3.623535	1.227543	0.823088	10.17793	0.134833	4.821283	3.693111	1.24332
cervix	2.408766	1.47778	0.557057	4.196405	0.291666	1.429876	3.056401	0.575757
choroid plexus	0.332687	0.408672	0.314454	0.449897	0.033143	0.967459	1.575112	0.129585
colon	1.045144	1.070383	0.367762	0.834632	0.065091	0.497202	2.09048	0.361186
duodenum	1.732855	1.148074	1.171893	0.473521	0.098346	0.3025	3.226073	0.389763
endometrium	4.718498	2.854577	0.960914	7.028689	0.150337	1.253833	4.060329	0.759997
epididymis	0.990887	1.938908	0.356396	1.283707	0.219712	3.578085	2.304539	0.715929
esophagus	1.346457	1.373006	0.509032	1.380505	44.84441	2.417454	2.435759	0.3301
fallopian tube	3.453514	2.098569	0.783231	6.365049	0.734712	3.558286	4.208781	0.487508
gallbladder	2.680365	1.536227	0.634228	3.035467	0.102389	0.48924	2.918135	0.708722
heart muscle	0.755057	0.436853	0.821438	0.4864	0.071513	0.229105	1.546543	0.624428
kidney	0.964303	0.629131	1.191729	0.890531	0.125715	1.648335	1.907827	0.385444
liver	0.456993	0.33348	0.54531	0.071866	0.020811	2.659186	1.21496	0.327074
lung	2.422535	1.360301	0.828395	2.021067	0.500948	0.555029	2.77376	0.582718
lymph node	2.96914	2.30206	0.534887	4.616288	0.188901	0.85793	3.165827	0.476511
ovary	3.358524	1.689794	1.011774	3.241433	0.147441	2.202208	5.616423	0.383093
pancreas	0.467608	0.264921	0.138763	0.138333	0.042197	1.462616	0.652462	0.055634
parathyroid gland	1.496177	0.967062	1.178065	2.043	0.037688	5.693324	2.701761	1.207985
placenta	1.859399	1.3826	0.430666	5.492619	0.198026	0.91056	2.26731	0.881123
prostate	2.222309	1.607849	0.667467	2.853006	0.258265	3.602702	3.204719	0.532751
rectum	0.681683	1.412554	0.395089	1.307868	0.11268	0.446589	2.042749	0.589342
salivary gland	1.038124	0.43884	0.473025	0.45466	0.094624	2.395667	1.320588	0.141179
seminal vesicle	2.685381	1.448048	0.662221	3.222215	0.680232	1.873366	3.173175	0.541167
skeletal muscle	0.755066	0.436211	1.072833	0.083654	0.023043	0.040807	1.475896	0.271204
skin	4.257301	2.721096	1.203095	0.464328	3.189605	5.298618	4.02134	0.556675
small intestine	1.651389	1.016388	0.515806	0.861086	0.088901	0.26478	3.047407	0.39133
smooth muscle	2.316132	2.236646	0.904534	3.869881	0.085044	1.28048	3.127999	0.89086
spleen	3.69223	1.775285	0.548336	1.287029	0.197003	0.177289	3.382865	0.90115
stomach	1.292249	1.016806	0.451976	0.634106	0.070532	0.804935	1.929008	0.397474
testis	5.191123	2.738524	3.261076	0.909346	0.217382	2.374068	1.356557	4.007227
thymus	2.792464	1.88117	0.991223	4.626137	0.196877	2.069301	3.917188	0.404886
thyroid gland	2.229707	1.880242	1.051087	3.890364	0.238925	2.739745	3.101051	1.016238
tongue	0.607927	0.251748	0.93193	0.207023	0.013796	0.078522	1.057151	0.243363
tonsil	1.290608	1.397638	0.330468	3.293608	3.981678	0.703807	2.433871	0.380173
urinary bladder	1.384065	1.045009	0.33923	4.522706	0.088933	0.676512	2.447037	0.471355

ID	TOX3	TGM5	SGTA	FBL	SLC1A5	DNAJB6	RAI1	SLC38A1
adipose tissue	0.061678	0.056072	1.60531	4.902216	17.45662	5.251096	1.013495	0.891416
adrenal gland	0.811787	0	1.665709	5.058994	2.642744	5.088035	1.954673	0.666631
appendix	0.38135	0.024227	1.7553	8.556779	6.667444	5.196698	1.995862	4.821907
bone marrow	0.008032	0.176122	1.564367	7.353291	6.032061	11.01791	0.734782	1.686684
breast	1.01503	0.525938	1.122995	13.34752	8.612868	6.656798	1.121078	9.597583
cerebral cortex	1.961466	0.076027	4.049662	3.045976	0.809079	10.69714	2.80904	6.599053
cervix	2.229862	0.03572	1.653915	8.631685	13.84788	4.254036	2.783615	1.892588
choroid plexus	0.024122	0.013121	0.709557	2.317773	0.804395	1.941867	0.521099	0.111211
colon	3.060897	0.014111	1.24055	7.259122	7.953814	3.539236	0.844159	3.509252
duodenum	1.901153	0.009969	1.975408	4.976432	2.670188	2.758917	1.25748	1.590695
endometrium	0.454354	0.055194	2.231142	11.36895	5.487149	7.668467	2.734958	2.355798
epididymis	1.131756	0.007206	1.52213	9.905159	19.15487	7.986811	0.971052	0.986809
esophagus	0.13031	1.625912	1.865727	8.539354	8.264037	6.72542	1.22026	2.630102
fallopian tube	1.072111	0.023706	2.672181	15.66004	8.76446	5.929178	3.315196	2.329658
gallbladder	2.205754	0.098641	1.815018	5.202718	9.311584	5.678245	2.224035	2.374045
heart muscle	0.006155	0.007693	2.065958	1.780024	0.968781	4.434121	0.784871	2.501649
kidney	2.070311	0.011411	1.20486	3.411753	5.491551	4.624862	0.835275	2.024976
liver	0.533298	0.003775	0.678184	2.283182	0.67509	1.484146	0.306269	0.226817
lung	0.42344	0.065935	1.739456	5.105253	7.593283	5.460698	1.804535	1.797659
lymph node	0.028526	0.042777	1.843577	10.7999	4.9707	4.121504	1.412277	7.04866
ovary	0.162758	0.004577	2.364116	16.49065	7.228517	4.518821	1.158572	0.700933
pancreas	0.115505	0.002829	0.639246	1.273102	2.550201	0.709635	0.372826	0.467602
parathyroid gland	0.010771	0.016863	1.866473	5.692242	0.527632	3.806272	2.416949	8.803754
placenta	0.082182	0.01714	2.342852	5.749152	10.94289	7.220681	1.205436	2.995296
prostate	0.601526	0.094488	2.102161	6.948458	19.39031	5.479267	3.056802	4.374221
rectum	4.030614	0.012372	0.889565	8.704667	7.476442	3.538847	0.716155	3.83843
salivary gland	0.238991	0.166996	1.039334	2.188343	6.181832	1.667914	1.201699	0.796527
seminal vesicle	0.164228	0.397335	2.025304	6.366366	17.30835	5.750343	2.596445	4.522238
skeletal muscle	0.246639	0.013323	1.839193	1.520135	0.228713	7.222038	0.328735	0.219687
skin	0.038502	6.024824	1.398626	11.03281	3.763898	6.383976	2.569636	3.37219
small intestine	2.096734	0.01129	1.967036	5.157589	3.580704	3.563775	1.193585	2.093679
smooth muscle	0.329778	0.025675	1.721411	9.132374	7.77977	6.688284	1.696708	3.6616
spleen	0.023727	0.033529	2.289879	8.209264	5.979424	5.288103	3.043495	4.18552
stomach	4.117515	0.013181	1.375351	4.421622	5.538879	2.775472	1.774078	1.033467
testis	0.259109	0.20649	2.903439	9.442799	7.907427	12.82156	1.77268	0.203474
thymus	0.35962	0.082877	3.382029	12.40273	4.304808	6.273827	1.740243	7.249141
thyroid gland	0.02089	0.015325	2.924643	8.362473	0.821275	5.26795	2.222748	6.472206
tongue	0.044438	0.004183	1.356696	2.204401	0.690958	3.936356	0.145045	2.968847
tonsil	0.045826	0.495099	0.941807	13.3422	4.556512	3.704089	0.923618	4.661711
urinary bladder	1.359606	0.092494	1.445975	7.621386	15.21892	6.554398	1.637504	2.578042

ID	ATN1	TBP	NIT2	HEMK1	GLS	SLC1A4	SFPQ	DR1
adipose tissue	17.29191	1.476993	5.995151	1.705391	3.180786	1.172333	19.01085	7.848256
adrenal gland	19.85076	1.668187	4.734341	2.398122	7.579077	5.615697	18.7898	8.010243
appendix	11.56868	2.474552	4.777143	2.837617	5.214228	2.600219	24.55632	12.82186
bone marrow	1.292991	1.607454	3.468581	0.916992	8.698386	2.431797	61.62323	12.62996
breast	7.379427	1.940858	8.059874	2.365303	3.749797	3.827289	28.88707	6.115696
cerebral cortex	27.43988	2.363221	4.845809	1.781404	9.259602	15.85243	19.1211	9.730474
cervix	14.7218	1.581639	4.111461	2.609711	2.813907	4.343275	21.28847	8.289798
choroid plexus	3.381451	0.501556	1.798506	2.007736	0.97067	0.49658	7.553856	2.719985
colon	6.423703	1.26809	3.938431	1.951404	2.741397	2.042304	13.51918	5.811553
duodenum	9.145143	1.626126	3.5806	2.221775	7.460563	1.460471	13.56494	9.05474
endometrium	23.27403	3.03715	6.955323	3.464943	4.525119	4.638092	39.86123	11.73705
epididymis	7.52946	1.594151	8.128329	2.790423	3.771097	2.356786	32.80327	4.485223
esophagus	8.746138	1.519239	5.419604	1.921597	1.649018	1.601884	11.18412	10.60413
fallopian tube	21.12103	2.594707	6.567815	4.114558	2.615306	1.662063	24.92491	7.573631
gallbladder	17.52197	2.374544	6.414305	2.452013	6.208312	1.4239	23.05692	12.00983
heart muscle	6.469911	0.878551	3.370651	1.252962	1.684421	0.734513	6.207461	5.578954
kidney	5.925151	1.255339	20.01094	1.779093	21.32351	0.413557	12.35096	7.465583
liver	1.478938	0.544131	14.60623	1.145753	0.455073	0.594827	7.504378	3.340919
lung	15.88627	2.113728	5.27902	2.656325	7.063523	1.568984	21.76738	9.730222
lymph node	6.42528	2.987591	5.120645	3.056925	6.728923	2.871911	30.19925	16.52252
ovary	27.631	2.199727	5.354628	3.493497	2.605311	2.998571	22.41481	6.58866
pancreas	2.39224	0.313666	0.863763	0.749594	0.664396	1.00853	3.014098	1.325601
parathyroid gland	16.63577	1.688162	4.80848	3.728759	5.362521	1.353842	17.67207	6.861957
placenta	15.91123	2.601182	4.478554	2.971629	4.403481	2.017728	27.05322	15.10612
prostate	15.9301	2.157966	6.732558	3.980068	3.043586	3.610948	28.90266	9.019173
rectum	5.697855	1.641536	4.874858	2.189711	2.905222	2.029999	20.80382	5.866955
salivary gland	8.196989	0.763027	1.518317	1.643048	0.759246	0.37345	6.16962	2.68687
seminal vesicle	20.79284	1.685653	5.881963	3.146139	3.234544	2.098323	18.85303	6.310271
skeletal muscle	4.110262	0.514245	3.365077	0.515885	0.363398	2.836092	4.207694	5.613431
skin	13.91278	2.548685	9.474241	1.982353	2.937275	4.785239	36.59851	10.49258
small intestine	9.276624	1.910461	3.165891	2.213873	8.770498	1.472507	15.82898	8.207731
smooth muscle	17.82593	2.347329	5.951213	2.993568	4.505777	3.757279	24.94782	9.111547
spleen	11.47298	2.601089	4.006097	3.186831	5.609401	2.312782	26.55113	12.21285
stomach	8.314918	1.468134	3.929004	2.436713	1.505293	1.749077	14.04001	9.544128
testis	6.465507	9.423664	3.396208	4.074183	2.566478	3.334857	29.28416	33.11536
thymus	5.353718	2.54757	4.19159	3.627295	4.364059	5.51167	33.64482	20.52193
thyroid gland	20.17199	2.625853	8.641144	4.861219	5.431199	2.086221	24.4516	10.19498
tongue	3.353543	0.651826	2.446837	0.768528	0.65433	1.358303	2.895373	5.557293
tonsil	3.237917	1.908964	4.736012	1.937031	4.208385	3.668909	23.65594	9.290953
urinary bladder	9.089907	1.709129	5.079115	1.70822	3.225272	1.215255	18.17196	10.45486

ID	F13A1	ATXN1	TGM3	ZNF384	PPAT	FOXP2	QRICH2	QRSL1
adipose tissue	46.7762	0.536169	0.051189	2.172805	0.83756	0.095033	0.150349	1.841225
adrenal gland	12.52262	0.580871	0.069607	2.576346	1.694158	0.099859	0.146286	2.342338
appendix	11.80804	1.182064	0.176806	3.872221	1.735954	0.130021	0.228535	3.829233
bone marrow	1.617692	2.371687	0.496679	3.060025	1.521074	0.008814	0.283774	1.695657
breast	9.34236	0.656911	2.594003	1.769801	1.937764	0.10979	0.152345	3.138146
cerebral cortex	1.649023	1.343287	0	2.96407	2.156608	0.220292	0.35818	3.271103
cervix	13.28212	1.040284	0.328531	2.696062	1.490382	0.220845	0.173648	2.46321
choroid plexus	1.681108	0.23837	0.003585	0.484267	0.323396	0.007003	0.119918	1.386516
colon	7.298526	0.531021	0.156314	1.883776	0.934656	0.206495	0.095914	2.812988
duodenum	1.602129	0.522709	0.028029	2.895448	0.909726	0.130879	0.064415	2.793156
endometrium	5.289519	1.693761	0.10942	4.751473	1.915047	0.648337	0.316696	3.632501
epididymis	1.098377	0.613049	0.843	2.090994	0.982952	0.015951	0.244378	2.40497
esophagus	9.152249	0.663282	762.3105	1.777172	1.118871	0.296693	0.201826	2.10639
fallopian tube	2.315998	0.763521	0.592511	3.833561	1.633699	0.290927	0.460229	2.966994
gallbladder	13.64831	1.21562	1.743699	3.027301	1.288799	0.255885	0.225959	2.830887
heart muscle	4.401421	0.326734	0.106541	1.108296	0.326692	0.032766	0.096016	2.128537
kidney	3.411384	0.446368	0.003032	1.655776	1.373525	0.090132	0.089972	3.20782
liver	0.238445	0.390257	0.035567	0.518781	0.654752	0.058672	0.053349	1.306977
lung	9.426921	1.053809	0.041456	2.78792	0.806373	0.112461	0.395377	2.329398
lymph node	5.05458	0.729072	0.028561	3.293921	2.389871	0.023628	0.228605	6.374009
ovary	2.989082	0.682111	0.028106	3.301321	1.889467	0.321718	0.154067	2.107986
pancreas	0.34698	0.130233	0	0.458641	0.291735	0.047243	0.064693	0.422326
parathyroid gland	2.914393	0.684837	0.004076	3.099934	1.03397	0.014659	0.208723	3.774831
placenta	114.6824	0.887861	0.113584	3.302332	2.212113	0.046988	0.397594	2.778345
prostate	1.533642	1.041008	0.319065	3.109206	1.42928	0.164978	0.329109	2.9803
rectum	11.61336	0.601282	0.424294	2.598605	1.295091	0.304945	0.118057	3.341099
salivary gland	0.609752	0.226235	0	0.916944	0.523508	0.059233	0.52066	0.87908
seminal vesicle	2.998571	0.842177	0	2.714258	0.947568	0.100142	0.215932	2.575739
skeletal muscle	0.192401	0.402664	0.061583	0.831666	0.226917	0.034519	0.086557	2.179615
skin	4.364352	0.768374	29.23892	2.602006	1.539867	0.185588	0.33295	2.344834
small intestine	4.177911	0.559729	0.116587	3.070707	0.989045	0.164283	0.165826	2.348786
smooth muscle	20.487	1.217418	0.046743	3.744325	1.239049	0.526342	0.182305	2.913121
spleen	1.406461	1.048241	0.106508	3.732005	1.471902	0.036245	0.27362	4.87958
stomach	3.84914	0.606522	0.040478	2.138532	0.835978	0.054812	0.096103	2.156729
testis	5.84503	0.695278	0.259752	3.991365	3.747895	0.118011	3.82913	4.201494
thymus	4.537102	0.439679	0.088467	4.254761	2.437505	0.019358	0.211605	3.801337
thyroid gland	3.016853	1.077561	0.01186	3.571653	2.240055	0.44583	0.680293	5.38799
tongue	2.137773	0.439495	0.006185	0.842265	0.239757	0.04452	0.055448	2.759274
tonsil	1.949608	0.468343	64.54725	2.52182	1.696203	0.032708	0.10908	4.745903
urinary bladder	26.53545	0.805423	0.243461	2.174034	1.715136	0.21183	0.08475	2.260753

ID	TAF4	GFPT2	RAN	PLAAT4	LARS1	SLC38A2	GLS2	GLUL
adipose tissue	0.485981	11.82295	20.8701	5.513405	1.266379	12.60634	0.031592	195.8371
adrenal gland	0.552522	0.760213	25.18052	4.319328	1.397754	13.59602	0.266553	21.52568
appendix	0.860594	3.87042	39.24592	15.53469	1.786393	11.08156	0.05099	68.00384
bone marrow	0.473505	0.035069	21.60426	2.864737	0.611958	12.28763	0.026925	45.37815
breast	0.169538	1.484797	21.89723	27.195	2.178653	11.3759	0.115827	43.01512
cerebral cortex	0.777769	1.976569	23.89426	9.659436	2.051488	5.022069	0.742533	127.8255
cervix	0.323432	1.720875	17.25988	14.27015	1.597463	13.91871	0.07389	48.23309
choroid plexus	0.066919	1.014503	11.3995	5.506253	0.765885	0.3662	0.21212	24.8325
colon	0.478898	0.491593	21.42393	9.875281	1.235709	5.570821	0.065362	20.04322
duodenum	0.874296	0.227852	15.15626	18.80929	0.807264	3.609737	0.033303	6.361505
endometrium	0.818667	2.547519	21.43138	10.27198	2.317145	17.77269	0.128841	31.20575
epididymis	0.326825	1.249303	23.79381	15.24708	1.781057	4.356112	0.304752	20.43151
esophagus	0.451107	0.857585	24.76743	4.481358	1.021226	12.77235	0.161538	73.00896
fallopian tube	0.424684	1.445809	23.73776	32.74255	1.888964	10.94694	0.466059	45.88991
gallbladder	0.450137	4.003827	19.90718	17.40701	1.188667	24.05078	0.057899	41.98394
heart muscle	0.152659	0.346602	8.884962	1.761612	0.687659	2.823147	0.023984	14.41223
kidney	0.377282	0.284936	16.49686	13.25002	1.196229	6.391005	0.140529	13.16908
liver	0.217096	0.154345	7.432466	10.81312	0.423478	14.17494	6.509533	29.2955
lung	0.559479	1.132763	19.69448	29.45151	1.384031	8.910217	0.185057	60.41981
lymph node	0.466183	0.435946	40.94859	22.74948	1.486072	9.52059	0.083068	19.43825
ovary	0.762793	1.971022	22.62637	18.55048	1.389885	15.04121	0.066536	62.05672
pancreas	0.110446	0.363221	2.549118	0.298053	0.329238	1.62762	0.337851	11.64438
parathyroid gland	0.391914	0.67461	19.24571	0.575742	1.563911	11.42176	0.485631	8.310364
placenta	0.522933	1.477772	28.93331	5.148588	1.391263	28.6417	0.016437	62.81892
prostate	0.40514	1.789891	18.5539	12.1629	1.880059	8.841307	0.222057	20.74662
rectum	0.321635	0.474726	24.26649	13.09938	1.57216	6.893097	0.130564	22.09057
salivary gland	0.199922	0.254328	4.200389	8.059619	0.689991	3.341675	0.050355	7.104406
seminal vesicle	0.304156	1.534845	17.62849	21.87798	1.432812	6.649601	0.250192	27.15889
skeletal muscle	0.38793	0.195519	8.1444	0.838456	0.715382	3.00765	0.006919	29.65415
skin	0.598424	0.433969	14.16004	2.028128	1.287735	24.92614	0.379781	11.45005
small intestine	0.86056	0.363626	17.36051	23.57652	0.972482	4.643273	0.063214	11.26652
smooth muscle	0.267788	2.28237	29.69274	13.24367	2.153856	18.1606	0.062622	45.0357
spleen	0.89282	0.219055	21.90773	20.06323	1.620737	11.90464	0.097823	43.99653
stomach	0.452576	0.250382	14.63139	12.6081	0.987309	6.23587	0.094372	68.4738
testis	2.162377	1.87279	45.64939	3.287882	1.722384	4.545399	0.123711	64.13608
thymus	0.688248	0.242376	62.70631	22.54123	1.656869	13.58297	0.165168	31.52077
thyroid gland	0.329945	0.71335	19.545	8.491012	2.358268	10.62719	0.213028	91.06499
tongue	0.073312	0.381643	12.47409	1.365485	0.821214	6.023344	0.007035	46.57422
tonsil	0.196413	0.425875	36.22783	11.22967	1.29048	7.238424	0.116588	21.42262
urinary bladder	0.211172	5.399695	36.18876	13.5147	1.267304	10.82783	0.05281	46.14834

ID	HTR2B	LMX1B	ASNSD1	SLC38A6	POLG	TXNL4A	CACNA1A	CRNN
adipose tissue	1.159011	0.051453	7.332865	0.757783	1.024455	4.801445	0.048234	0.035119
adrenal gland	18.97857	0.021707	8.22309	0.670355	0.903029	6.160091	0.165177	0.014873
appendix	1.984	0.024035	6.425838	0.739266	1.354677	5.78852	0.055392	0
bone marrow	0.773078	0.021172	5.706565	0.262348	0.986312	3.939305	0.074922	0.004712
breast	0.636341	1.683084	9.922067	0.695273	0.828481	4.165024	0.030278	0.221335
cerebral cortex	0.229744	0.036528	9.957374	0.598342	0.960488	7.889682	0.720237	0
cervix	31.3961	0.009352	10.32203	0.637398	0.688881	4.031449	0.018132	12.73991
choroid plexus	0.731397	0.024535	2.58196	0.395476	0.363902	4.833736	0.033091	0
colon	1.605886	0.017811	4.429136	0.408208	0.839473	4.135343	0.048146	0.119301
duodenum	1.5067	0.031059	5.586414	0.373884	0.863049	3.489323	0.145686	0
endometrium	30.82542	0.009664	11.7093	0.893312	1.048309	3.911596	0.055435	0.210224
epididymis	13.9204	0.024655	5.748	1.143536	0.760639	6.330207	0.142257	0
esophagus	0.853327	0.01198	5.653201	0.290345	0.996844	4.152654	0.029889	4330.517
fallopian tube	1.368793	0.03704	9.540303	0.775607	0.822232	4.678665	0.116108	0.035929
gallbladder	1.96532	0.023398	7.592933	1.355612	1.170957	4.160342	0.065249	1.794227
heart muscle	2.452478	0.204348	4.897264	0.207664	0.433124	3.174766	0.018506	0.887753
kidney	3.44422	0.419137	7.781395	0.794445	0.486975	3.887706	0.019259	0.006103
liver	1.737428	0.000395	4.872211	0.285538	0.321284	2.076358	0.007069	0.098376
lung	1.381504	0.05201	8.064635	1.021435	1.249033	3.930308	0.035217	0.007325
lymph node	1.197535	0.010553	7.599838	0.845128	1.644809	6.506787	0.065422	0.005408
ovary	0.779134	0.008621	10.33352	0.498826	0.652178	2.451624	0.027412	0.001662
pancreas	0.022683	0.013028	1.414708	0.062716	0.156157	1.359544	0.043899	0
parathyroid gland	0.643467	0.359935	9.528803	1.597806	0.822392	4.311229	0.016252	0
placenta	5.730606	0.009962	11.77518	1.102841	1.190176	4.607312	0.035476	0
prostate	0.539209	0.024767	8.490325	0.730224	0.826805	4.751817	0.035903	0.003342
rectum	2.300697	0.015663	4.927241	0.57516	1.006819	3.757984	0.074854	0.01591
salivary gland	0.2135	2.537605	2.561507	0.189981	0.299267	2.211427	0.021115	0
seminal vesicle	1.893372	0.006851	7.002293	0.410321	0.77176	3.741458	0.026848	0.017402
skeletal muscle	0.04418	0.007935	4.997182	0.148041	0.468871	2.886788	0.017409	0.009159
skin	0.221135	0.272784	5.043272	0.437104	1.617867	4.611077	0.066621	4.947363
small intestine	1.186105	0.025849	4.813132	0.343307	1.001986	3.980311	0.120405	0
smooth muscle	23.5988	0.02751	10.07054	0.670691	1.093209	4.299252	0.023483	0.26215
spleen	0.636626	0.013369	7.813262	0.918781	1.632266	4.420689	0.04937	0.031073
stomach	0.605392	0.037457	4.601315	0.384137	0.736488	3.495664	0.29451	0
testis	0.857414	0.5622	8.02857	1.584527	0.826371	7.671191	0.250005	0.180956
thymus	1.591056	0.010526	9.05145	0.579325	2.095722	6.399061	0.020745	0.058183
thyroid gland	1.348197	0.060939	10.72481	0.855065	0.788668	5.529261	0.063927	0.045456
tongue	0.146017	0.00246	6.835697	0.125638	0.433406	2.816417	0.009551	0
tonsil	0.768999	0.003866	5.669184	0.367938	1.035922	4.340526	0.021993	222.3023
urinary bladder	2.607857	0.007215	7.774438	0.593816	0.890701	4.226543	0.019848	0.554488

ID	LGSN	UHRF2	GLUD1	SREK1	HEMK2	NTAQ1	TGM7	KALRN
adipose tissue	0.016342	1.524857	3.518169	2.252856	1.668522	0.978281	0	0.303058
adrenal gland	0.034027	1.617325	5.268011	2.768509	2.814877	1.892811	0.005867	0.294141
appendix	0.027758	3.178022	2.895854	3.385607	1.498218	1.196317	0	0.189287
bone marrow	0.042594	6.505235	0.884583	8.086041	0.344074	0.546384	0	0.024841
breast	0.012874	2.475984	5.562521	3.648297	2.855176	2.014472	0.014061	0.139828
cerebral cortex	0.018649	1.76591	11.77267	2.602527	3.42347	1.932606	0	1.692721
cervix	0.014657	2.494754	3.32489	2.970337	2.62828	2.063236	0.004102	0.190103
choroid plexus	0.00719	0.44719	1.901046	0.905169	1.084245	0.980199	0.274695	0.313702
colon	0.065555	1.402971	2.889123	1.83101	1.310723	0.97792	0.003131	0.448335
duodenum	0.01209	1.528655	3.786415	2.3384	1.464298	0.57736	0	0.973093
endometrium	0.046471	3.863898	3.574629	5.580739	4.521204	1.559897	0.047332	0.3901
epididymis	0.027727	2.932703	1.796109	4.813667	3.001248	1.309957	0	0.132685
esophagus	0.007413	1.180773	3.642947	2.173638	1.202992	1.029385	0.133163	0.449034
fallopian tube	0.062875	2.042506	3.528452	3.010601	3.660271	1.367101	0.002433	0.281455
gallbladder	0.038933	2.295118	3.774266	3.813743	1.747623	1.328356	0	0.510093
heart muscle	0.006631	0.567307	2.104852	1.096837	1.518137	1.472154	0	0.447714
kidney	0.503667	1.119212	11.44326	1.924583	1.954186	1.339335	0.024638	0.134844
liver	0.46544	0.572585	18.8339	1.001496	0.667044	0.468824	0	0.248123
lung	0.109274	2.160107	4.034261	2.952443	1.98827	1.233862	0.000974	0.142592
lymph node	0.027185	4.869648	2.24479	3.645119	1.896808	1.138205	0.004501	0.086005
ovary	0.01667	2.207251	4.09574	2.162999	3.277076	2.441428	0.041249	0.101099
pancreas	0.015656	0.361122	0.646372	0.58519	0.267634	0.276971	0	0.057223
parathyroid gland	0.010825	1.743584	2.011833	3.194769	2.735033	1.309184	0	0.157448
placenta	0.389476	2.852357	3.906192	3.773243	3.525738	1.070559	0.001554	0.357851
prostate	0.082709	4.060701	8.185949	4.11198	3.035609	2.017472	0.202558	0.206571
rectum	0.037718	2.093142	3.146647	2.647082	1.808958	1.07869	0	0.498492
salivary gland	0.01014	0.584691	0.85229	1.085588	0.649163	0.331574	0.063875	0.030483
seminal vesicle	0.006925	2.201365	3.366843	2.640243	2.297956	1.296152	0.042148	0.394434
skeletal muscle	0.016664	0.408317	0.747787	0.644	1.127232	0.726314	0.014147	0.443035
skin	0.051467	2.133481	3.788339	4.853006	1.73413	1.071172	0	0.070226
small intestine	0.018069	1.763003	4.594365	2.330897	1.628914	0.668101	0	1.101065
smooth muscle	0.048716	2.766001	3.657691	3.187322	2.540119	1.54648	0.008434	0.395643
spleen	0.04468	3.552537	2.959386	3.635231	2.542637	1.094718	0	0.471238
stomach	0.034416	1.751144	2.346628	2.514777	1.402423	0.656852	0	0.590091
testis	0.146682	3.115609	1.839463	3.957522	2.825529	3.149185	3.488885	0.560359
thymus	0.015434	4.38386	2.657107	3.369993	1.367818	1.212236	0.012896	0.032899
thyroid gland	0.017246	2.238426	5.187777	5.863838	3.267588	2.497429	0	0.387776
tongue	0.001518	0.445109	1.154272	0.573832	1.002657	0.90511	0	0.440415
tonsil	0.020858	3.093503	2.512599	2.44567	1.22215	1.119191	1.159364	0.096466
urinary bladder	0.007612	1.615262	3.603655	2.001196	1.253918	1.226024	0.038015	0.121683

ID	GATD3	ATXN7	GMPS	TGM4	CYBB	RIMKLB	GLYATL1	EPB42
adipose tissue	6.019242	0.722923	7.679257	0.019681	8.543053	4.932422	0.006426	0.561782
adrenal gland	7.151938	0.778782	8.996252	0.506691	6.379705	2.086069	0.013079	0.008907
appendix	4.633464	1.47105	10.69807	0.161558	38.0271	1.637923	0.046159	0.008077
bone marrow	1.878281	1.880951	11.53199	0.053261	29.3454	1.630758	0.015723	10.55263
breast	4.324481	0.769317	10.49009	0.098786	3.108835	2.716766	0.655195	0.085043
cerebral cortex	7.328833	0.745252	7.111853	0.001417	4.586968	4.3943	0.075577	0
cervix	4.221087	0.838183	10.69121	0.003182	3.112343	3.468498	0.03077	0
choroid plexus	2.734034	0.096208	2.917618	0	0.746669	1.826214	0.044926	0.003936
colon	4.459086	0.612346	7.342597	0.002692	4.041736	0.923919	0.042776	0.008747
duodenum	5.618589	0.635643	6.710717	0.012268	3.414656	1.006228	0.121375	0.003065
endometrium	4.374454	1.360651	12.07327	0.002914	2.818532	11.69217	0.036509	0
epididymis	4.367729	0.648135	6.277007	0.293074	1.005329	3.964153	0.041237	0
esophagus	4.555645	0.753798	9.227332	0.005408	1.80366	1.794403	0.030586	0.011713
fallopian tube	5.822162	0.755614	8.331476	0.000887	2.869811	6.484706	0.139083	0.003526
gallbladder	4.759405	1.310659	8.972517	0.002913	7.207836	5.351628	0.172786	0.154215
heart muscle	8.554473	0.288547	4.323374	0.001162	0.944486	0.99603	0.010032	0.0158
kidney	11.77603	0.607646	5.797209	0.007042	1.51305	1.280789	20.50483	0.001522
liver	5.777892	0.356422	4.289534	0.003651	2.32327	0.234163	14.80559	0.005066
lung	4.287019	1.146294	9.068044	0.062511	23.99299	4.004025	0.0056	0.010697
lymph node	5.796204	1.405023	12.15052	0.00588	20.20137	2.049003	0.114863	0.002625
ovary	5.606633	0.522123	7.509636	0.001012	1.227205	3.685451	0.00163	0.011647
pancreas	0.912154	0.339806	2.246416	0.002983	0.16782	0.207539	0.019559	0.002415
parathyroid gland	5.928129	1.111431	10.40948	0.006596	0.938753	4.809412	0.305056	0
placenta	4.670897	1.111355	11.46284	0.013156	13.65159	9.193253	0.006459	0.854314
prostate	5.742347	0.964775	9.476181	189.6795	2.085775	3.237032	1.62252	0
rectum	4.202411	0.990779	9.396919	0.000469	6.222025	1.333305	0.035101	0.005347
salivary gland	2.342872	0.268656	3.196929	0.001446	0.578247	0.805039	0.06876	0.004547
seminal vesicle	4.14972	0.567104	8.308681	0.016444	2.249115	3.160108	0.007483	0.00485
skeletal muscle	6.412575	0.236398	2.066093	0.003008	0.246586	0.459247	0.002182	0.002456
skin	4.432536	0.957103	8.511245	0.017416	0.599753	1.903539	0.071372	0.016297
small intestine	4.900285	0.849714	6.715673	0.043306	4.607149	1.5436	0.035858	0.003886
smooth muscle	3.500699	0.942688	13.05012	0.000804	13.93438	5.460211	0.014365	0
spleen	5.903442	1.260108	9.760234	0.013324	22.04073	3.426671	0.022295	0.023007
stomach	3.823506	0.673579	5.867693	0.002749	2.913168	1.191112	0.194425	0
testis	4.384582	1.815479	72.95464	0.240865	1.77437	8.489354	0.056256	0.647545
thymus	4.20607	1.651088	16.80737	0.016251	4.176686	2.184874	0.213466	0.045824
thyroid gland	6.473361	1.113957	11.41898	0.008891	1.728814	4.938911	0.014363	0.010641
tongue	10.1712	0.202762	2.739317	0	0.364051	0.377489	0.001039	0.039277
tonsil	3.397683	0.852271	10.60968	0.000657	12.52579	1.047794	0.017103	0
urinary bladder	3.253542	0.657651	10.69457	0.000389	8.960155	1.35129	0.006791	0.006986

ID	TGM6	MECP2	AR	KYAT1	CTPS1	QARS1	NADSYN1	TRMT112
adipose tissue	0	0.572026	0.901041	0.384591	0.658759	1.681161	1.878592	18.3926
adrenal gland	0	0.693505	0.196088	0.472164	0.885071	1.673867	2.162543	39.0277
appendix	0	0.807163	0.105607	0.587352	1.129972	2.443479	3.345822	24.51206
bone marrow	0.002035	2.442796	0.012839	0.241347	0.784203	2.196112	4.224595	15.30449
breast	0.003073	0.484275	1.517402	0.70728	0.439793	2.869344	2.01691	19.58662
cerebral cortex	0	0.767756	0.152253	1.970641	0.558977	1.3141	1.737201	19.18672
cervix	0	0.642437	4.859792	0.418119	0.765788	2.11238	2.164191	22.91512
choroid plexus	0	0.256062	0.038023	0.927012	0.28475	1.219707	1.090283	19.30402
colon	0.001035	0.360243	0.104076	0.189374	0.523058	3.434849	3.050109	12.41693
duodenum	0	0.457715	0.044634	0.321709	0.414831	2.719919	3.550294	11.28741
endometrium	0	0.997106	4.221514	0.78719	2.641666	2.471926	2.816159	33.6128
epididymis	0	0.637053	3.882147	0.495795	0.516303	2.633893	3.392311	27.78407
esophagus	0	0.454863	0.184379	0.394103	0.908916	2.79452	2.362191	21.12538
fallopian tube	0.001524	0.877656	4.907122	1.013075	0.958461	2.828796	2.745283	44.16888
gallbladder	0	0.772813	0.459283	0.546544	1.06764	2.227198	3.665277	22.943
heart muscle	0	0.301806	0.66243	0.620658	0.183086	1.146106	0.717356	10.75906
kidney	0	0.411666	0.511847	0.671739	0.744771	1.642037	1.220153	17.20849
liver	0.000709	0.142547	2.601423	0.451997	1.795507	0.706896	0.676609	9.18714
lung	0	0.767692	0.325932	0.450264	0.642699	2.263786	2.768342	23.00082
lymph node	0.001635	0.718852	0.056449	0.789803	0.964844	2.636672	3.734527	31.332
ovary	0.001301	0.770157	2.240283	0.565813	0.346789	2.663128	1.792719	40.38216
pancreas	0	0.112948	0.03199	0.086604	0.081732	0.380557	0.341347	3.829343
parathyroid gland	0	0.68465	0.024121	0.850531	2.739885	2.728895	1.622964	22.34391
placenta	0	0.539172	0.031448	0.511763	0.746096	2.749544	2.405591	26.23241
prostate	0.005068	0.867671	3.717251	1.070569	1.360623	2.753483	2.835325	27.25991
rectum	0	0.287884	0.100021	0.224631	0.73045	3.568481	3.429213	11.743
salivary gland	0	0.234603	0.051466	0.303909	1.247334	1.190696	0.950682	8.442822
seminal vesicle	0	0.881246	3.552466	0.828039	1.809928	2.393828	2.970318	21.76812
skeletal muscle	0	0.380828	0.58634	0.510134	0.187759	1.49467	0.251684	7.192113
skin	0	0.519181	0.920691	1.487766	0.405117	3.435395	2.890278	19.18641
small intestine	0	0.508039	0.05677	0.501884	0.645613	2.548945	3.94875	13.2059
smooth muscle	0	0.715548	1.988425	0.688184	3.621476	2.395634	2.530768	25.26659
spleen	0.002206	0.920638	0.259761	0.798863	0.547827	2.544643	4.198926	27.05047
stomach	0	0.420573	0.078834	0.320143	0.344683	2.011912	2.250803	12.67352
testis	0.012808	0.705816	0.997915	1.082307	1.773001	2.136301	2.796543	27.75172
thymus	0.002989	0.847661	0.506757	1.458486	1.205056	4.025385	2.998096	32.94474
thyroid gland	0	0.802325	0.517617	1.085202	0.449749	2.491134	2.321457	30.49011
tongue	0	0.201225	0.307421	0.402972	0.132343	1.73392	0.197168	5.303697
tonsil	0.150062	0.42257	0.068799	0.590479	0.893749	2.821041	2.291359	23.029
urinary bladder	0.002048	0.537023	0.389294	0.295687	1.124668	2.623384	2.952623	21.97448

ID	NAA20	HAP1	LEP	UCP2	USF3	SLC38A9	RIMKLA	GATD1
adipose tissue	9.029755	0.013717	304.4778	8.107797	1.537536	0.524156	0.070838	1.227539
adrenal gland	11.32949	0.27346	1.135987	2.760117	0.868359	0.329235	0.161026	2.714506
appendix	8.550206	0.06531	7.136273	20.76423	1.010172	0.601249	0.277684	1.737079
bone marrow	4.981829	0.012115	0.100347	9.028753	1.567776	0.307366	0.021398	0.550024
breast	12.64103	0.146772	28.13876	4.664079	1.592987	0.469938	0.158188	0.978299
cerebral cortex	10.64039	1.587411	0.166506	2.410783	1.145292	0.469245	4.469237	3.547429
cervix	9.221456	0.225227	0.095836	6.106011	1.395492	0.464268	0.119181	0.987985
choroid plexus	7.674231	0.127268	0.003992	3.385729	0.526822	0.064604	0.222026	0.807738
colon	6.502933	0.061446	2.033528	3.99808	1.102047	0.461398	1.470301	0.994646
duodenum	5.184662	0.392232	0.145276	5.529592	0.706835	0.413307	0.163674	1.618707
endometrium	12.01205	0.519328	0.124948	3.180872	1.065801	0.641483	0.125966	1.987784
epididymis	11.55976	0.278312	0	0.932417	2.315937	0.403336	0.042823	1.472694
esophagus	21.61906	0.037107	0.217569	2.039009	0.929187	0.415846	0.100461	1.464482
fallopian tube	11.76916	0.28866	0.096555	28.13466	1.121153	0.370159	0.09509	1.794764
gallbladder	9.789665	0.324758	1.997358	7.386637	0.987373	0.641927	0.406204	1.468464
heart muscle	7.063995	0.005626	0.340248	0.910752	0.499085	0.113988	0.970471	0.641997
kidney	13.32801	0.043834	0.081408	2.244172	1.592698	0.409481	0.401971	1.015015
liver	6.251687	0.02955	0.019825	1.320914	1.379505	0.360701	0.009024	0.287355
lung	10.16499	0.110693	0.112722	9.909254	1.287835	0.64049	1.420262	1.349268
lymph node	7.842931	0.042246	0.237324	33.73368	1.492752	0.652227	0.060901	1.348524
ovary	7.480044	0.13772	0.051081	0.604936	0.895016	0.358048	0.323657	1.471248
pancreas	2.251901	0.029912	0.523158	0.262944	0.153155	0.121591	0.085856	0.327196
parathyroid gland	17.36511	0.007143	0.019001	1.019076	1.906388	0.581614	1.243652	1.309572
placenta	12.46321	0.304026	6.348901	4.000711	1.00746	3.483493	0.193427	1.419362
prostate	11.14501	0.143554	0.082911	3.170009	1.390687	0.52318	0.460022	1.872426
rectum	7.59379	0.076379	0.504921	5.369171	1.046628	0.46308	1.700905	0.655964
salivary gland	3.71958	0.106525	0.451993	1.81378	0.557755	0.141932	0.040222	0.562209
seminal vesicle	9.697566	0.256444	0.040028	2.999823	0.927206	0.379849	0.148243	1.520277
skeletal muscle	4.530245	0.012411	0.176317	1.870574	1.569147	0.14735	0.056084	0.480383
skin	8.851607	0.102428	0.263285	1.203805	2.052815	0.603302	0.117539	1.846842
small intestine	6.31956	0.182663	0.243442	5.363624	0.904015	0.402035	0.149707	1.694154
smooth muscle	11.15724	0.138402	0.177486	4.589224	0.885096	0.559366	0.482574	1.035402
spleen	7.274392	0.339113	0.340747	25.81451	1.593071	0.739458	0.157675	1.827314
stomach	6.4503	1.791686	0.050488	5.514005	0.891241	0.436713	0.292886	1.387547
testis	20.39295	0.449566	0.464592	3.021319	1.330282	1.789938	0.620085	2.066367
thymus	7.346551	0.529598	0.465082	30.12034	2.179682	0.559108	0.05177	1.253349
thyroid gland	17.67527	0.898001	0.143409	7.867247	1.703198	0.619737	0.508683	1.73916
tongue	4.280981	0.00577	0.481384	5.790592	0.827231	0.073153	0.221068	0.253292
tonsil	8.026481	0.034187	0.030778	21.90775	1.071106	0.587734	0.038725	0.704745
urinary bladder	9.341535	0.035622	0.164156	9.170683	0.833654	0.449274	0.093054	0.882586

ID	PFAS	DNAJB8	NQO1	RAP2B	SETD2	RP1L1	MAML2	WT1
adipose tissue	1.066969	0.002354	14.41372	5.38248	0.772106	0.174148	4.83028	0.395194
adrenal gland	0.921117	0.027142	6.14729	2.60519	0.765204	0.216908	1.773412	0.011787
appendix	1.666339	0	4.055629	8.14759	0.956021	0.307072	4.203987	0.117385
bone marrow	0.918732	0.00889	0.085894	9.267855	1.396586	0.497925	1.055178	0.011939
breast	0.737912	0	47.83214	2.894013	1.047904	0.255173	8.229728	0.013187
cerebral cortex	1.186203	0	7.021608	7.09688	0.868746	0.418744	7.409411	0.008957
cervix	0.98142	0	5.226318	5.53627	1.217942	0.128125	7.861041	0.53989
choroid plexus	0.150477	0	12.26569	0.686892	0.468465	0.090654	0.726548	0.001872
colon	0.831465	0	11.90887	3.648048	0.726873	0.121147	2.937729	0.023798
duodenum	0.696557	0	15.42117	2.604045	0.781153	0.22833	1.50797	0.006037
endometrium	1.40033	0	3.146254	4.977284	1.36073	0.245674	4.429728	6.602143
epididymis	0.697277	0	4.552178	3.438	1.456448	0.28414	1.08492	0.794758
esophagus	0.915059	0	13.85445	8.662767	0.607526	0.203398	2.9367	0.001293
fallopian tube	1.397096	0	6.575841	3.9065	1.130889	0.264839	6.448973	7.239287
gallbladder	0.656844	0	40.62753	9.853413	0.864252	0.269344	5.962132	0.198291
heart muscle	0.308162	0	0.914409	1.088405	0.291907	0.114671	1.289237	0.094541
kidney	0.656106	0.00171	7.955401	1.942377	0.849338	0.236487	5.001004	0.576765
liver	0.288582	0	0.231048	2.161463	0.488144	0.044714	0.593775	0.00668
lung	0.867137	0.001111	4.444044	6.927077	1.089929	0.266615	7.248767	0.065049
lymph node	1.89054	0	0.815304	6.977404	1.220843	0.389804	5.000642	0.006845
ovary	0.883965	0	1.514114	6.345803	1.121144	0.16108	5.473286	3.022568
pancreas	0.209133	0	0.217541	0.524748	0.185013	0.034017	0.61852	0.004971
parathyroid gland	1.080863	0	3.340885	2.48617	1.445958	0.217628	3.895376	0.006477
placenta	1.187069	0.001285	2.499	12.03253	1.09969	0.309893	8.592768	0.220958
prostate	1.092392	0.00246	4.706255	3.484547	1.232183	0.277859	5.946173	0.051111
rectum	1.066139	0	11.35693	3.839123	0.95649	0.228754	3.342192	0.006596
salivary gland	0.298812	0	2.036411	0.889107	0.336711	0.075324	2.332927	0.002219
seminal vesicle	0.884496	0.00306	4.934037	3.225687	1.042291	0.121038	3.850383	0.475145
skeletal muscle	0.258204	0.004297	0.770975	0.448356	0.31078	0.15179	0.178325	0.001383
skin	1.072814	0	0.935145	5.226013	1.299753	0.431254	3.030652	0.005911
small intestine	0.85045	0.005072	8.548915	3.296965	0.831846	0.214536	1.81538	0.007926
smooth muscle	1.460818	0	7.159129	4.515023	1.214303	0.134808	4.745208	4.775193
spleen	1.379168	0	2.647305	10.13074	1.244131	0.560011	7.057067	1.27415
stomach	0.584442	0	70.99623	8.029643	0.670984	0.171062	2.187171	0.003389
testis	1.295614	17.61217	1.76293	2.730665	1.789927	0.463167	2.165743	3.035789
thymus	1.429132	0	0.81254	6.13797	2.048371	0.309971	8.894443	0.019259
thyroid gland	1.125595	0	18.55413	2.696156	1.55681	2.291366	5.367861	0.004998
tongue	0.172993	0	1.491266	0.661232	0.359037	0.094946	0.939971	0.018832
tonsil	1.239448	0	2.091982	4.604168	0.945487	0.237095	3.220119	0.002063
urinary bladder	0.894728	0	6.953135	9.320048	0.820642	0.160012	5.018766	0.034502

ID	HSF1	BRWD1	MAGI2	H2AP	SLC38A3	FBLL1	HTT	SGTB
adipose tissue	1.876922	0.933361	0.115319	0	0.196897	0.106588	0.569292	2.442451
adrenal gland	1.991983	1.539406	0.204019	0	0.026243	3.876707	0.572159	1.693447
appendix	2.252959	0.968269	0.056831	0	0.028821	0.185689	0.785414	5.176523
bone marrow	1.765347	0.851017	0.057553	0	0.064324	0.00849	1.052135	3.123562
breast	1.131296	1.426815	0.101774	0.032903	0.190735	0.325321	0.581029	1.461874
cerebral cortex	1.986832	4.667491	1.106094	0	3.931938	21.23277	1.04073	25.0709
cervix	1.722128	1.301836	0.131128	0	0.063316	0.257725	0.492263	2.271757
choroid plexus	0.689184	0.346127	0.048511	0	0.905239	0.290546	0.246708	0.249847
colon	1.471297	0.764778	0.065517	0.037192	0.033872	0.126528	0.515008	1.02676
duodenum	1.426526	0.738281	0.063808	0	0.151129	0.452726	0.391611	0.733772
endometrium	2.518011	1.701229	0.200358	0.070424	0.068436	0.128533	0.650681	3.87775
epididymis	1.116572	1.756268	0.146598	0	0.073171	0.094466	0.932933	1.182935
esophagus	2.37484	0.893255	0.073932	0	0.227506	0.078885	0.642301	1.597764
fallopian tube	2.857732	1.471647	0.141979	0	0.133052	0.688943	0.674864	1.427558
gallbladder	2.362435	1.174388	0.131435	0	0.110826	0.427431	0.62608	2.367459
heart muscle	1.705337	0.63582	0.059904	0	0.514416	0.217248	0.237968	1.286681
kidney	0.96304	1.064491	0.174911	0	0.46259	0.275172	0.52417	1.319465
liver	0.504828	0.821846	0.02863	0	19.37947	0.077197	0.197616	0.631549
lung	2.072098	1.178089	0.131491	0	0.044311	0.161667	0.881977	2.884262
lymph node	2.008708	1.311006	0.070265	0	0.095324	0.049224	0.918384	4.020547
ovary	2.064362	1.28179	0.321152	0	0.747289	0.45759	0.447201	2.027077
pancreas	0.472719	0.315212	0.022618	0	1.002829	0.171975	0.180575	0.191927
parathyroid gland	1.951734	1.019718	0.482616	0	1.158961	0.565864	0.866598	1.467689
placenta	2.22016	1.901462	0.065412	0.023905	0.288327	0.298228	0.776999	2.616085
prostate	2.025387	1.527584	0.368534	0	0.132214	0.671271	0.683608	3.83537
rectum	1.083751	0.926386	0.060739	0	0.01382	0.114232	0.539611	1.387118
salivary gland	0.860023	0.325362	0.02353	0	0.159267	0.194236	0.153693	0.315077
seminal vesicle	2.117011	1.075986	0.286521	0	0.044154	0.482053	0.489094	1.420262
skeletal muscle	1.51923	0.384227	0.025393	0	2.855675	0.044133	0.250707	0.448449
skin	1.974366	0.989689	0.091548	0.271521	0.096996	0.565066	1.333307	1.183557
small intestine	1.699457	0.880895	0.078734	0	0.088141	0.594466	0.51566	0.806309
smooth muscle	2.0456	1.087301	0.103505	0.070255	0.055091	0.322372	0.543672	3.837482
spleen	2.325941	1.09022	0.102615	0	0.029098	0.10769	0.926915	3.200381
stomach	1.404635	0.743029	0.058643	0	0.346993	0.386863	0.430353	0.82833
testis	5.758585	2.809541	0.664791	43.19859	0.354987	4.610699	1.211128	1.740827
thymus	1.913115	1.321295	0.030023	0	0.014433	0.098437	0.829827	2.692611
thyroid gland	2.759712	2.321758	0.293879	0	0.236233	0.169872	1.079755	3.44625
tongue	1.018995	0.640457	0.023779	0	1.952035	0	0.323798	0.920074
tonsil	1.111802	0.967171	0.019243	0	0.038144	0.011907	0.557564	2.253673
urinary bladder	1.906802	0.927949	0.08432	0	0.006333	0.371725	0.495892	2.267479

ID	QRICH1	GFPT1	TGM2	GIGYF2	ATXN2	GLYATL1B	GATC	ARHGAP11B
adipose tissue	1.596139	6.949464	26.06364	0.504062	0.518555	0.000239	3.956753	0.224755
adrenal gland	1.903516	4.604131	5.691951	0.605856	0.667013	0.035077	4.157692	0.191393
appendix	2.328949	5.865308	19.88627	0.672744	0.55866	1.130546	4.155101	0.723578
bone marrow	2.430581	1.943017	1.231885	0.554802	0.289635	0.048938	1.955588	0.98605
breast	2.085853	5.333337	0.933091	0.667988	0.412852	9.278825	5.203636	0.080548
cerebral cortex	1.922894	4.150541	3.110771	0.780762	0.996758	0.033613	6.717696	0.336522
cervix	2.355849	11.38144	81.38751	0.64369	0.605258	0	5.706692	0.136873
choroid plexus	0.649088	2.306835	6.027141	0.119631	0.2677	0	1.219915	0.029269
colon	1.46352	14.52762	4.713028	0.467466	0.37869	0.034909	3.594536	0.185021
duodenum	1.530648	7.719421	7.359277	0.503484	0.403264	0.110826	4.548998	0.281934
endometrium	2.92795	5.279358	60.13811	0.870941	0.861803	0	5.541137	0.32012
epididymis	2.351205	9.846949	16.8763	0.501373	0.645951	0	4.349778	0.331998
esophagus	1.796374	3.49274	8.038741	0.587168	0.392593	0	3.087194	0.282953
fallopian tube	2.391061	7.625886	62.08071	0.665187	0.786498	0.017317	4.629348	0.166683
gallbladder	2.355455	10.1027	40.86121	0.619539	0.639372	0.181222	4.188414	0.353446
heart muscle	0.886842	3.082663	25.02596	0.356605	0.306729	0	1.299482	0.127786
kidney	1.274391	4.54649	4.072432	0.61011	0.414394	0.004551	5.551278	0.146886
liver	0.687754	2.323289	6.319529	0.189265	0.23394	0.024193	2.878066	0.037745
lung	2.607787	5.076299	48.97795	0.597872	0.699406	0.036081	4.304541	0.286547
lymph node	2.886641	2.436699	4.541832	0.605322	0.436889	3.414234	4.680684	0.974912
ovary	2.142691	2.665088	3.143405	0.612704	0.656278	0.005944	4.293915	0.139655
pancreas	0.322419	1.414798	1.002482	0.128269	0.075674	0	3.803177	0.042439
parathyroid gland	2.289973	6.406316	4.193301	1.135651	0.62484	0	5.41198	0.049209
placenta	2.251543	10.09034	72.74144	0.737029	0.816003	0	4.767124	0.263806
prostate	2.373149	11.79012	18.84805	0.820708	0.771742	1.130374	5.02239	0.215033
rectum	2.094351	16.30136	5.516555	0.525416	0.444532	0.028949	4.149177	0.3013
salivary gland	0.692819	3.332014	0.586219	0.281412	0.164356	0.081606	1.598514	0.091159
seminal vesicle	2.284207	7.11261	16.26862	0.5265	0.643613	0	3.856292	0.024139
skeletal muscle	0.634609	0.704094	1.163419	0.309493	0.239523	0	0.953077	0.075328
skin	2.441895	1.48438	0.19135	0.678087	0.528787	0.279753	4.080959	0.47706
small intestine	1.774276	9.12604	9.991232	0.580158	0.376698	0.199475	4.289805	0.285542
smooth muscle	2.548103	6.651688	43.8447	0.633091	0.791468	0	4.153093	0.181779
spleen	2.948665	2.702774	5.97448	0.722295	0.600029	0.810228	4.427405	0.393235
stomach	1.395854	12.73763	3.39454	0.491036	0.377002	0.023977	3.395627	0.231088
testis	4.384715	16.94418	2.25075	1.772591	1.182915	0.277314	3.077053	0.692606
thymus	3.905201	2.669199	3.55309	0.748405	0.558325	0.097306	5.81587	0.619876
thyroid gland	2.296222	29.7955	2.814264	1.030497	0.76799	0	6.970619	0.948096
tongue	0.688567	1.083582	2.438542	0.274811	0.197461	0	1.078751	0.003854
tonsil	2.707083	2.922441	2.139971	0.454683	0.285167	0.073134	3.850693	0.351027
urinary bladder	1.758325	4.973274	19.18144	0.527017	0.543514	0.039004	4.074846	0.197813

ID	KYAT1
adipose tissue	0.350454
adrenal gland	0.219289
appendix	0.266675
bone marrow	0.335078
breast	0.343132
cerebral cortex	1.010281
cervix	0.306797
choroid plexus	0.309139
colon	0.173811
duodenum	0.28406
endometrium	0.413435
epididymis	0.547134
esophagus	0.267049
fallopian tube	0.390469
gallbladder	0.308268
heart muscle	0.103043
kidney	0.126606
liver	0.158727
lung	0.2316
lymph node	0.399502
ovary	0.370131
pancreas	0.058856
parathyroid gland	0.183083
placenta	0.448379
prostate	0.495109
rectum	0.176676
salivary gland	0.086898
seminal vesicle	0.387673
skeletal muscle	0.185551
skin	0.522787
small intestine	0.156678
smooth muscle	0.285778
spleen	0.329081
stomach	0.131752
testis	0.706082
thymus	0.447901
thyroid gland	0.303868
tongue	0.183164
tonsil	0.260444
urinary bladder	0.178818

### Synthesis of glutamine

As a conditional EAA, glutamine can be synthesized in vivo. In general, the body’s requirement for glutamine is not met by intake, and extensive glutamine is synthesized de novo in the body.^31^ As shown in Fig. 1b, glutamine synthetase (GS) is expressed in most tissues.^32,33^ Glutamine synthesis occurs primarily in skeletal muscles, adipose tissue, lungs, liver, erythroblasts, and the brain via GS, which incorporates ammonia into glutamate (Fig. 1a). This reaction consumes ATP and is essential for ammonia detoxification and glutamate-glutamine cycling in astrocytes. As a conditional EAA, glutamine cannot be synthesized in certain conditions, such as stress or illness.

### Transporters and cellular uptake

While the intracellular fate of glutamine is determined by core enzymes such as glutaminase and glutamine synthetase, its entry into the cell is the critical first step governed by specialized plasma membrane transporters. Cellular uptake of glutamine is not a passive process but an actively regulated event involving several SLC families, with SLC1A5, SLC7A5, and SLC38 being paramount.

The SLC1A5 transporter, also known as ASCT2 (alanine-serine-cysteine transporter 2), is a sodium-dependent neutral amino acid exchanger that primarily facilitates the uptake of glutamine, alanine, serine, and cysteine.^3,34^ It operates with a high affinity for glutamine, making it a principal entry point for this amino acid into many cell types, particularly those with high anabolic demand. The functional importance of SLC1A5 extends beyond mere nutrient uptake; it serves as a critical component of a broader amino acid network. By importing glutamine, SLC1A5 provides substrate for subsequent metabolic pathways and, importantly, fuels antiporter systems involving other transporters. Its expression is frequently upregulated in rapidly proliferating cells, including cancer cells.^3,16^ This upregulation is not merely a passive response but can be driven by oncogenic signaling pathways, positioning SLC1A5 as a key mediator of metabolic adaptations.

In contrast to the sodium-dependent symport mechanism of SLC1A5, the SLC7A5 transporter (LAT1, L-type amino acid transporter 1) functions as a sodium-independent exchanger, typically in a heterodimeric complex with its heavy chain subunit SLC3A2 (CD98hc, 4F2hc).^16,34^ Glutamine imported via SLC1A5 can then be rapidly effluxed in exchange for extracellular EAAs, notably leucine, via the SLC7A5/SLC3A2 heterodimer.^3^ Imported leucine is a potent activator of the mechanistic target of rapamycin complex 1 (mTORC1) signaling pathway, a master regulator of cell growth and proliferation. Thus, the SLC1A5/SLC7A5 axis forms a critical metabolic nexus, translating extracellular glutamine abundance into intracellular anabolic signaling.^3,16^ This cooperative mechanism underscores why targeting glutamine uptake can disrupt broader growth signaling networks beyond mere glutamine deprivation.

The SLC38 family of transporters represents another major class of sodium-coupled neutral amino acid transporters with diverse substrate specificities and regulatory profiles. Members such as SLC38A1 (SNAT1) and SLC38A2 (SNAT2) are considered system A transporters, exhibiting broad selectivity for small neutral amino acids, including glutamine, alanine, serine, and asparagine. They are highly pH-sensitive and subject to complex regulation by amino acid availability, hormonal signals, and cellular stress, often through transcriptional and posttranscriptional mechanisms such as substrate-induced stabilization or degradation.^34^ Their role extends beyond constitutive nutrient supply; they are integral to adaptive responses. For instance, SLC38A2 is notably responsive to amino acid starvation, with its expression upregulated as part of the amino acid response pathway to compensate for nutrient deficiency. In specific tissues, SLC38 transporters fulfill specialized roles. In the nervous system, members such as SLC38A1 are crucial for the glutamate-glutamine cycle, facilitating glutamine export from astrocytes to neurons.^35^ In the intestinal epithelium, glutamine absorption is mediated by SLC38 transporters in crypt cells, highlighting their role in organ-specific homeostasis (Fig. 1b, Table 1).^36^

The above transporters are far more than simple conduits; they are dynamic, regulated gatekeepers whose expression and activity integrate extracellular nutrient status with intracellular metabolic and signaling destiny, playing decisive roles in both physiological homeostasis and pathological progression.

### Glutamine metabolism and cell signaling pathways

Glutamine plays a significant role in cellular signaling pathways, acting both as a signaling molecule and a regulator of metabolic pathways that influence cell growth, proliferation, and survival. It influences various pathways, including the mTORC1, hexosamine biosynthesis, and MAPK pathways, impacting cellular processes such as protein synthesis, gene expression, and redox balance.

#### mTORC1 pathway

Glutamine positively regulates the mTORC1 pathway, a master regulator of cell growth and metabolism. It facilitates the uptake of leucine, an EAA, which is crucial for mTORC1 activation.^37^ Glutamine metabolism catalyzed by GLS also promotes the assembly and lysosomal localization of mTORC1, further enhancing its activity.^38^ Glutamine deficiency activates the panamino acid receptor, the protein kinase GCN2, which inhibits the mTORC1 pathway.^39^ At the cellular level, MYC and mTORC1 upregulate GLS1, GDH, and glutamine transporters, enhancing glutaminolysis.^40–42^ Hypoxia-inducible factor (HIF-1α) suppresses GS, favoring catabolism under low oxygen.^43^ Glutamine metabolism affects autophagy through the AMPK-mTORC1 signaling axis but plays different roles in promoting or inhibiting autophagy in different cell types and states (Fig. 1).^3,44^ It exhibits heterogeneous inhibition and activation of the mTORC1 pathway in tumor cells but plays an important role in astrocyte maturation and brain nerve development through the mTORC1 pathway, which may be related to glutamine dependence.^42,45–48^ Recent studies have found that hyperglutaminolysis accelerates aging through the arginine-mTORC1 pathway.^38^

#### MAPK pathway

Glutamine can increase mitogen-activated protein kinase phosphatase-1 (MKP-1) expression to negatively regulate the MAPK pathway, which plays a crucial role in reactive oxygen species (ROS) production.^49^ Glutamine attenuates inflammation via MKP-1-dependent deactivation of cPLA2, while deficiency causes an increased number of neutrophils, which is a more severe inflammation than eosinophils.^50,51^

#### Wnt pathway

Glutamine promotes differentiation of intestinal stem cells and proliferation of epithelial cells through the Wnt/β-catenin pathway. This pathway has also been associated with increased glutaminolysis during liver fibrosis. During bone formation, Wnt signaling increases the expression of glutamine transporters and glutaminase, increasing glutaminolysis to meet the needs of osteoblast differentiation, which cascades independent of β-catenin but seems to mTOR after binding the Fzd receptor.^52^ Overall, Wnt signaling activates glutamine catabolism, and glutamine activates Wnt signaling, which plays a role in physiological and pathological processes.

#### Other signaling pathways

Glutamine can activate amino acid-sensitive GPCRs and mobilize calcium from the endoplasmic reticulum via the Gq signaling pathway. It can also influence the expression of various genes involved in metabolism, signal transduction, cell defense, autophagy, and repair. Glutamine-dependent signaling can also impact cell cycle progression and apoptosis.^53^

### Systemic regulation

Glutamine metabolism is tightly regulated at multiple levels. In the whole body, hormones regulate glutamine metabolism, including thyroid hormone,^54^ growth hormone,^55^ insulin,^56^ and glucocorticoids.^57^ Altered GS expression levels and activity have been found to be associated with a variety of physiological processes and diseases, including erythropoiesis,^58^ immunoregulation, aging,^41^ and tumorigenesis.^42^

The intricate regulation of cellular glutamine uptake and metabolism is embedded within a broader, organ-spanning network essential for systemic homeostasis. For instance, the rhythmic expression of SLC6A19 regulates the intestinal absorption of glutamine, which shapes the diurnal sleep-wake cycle.^59^ The body orchestrates glutamine flux between tissues to support anabolic demands, maintain acid‒base balance, and, critically, detoxify ammonia, a pervasive metabolic byproduct.^23^ This systemic perspective reveals that glutamine is not merely a fuel for proliferating cells but a central currency in interorgan nitrogen metabolism, with the gut–liver axis serving as its principal clearinghouse. The maintenance of circulating glutamine levels, or glutaminemia, is a dynamic process controlled by key metabolic organs, including skeletal muscle, the gut, liver, and kidneys.^5^ Skeletal muscle represents the primary reservoir, releasing glutamine into the bloodstream, particularly during catabolic states, to supply other tissues. This release is not passive; it is influenced by local metabolic crosstalk, as demonstrated by the finding that macrophages can adapt their glutamine metabolism to support muscle regeneration by secreting glutamine for uptake by satellite cells via SLC1A5.^60^ The liver and intestines are pivotal in the first-pass metabolism of dietary nitrogen and glutamine. The gut mucosa itself is a major consumer of glutamine, utilizing it for energy and nucleotide synthesis, which underscores its role in maintaining intestinal barrier function.^5^ The ammonia generated from intestinal bacterial metabolism and glutamine catabolism is then delivered via the portal vein to the liver, initiating a critical detoxification sequence central to the gut-liver axis.^61–63^

Hepatic ammonia detoxification is a master class in metabolic zonation and efficiency, relying on the complementary actions of the urea cycle and GS.^61,64,65^ The liver acinus is functionally divided: periportal hepatocytes express high levels of GLS and the full complement of urea cycle enzymes, constituting a high-capacity, low-affinity system for ammonia removal via ureagenesis. In contrast, a small population of perivenous hepatocytes exclusively expresses GS, forming a high-affinity scavenger system that captures any ammonia that escapes periportal detoxification before blood enters the systemic circulation.^61,66^ This anatomical arrangement creates an intercellular glutamine cycle, where periportal glutamine breakdown (yielding ammonia for urea synthesis) is balanced by perivenous glutamine resynthesis, optimizing ammonia clearance at physiological portal concentrations.^61^ The pivotal role of hepatic GS is quantitatively significant; it contributes to approximately 35% of enteral ammonia detoxification, matching the contribution of urea cycle enzymes under certain conditions.^65^ Its importance is further highlighted by therapeutic strategies demonstrating that augmenting hepatic GS expression via gene therapy can effectively lower blood ammonia in models of hyperammonaemia.^67,68^ This GS activity is sensitive to pathophysiological regulation; oxidative stress can inhibit it through tyrosine nitration in a pH-sensitive manner, while hyperammonemia itself can downregulate its expression in astrocytes via Hippo-YAP signaling, creating a vicious cycle in hepatic encephalopathy.^66,69,70^ Beyond classic glutamine synthesis, hepatic GS also exhibits alternative activity, producing N5-methylglutamine from glutamate and methylamine, a metabolite sustained by the microbiome and linked to liver cancer, further intertwining hepatic nitrogen metabolism with gut-derived substrates and pathology.^71^

The kidneys and peripheral tissues, particularly muscle, are integral to systemic ammonia handling, especially when hepatic function is compromised. Renal ammonia metabolism is fundamental to acid‒base homeostasis, with the proximal tubule being a major site of glutamine-driven ammoniagenesis via phosphate-dependent GLS.^72,73^ The ammonia generated is subsequently excreted in the urine, carrying protons. However, the proximal tubule also expresses GS, which recycles a portion of this ammonia back into glutamine, thereby fine-tuning net acid excretion.^72,73^ Deletion of proximal tubule-specific GS increases basal ammonia excretion, underscoring its role as an ammonia-recycling enzyme, while its absence impairs the adaptive increase in ammonia excretion during metabolic acidosis.^72^ This renal process is coordinated by transporters such as the electrogenic Na^+^-bicarbonate cotransporter NBCe1; combined deletion of its A and B splice variants in the proximal tubule causes severe metabolic acidosis and completely blocks the ammoniuretic response to an acid load, demonstrating its critical role in coupling acid‒base status to ammonia generation.^74^ In the periphery, muscle becomes a crucial site for ammonia detoxification via GS during liver failure, highlighting the body’s reliance on extrahepatic GS activity when the primary hepatic systems are overwhelmed.^64,65^ The coordinated action of peripheral (including muscle and perivenous hepatocytes) GS for glutamine synthesis and periportal hepatic GLS for its subsequent breakdown represents the body’s high-affinity ammonia detoxification system.^65^ This interorgan teamwork ensures that glutamine serves as a nontoxic nitrogen carrier, shuttling ammonia from peripheral sites to the liver and kidneys for ultimate disposal.

The gut-liver axis, therefore, is the epicenter of systemic nitrogen and ammonia balance. Dietary protein and intestinal microbial metabolism constantly generate ammonia, which is absorbed into the portal circulation.^62,63^ The liver’s first-pass clearance of this ammonia load is paramount to prevent systemic hyperammonemia, a neurotoxic condition. Impairment in this axis, as seen in advanced liver diseases such as cirrhosis or metabolic dysfunction-associated steatotic liver disease (MASLD), leads to increased ammonia spillover into the systemic circulation.^63,64^ This hyperammonemia has dire consequences, most notably hepatic encephalopathy, where ammonia disrupts astrocyte function, cerebral metabolism, and neurotransmission.^64,69^ Astrocytic GS is the brain’s primary defense, rapidly converting ammonia and glutamate to glutamine. However, chronic hyperammonaemia can dysregulate this enzyme, both by potentially inhibiting its activity and by downregulating its expression via mechanisms such as Hippo-YAP pathway activation, thereby exacerbating neurotoxicity.^69,70,75^ The resulting astrocyte swelling and neuronal dysfunction underscore the systemic reach of a disrupted gut-liver axis. Moreover, evidence suggests that hyperammonaemia can modulate autophagy.^62^ Genetic variations in the *GS* gene have also been associated with altered plasma ammonia levels in patients on medications such as valproic acid, indicating that individual differences in this detoxification pathway can influence drug tolerability and toxicity.^76^

The systemic regulation of glutamine and ammonia is a complex, multiorgan endeavor. The gut-liver axis acts as the primary filter for dietary and microbial nitrogen, employing a zonated hepatic strategy of urea synthesis and glutamine synthesis for efficient detoxification.^61,65^ The kidneys and muscle provide essential backup and fine-tuning mechanisms, particularly for acid‒base balance and during hepatic insufficiency.^65,72,73^ Dysregulation of this homeostasis, whether through liver disease, renal dysfunction, or inherited metabolic disorders, leads to hyperammonaemia with profound clinical implications, driving ongoing research into therapies that target GS activity, modulate interorgan flux, or support alternative ammonia disposal pathways.^67,68,77^

## Glutamine metabolism in neoplastic diseases

### Mechanisms of glutamine dependence and metabolic reprogramming in tumors

While systemic glutamine homeostasis ensures nitrogen balance and supports vital physiological functions, cancer cells exploit this abundant nutrient through profound metabolic reprogramming.^78^ The best documented metabolic alteration is the activation of aerobic glycolysis, the Warburg effect, which activates fatty acid biosynthesis and glutamine consumption, termed “glutamine addiction”.^79^ The Warburg effect promotes tumor development by facilitating a GPT2-dependent linkage between the generation of pyruvate and glutaminolysis.^54^ This identifies a metabolic weakness that is specifically linked to the progression of cancer cells toward malignancy.^80–83^

**Fig. 2:**
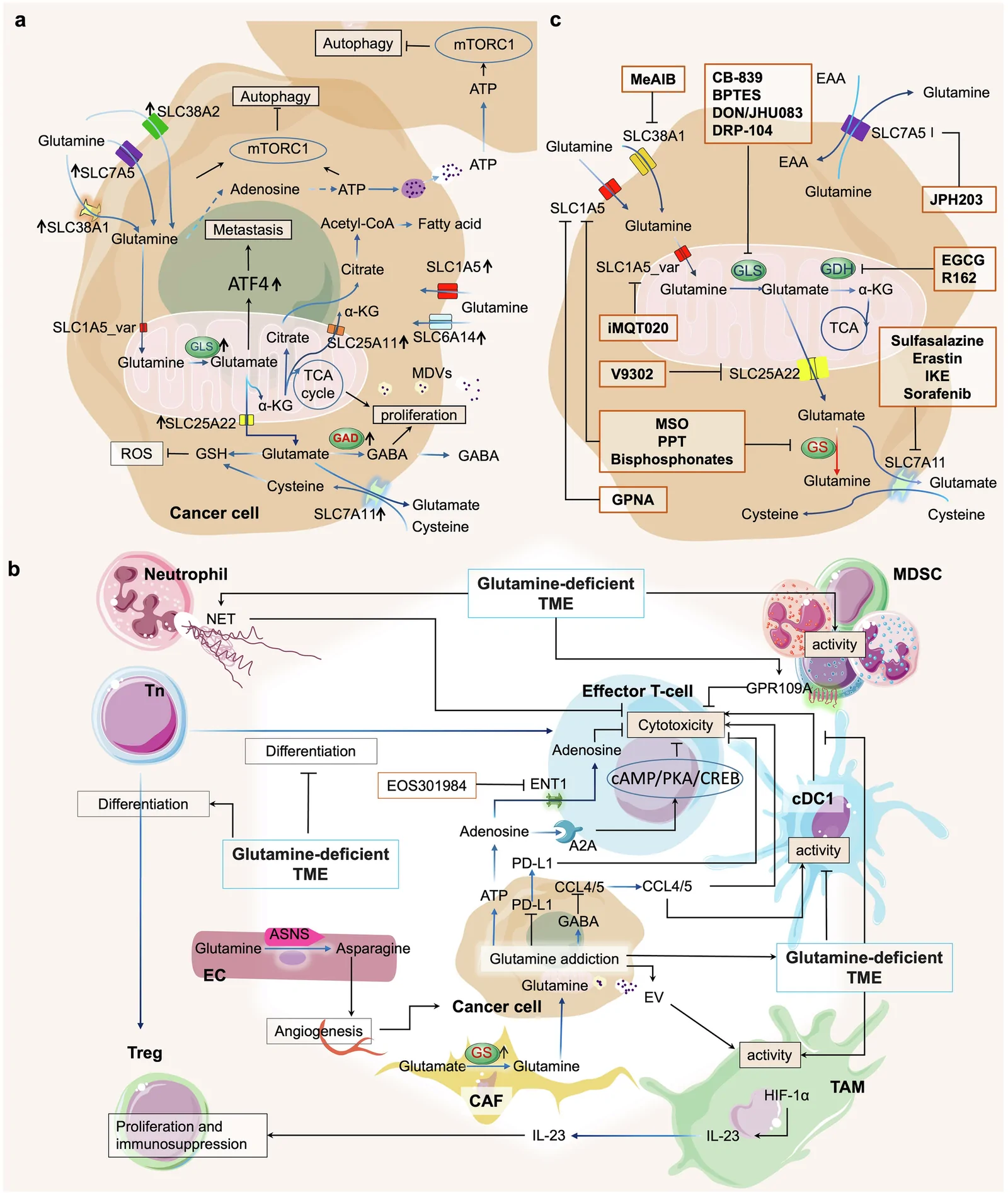
Glutamine addiction of cancer cells, its effect on microenvironment and related inhibitors. **a** Cancer cells upregulate glutamine transporters and glutaminolytic enzymes, triggering glutamine deprivation in microenvironment. Rapid glutamine catabolism fuels cancer proliferation and metastasis by supplying energy and biosynthetic precursors; the resulting metabolites activate oncogenic signaling and suppress oxidative stress and autophagy-mediated cell death. Moreover, glutamine metabolic byproducts are secreted into the tumor microenvironment via specialized channels, cellular vesicles and MDVs, driving malignant activation of cancer cells and mediating immune suppression. **b** Glutamine metabolism and tumor microenvironment. Cancer cells can inhibit effector T-cell and increase immunosuppressive cells, include TAM and cDC1, through glutamine-related metabolites and signaling pathways. Glutamine-deficient microenvironment promotes the differentiation, proliferation and activation of immunosuppressive cells. Glutamine deficiency inhibits toxic T-cell activation. Neutrophils inhibit T cell activation via NETs. Tn differentiate into immunosuppressed Treg cells rather than effector T cells. Glutamine-deficiency increases MDSC infiltration. Glutamine-deficient tumor microenvironment promotes the activity of TAM cells, inhibits the function of cDC1 cells, and jointly enhances the immunosuppressive effect. EC promote the growth of vascular sprouts by converting glutamine to aspartate. CAF supply cancer cells by promoting glutamine synthesis. **c** Extracellular glutamine is imported via multiple amino acid transporters, which can be suppressed by MeAIB, JPH203, GPNA and V9302. The cysteine-glutamate exchanger SLC7A11 transports extracellular cysteine in exchange for intracellular glutamate, and its function is antagonized by sulfasalazine, erastin, IKE, and sorafenib. Glutamine synthetase (GS) reversibly synthesizes glutamine from glutamate, with GS activity inhibited by MSO, PPT and bisphosphonates. Mitochondrial transporters SLC25A22 mediate mitochondrial shuttling of glutamine-derived glutamate. Glutaminase (GLS) inhibitors, including CB-839, BPTES, DON and DRP-104, as well as the SLC1A5_var modulator iMQT020, target core glutamine metabolic enzymes. MeAIB α-(methylamino) isobutyric acid, EGCG epigallocatechin gallate, GPNA γ-L-glutamyl-p-nitroanilide, GLS glutaminase; GS glutamine synthetase, GDH glutamate dehydrogenase, TCA tricarboxylic acid cycle, MSO L-methionine-DL-sulfoximine, DON 6-diazo-5-oxo-L-norleucine, JHU083 Ethyl 2-(2-Amino-4-methylpentanamido)-DON, IKE imidazole ketone erastin, BPTES bis-2-(5-phenylacetamido-1,3,4-thiadiazol-2-yl) ethyl sulfide, GABA γ-aminobutyric acid; MDVs: Mitochondrial-derived vesicles, cDC1s conventional type 1 dendritic cells, TME tumor microenvironment, Tn naive T-cell, Treg regulatory T-cell, NETs Neutrophil extracellular traps, MDSC myeloid-derived suppressor cell, EC endothelial cells, CAF carcinoma-associated fibroblasts

The initial and crucial step in glutamine addiction is the upregulation of specific transporters on the cancer cell membrane (Fig. 2a). The sodium-dependent neutral amino acid transporter SLC1A5 is frequently overexpressed in cancers and serves as a primary gateway for glutamine import (Fig. 2a).^84–86^ Its expression is often driven by oncogenic transcription factors such as c-Myc and N-Myc, creating a feed-forward loop where MYC amplifications directly enhance glutamine influx to support MYC-driven anabolic programs.^84,87^ In hepatocellular carcinoma (HCC), the tRNA methyltransferase TARBP1 promotes the efficient translation of SLC1A5 mRNA, thereby enhancing glutamine uptake and tumor progression^85,86^ Similarly, in gastric cancer, integrin α3 (ITGA3) promotes tumor progression by activating STAT3 signaling, which in turn transcriptionally upregulates SLC1A5 to increase glutamine uptake.^88^ Beyond SLC1A5, other transporters play context-specific roles. SLC7A5 is essential for KRAS-mutant colorectal cancer.^89^ In SMARCA4/2-deficient cancers, loss of these chromatin remodelers represses glucose uptake, forcing cells to rely on increased SLC38A2-mediated glutamine import to fuel oxidative phosphorylation (OXPHOS).^90^ Other amino acid transporters related to glutamine metabolism, such as SLC6A14 and SLC7A11, are also at the forefront of oncology research.^91–93^ Mitochondrial glutamine metabolite transporters also play a role in tumors, such as SLC25A11 or SLC25A22, which export α-KG and glutamate, respectively.^94,95^ This orchestrated upregulation of glutamine transporters ensures a steady intracellular supply, which is then rapidly metabolized by key enzymes to enter central carbon metabolism.^83,96^

The committed step of glutaminolysis is catalyzed by GLS, which deamidates glutamine to glutamate. There are two paralogous *GLS* genes, *GLS1* (or *GLS*) and *GLS2*.^97^ GLS1 is widely recognized for its oncogenic properties and is frequently upregulated in cancers.^81,98^ The role of GLS2 is more complex, while historically viewed as a tumor suppressor, evidence in triple-negative breast cancer (TNBC) shows that it can be protumorigenic, sustaining proliferation and underlying metastasis, particularly upon therapeutic pressure targeting GLS1.^98,99^ Recent studies have shown that aging-ATF4 axis-driven lung cancer hypermetastasis is also dependent on glutaminolysis.^100^

Glutamate can be transaminated to α-KG to replenish the TCA cycle, a process critical in cancers where the m6A reader IGF2BP2 stabilizes transcripts of glutamine metabolism genes to fuel this pathway.^101^ In prostate cancer, radioresistant cells and cancer stem cells exhibit high glutamine demand, with GLS-driven catabolism supporting energy production and the maintenance of redox balance.^102^ Alternatively, under conditions of mitochondrial dysfunction or hypoxia, glutamine-derived α-KG can undergo reductive carboxylation, a reverse TCA cycle flux that generates citrate for lipid synthesis.^103,104^ In HCC, deficiency in enzymes such as SLC25A15 or OGDHL promotes reductive glutamine metabolism, driving lipogenesis and providing energy and building blocks for tumor progression.^103,104^

By feeding into the TCA cycle, glutamine supports ATP generation via OXPHOS, which is particularly important in cancers such as African American bladder cancer, where mitochondrial complex I activation drives a glutamine-fueled OXPHOS phenotype linked to disease progression.^105,106^ The nitrogen from glutamine is essential for the synthesis of nucleotides and NEAAs such as asparagine, proline, and glycine, which are required for protein and collagen synthesis in cancer-associated fibroblasts (CAFs) and myofibroblasts.^107,108^ As a contributor to the synthesis of GSH, glutamine metabolism is also pivotal for maintaining redox homeostasis in cancer cells.^79^ In cervical cancers with PI3K pathway mutations, inhibition of GLS induces thiol-mediated oxidative stress and radiosensitization.^109^ Similarly, in pancreatic cancer, oncogenic KRAS upregulates NRF2, which rewires metabolism to enhance glutamine utilization and bolsters the antioxidant network, contributing to chemoresistance.^110^ Furthermore, glutamine-derived metabolites can act as signaling molecules. In renal cell carcinoma and other cancers, glutamine can be converted to the oncometabolite 2-hydroxyglutarate, which is associated with disease progression in plasma cell malignancies.^111^ In breast and other cancers, aberrant expression of glutamate decarboxylase allows tumor cells to synthesize the neurotransmitter γ-aminobutyric acid (GABA) from glutamine, which then activates autocrine signaling to promote proliferation and suppress CD8+ T-cell infiltration, creating an immunosuppressive microenvironment.^112^

### Glutamine metabolism in the tumor microenvironment

The metabolic coupling between cancer cells and CAFs represents a cornerstone of glutamine metabolism within the tumor microenvironment (TME). In PDAC, pancreatic stellate cells (PSCs, a major source of CAFs) exhibit upregulated GS via the Wnt/β-catenin/TCF7 pathway, secreting glutamine to fuel the growth and oxidative metabolism of adjacent cancer cells.^108^ In TNBC, estrogen-activated G protein-coupled estrogen receptor (GPER) in CAFs enhances GS and lactate dehydrogenase B (LDHB) expression, leading to increased glutamine production and secretion, which is subsequently taken up by TNBC cells via SLC1A5 to promote mitochondrial activity and tumor progression.^113^ Similarly, epigenetic silencing of the Ras inhibitor RASAL3 in prostatic CAFs drives macropinocytosis-mediated glutamine synthesis, supporting castration-resistant prostate cancer growth and neuroendocrine differentiation.^114^ These findings collectively establish CAFs as critical glutamine suppliers, with their targeting—such as using ultrasound-responsive nanodroplets to deliver SLC1A5 inhibitors and GS-targeting siRNA specifically to CAFs—disrupting this metabolic symbiosis and inhibiting tumor growth.^115^ This metabolic interplay creates a nutrient-depleted TME, setting the stage for intense competition between various cellular constituents.

The limited availability of glutamine in the TME fosters a state of metabolic competition that profoundly influences antitumor immunity, often to the detriment of effector immune cells such as tumor-associated macrophages and myeloid-derived suppressor cells (MDSCs), which compete for glutamine and impair nutrient access to cytotoxic T cells (Fig. 2b).^116–119^ Tumor cells, with their high anabolic demands and upregulated glutamine transporters such as SLC1A5 and SLC7A5, often outcompete tumor-infiltrating lymphocytes for this critical nutrient.^120^ This “glutamine steal” scenario impairs CD8+ T-cell activation and effector function, as evidenced by an inverse correlation between glutamine metabolic gene signatures and T-cell cytotoxicity in basal-like breast cancer. The consequences of this competition are multifaceted. In clear cell renal cell carcinoma, tumor cell glutamine consumption creates local deprivation, inducing interleukin-23 (IL-23) secretion by tumor-associated macrophages via HIF-1α activation, which subsequently promotes regulatory T-cell (Treg) proliferation and immunosuppression.^121,122^ In pancreatic cancer, single-cell analyses have revealed that tumor cells with high glutamine metabolism scores skew CD8+ T-cell differentiation toward less cytotoxic or more exhausted states.^123^ In fact, sufficient glutamine can reduce the formation of neutrophil extracellular traps (NETs) that inhibit the spatial distribution of CD8+ T cells within the tumor, thereby establishing cytotoxic CD8+ T-cell hubs in human cancer.^124,125^ Glutamine enhances the ability of conventional type 1 dendritic cells (cDC1s) to deliver antigens to CD8+ T cells.^126^ Glutamine deprivation enhances the differentiation of naïve T-cells into Treg cells rather than effector T cells.^127^ In cancer cells, glutamine‑derived metabolites such as adenosine can inhibit T-cell activation and facilitate immune escape (Fig. 2b).^128–130^ Extracellular adenosine inhibits T-cell activation and proliferation through a variety of different pathways, such as binding to the A2A receptor, which activates the typical G protein and triggers the cAMP/PKA/CREB pathway.^131–133^ Cancer cells also suppress immune cell development and activation by secreting extracellular signaling factors, including mitochondrial-derived vesicles and other extracellular vesicles, which promote the reorganization of the microenvironment.^134^ Furthermore, glutamine shortage in the liver cancer TME drives the infiltration of immunosuppressive GPR109A+ MDSCs and M2-like tumor-associated macrophages, a process dependent on an endoplasmic reticulum stress response triggered by competitive glutamine uptake.^135^ Even innate-like immune cells such as γδ T cells in HCC exhibit functional exhaustion and upregulated glutamine metabolism in the nutrient-poor TME.^136^ Thus, tumor cell-intrinsic glutamine addiction actively shapes an immunosuppressive landscape by restricting nutrient availability and initiating suppressive signaling cascades. The mechanisms of crosstalk between cancer cells and immune cells are complex, but glutamine metabolism may play a critical role.

Most studies support that inhibition of glutamine metabolism increases the killing activity of T cells against tumor cells,^120^ and a few studies found that cytotoxic T cells were also damaged.^137,138^ One possible explanation is that although glutamine transporters are inhibited by inhibitors in all cells in the TME, tumor cells are more sensitive to inhibitors because of their greater number of transporters. Additionally, once glutamine transporters and glutaminolysis enzymes are inhibited, the availability of glutamine in the microenvironment will be increased, which is beneficial to the acquisition of nutrients for tumoricidal cells. This may partly explain why glutamine blockade tends to inhibit only cancer cells but instead has a supportive effect on immune cells that also require glutamine.^116^

Beyond serving as a biosynthetic fuel, glutamine metabolism directly modulates the functional polarization and activity of diverse immune cells within the TME, further entrenching immunosuppression. Glutaminolysis is crucial for the generation and suppressive capacity of immature myeloid cells (IMCs) and MDSCs; inhibition of glutaminase 1 (GLS1) reduces these populations and enhances anti-PD-L1 therapy efficacy.^139^ Tumor-derived extracellular vesicles carrying GLS1 can drive M2 macrophage polarization, contributing to trastuzumab resistance in HER2-positive gastric cancer.^140^ Conversely, in ovarian cancer, glutamine-addicted cancer cells release N-acetylaspartate (NAA), which enforces GS expression in macrophages, pushing them toward an M2-like, protumoral phenotype.^141^ For T cells, the impact is dichotomous. While effector T-cell differentiation requires glutamine, its deprivation or pharmacological inhibition can paradoxically be beneficial by preserving less differentiated, memory-like phenotypes with superior persistence, a principle applied to enhance chimeric antigen receptor (CAR)-T-cell therapy.^142^ However, within the TME, chronic glutamine blockade can upregulate PD-L1 on cancer cells via mechanisms involving ROS accumulation and EGFR/ERK signaling, leading to T-cell inhibition—a resistance mechanism that can be reversed by combining glutamine inhibitors with agents such as gefitinib.^143,144^ However, up-regulation of PD-L1 can increase the efficacy of immunotherapy, so combination therapy is optimization.^138^ Furthermore, metabolic byproducts such as ammonia, generated from glutamine catabolism in T cells, can induce ammonia death, potentially limiting sustained antitumor immune responses.^30,145^ This intricate crosstalk positions glutamine metabolic pathways as central regulators of immune cell fate and function.

Angiogenesis, which is essential for tumor growth and metastasis, is another process coopted by glutamine metabolism within the TME. Endothelial cells (ECs) rely on glutamine to drive vessel sprouting. Glutamine functions not mainly as an energy substrate, but to support macromolecular biosynthesis, and cellular redox homeostasis.^146^ A crucial link is established through asparagine synthesis; glutamine-derived nitrogen is essential for producing asparagine via ASNS, and silencing ASNS impairs EC sprouting even in the presence of extracellular glutamine and asparagine.^146^ This metabolic dependency makes the tumor vasculature vulnerable to glutamine-targeting strategies. In TNBC, nanoplatforms that combine glutamine transport inhibition (using V9302) with angiogenesis disruption (using the vascular disrupting agent CA4P) achieve enhanced tumor suppression by simultaneously blocking the primary nutrient pathway and compensatory nutrient delivery via new vessels.^147,148^ Furthermore, metabolic reprogramming in other TME components indirectly supports angiogenesis. For instance, in HER2-positive gastric cancer, glutamine metabolism-driven M2 macrophage polarization promotes proangiogenic functions, contributing to a therapy-resistant niche.^140^ The sympathetic nervous system, which infiltrates the TME, also influences angiogenesis by enhancing glycolysis in ECs, a process intertwined with broader metabolic regulation that includes glutamine metabolism.^149^ Therefore, targeting glutamine metabolism offers a multipronged approach to impair the tumor’s nutrient supply network by directly affecting EC biology and indirectly modulating proangiogenic signals from stromal and immune cells.

### Glutamine as a therapeutic target

This heightened dependence on glutamine renders the enzymatic pathways of glutaminolysis and its membrane transporters compelling targets for therapeutic intervention.^21,78,83^ The clinical realization of this concept is driven by agents designed to inhibit glutamine catabolism and block membrane transport (Fig. 2c).^150^

The most clinically advanced strategy involves direct inhibition of GLS, with telaglenastat (CB-839) being a first-in-class, orally bioavailable allosteric inhibitor.^151^ CB-839 is a noncompetitive inhibitor of GLS1 with an IC50 of 23 nM. Preclinical studies across diverse malignancies have validated its antitumor potential. In soft tissue sarcoma models, CB-839 successfully inhibited tumor growth, demonstrating therapeutic efficacy in autochthonous settings.^152^ In acute myeloid leukemia (AML), CB-839 disrupts the TCA cycle and redox balance, and its combination with agents such as arsenic trioxide exacerbates mitochondrial ROS to induce cancer apoptosis.^153^ Hyperpolarized magnetic resonance imaging using pyruvate has been proposed as a noninvasive method to monitor the metabolic effects of CB-839 in AML.^154^ Beyond monotherapy, GLS inhibition sensitizes tumors to conventional therapies. It reverses chemoresistance in KRAS-driven pancreatic cancer and anaplastic thyroid cancer by undermining antioxidant defenses and one-carbon metabolism, respectively.^110,155^ In HCC, CB-839 enhances the efficacy of 5-fluorouracil in resistant cells by promoting ferroptosis.^156^ However, induction of ASNS in response to CB-839 reprograms glutamine metabolism to sustain redox homeostasis and mTOR signaling, thereby conferring resistance.^157^ Similarly, loss of glutamic-oxaloacetic transaminase 2 (GOT2) in HCC reprograms glutamine metabolism to fuel progression but, paradoxically, creates a vulnerability to GLS inhibitors.^158^ The clinical development of CB-839 has included phase I/II trials, establishing its safety profile and a recommended phase II dose, with signals of activity in renal cell carcinoma.^159^ However, a phase III trial combining CB-839 with cabozantinib in advanced renal cell carcinoma did not meet its primary endpoint, highlighting the complexity of achieving clinical efficacy.^160^ Other GLS inhibitors, including BPTES and DON, are also under investigation in cancer.^151,161^ BPTES is a noncompetitive inhibitor of GLS1 with a Ki of 3 μM and is less potent than CB-839 but with faster inhibition kinetics. DON is a glutamine antagonist that binds covalently to the enzyme active site of different glutamine-using enzymes, including GLS and glutamine amidotransferases. Nonselective inhibitor of glutamine metabolism. Epigallocatechin gallate and R162 can inhibit the activity of GDH and result in imbalanced redox homeostasis, leading to cancer cell death.^162,163^ Recent studies have found that inhibition of GS activity by l-methionine sulfoximine (MSO) can enhance the radiosensitivity of HCC.^164^ Similar to MSO, another nonprotein amino acid, dl-phosphinothricin (PPT), which inhibits glutamine synthetase, can also activate the mTORC1 pathway, further depleting glutamine.^165^ Bisphosphonates can also inhibit GS in *Mycobacterium tuberculosis* to control tuberculosis, but their role in inhibiting glutamine synthesis in the treatment of cancer remains to be studied.^166^

Parallel to targeting GLS, inhibition of glutamine transporters, particularly the primary transporter SLC1A5, represents a complementary approach. Notably, although highly expressed in cancer cells, glutamine transporters are also widely expressed in other proliferating cells, including immune cells. γ-L-Glutamyl-p-nitroanilide (GPNA), although it is known that it also inhibits other sodium-dependent amino acid transporters and the mTORC1 pathway, blocks SLC1A5 function and has shown efficacy in preclinical models.^167^ V9302, a new generation of more potent SLC1A5 inhibitors, is currently the preferred agent. In pancreatic cancer, V9302, when codelivered with a GLUT1 inhibitor in nanoparticles, induces dual starvation leading to pyroptosis.^168^ In breast cancer, V9302 inhibits glutamine uptake, synergizing with photodynamic therapy to induce ferroptosis by blocking GSH synthesis and activating lipophagy.^169^ This strategy also applies to hematological malignancies; V9302 synergizes with proteasome inhibitors in plasma cell myeloma by inducing a catastrophic unfolded protein response and oxidative stress.^170^ V9302 attenuates TME-induced resistance to proapoptotic drugs, such as venetoclax, in chronic lymphocytic leukemia.^171^ V9302 also inhibited SLC25A22. In renal cell carcinoma models, V9302 suppresses tumor growth and enhances the anti-PD-1 immunotherapy response by remodeling the immunosuppressive microenvironment.^120,147^ Another SLC1A5 inhibitor, IMD-0354, identified through a screen for compounds that prevent its plasma membrane localization, potently inhibits glutamine uptake, suppresses mTOR signaling, and induces cell death in melanoma models.^172^ An SLC1A5_var inhibitor called iMQT_020 has recently been developed that reduces glutamine anaplerosis and OXPHOS by inhibiting glutamine entry into mitochondria.^173^ Beyond SLC1A5, other amino acid transporters involved in glutamine metabolism are emerging as targets. In TNBC, SLC7A5 blockade by JPH203 enhances anti-PD-1 therapy by increasing CD8+ T-cell infiltration.^174^ Functional inhibition of SNAT1 with α-(methylamino) isobutyric acid (MeAIB) could reduce cancer cell growth.^175^ These findings underscore the therapeutic potential of disrupting glutamine at the point of cellular entry.

Cells that are glutamine-deprived by blockade of the transporter can maintain proliferation by intracellular synthesis of glutamine. Asparaginase has GLS activity and can block the ability of cancer cells to adapt to glutamine deprivation.^176,177^ Enzymatic depletion of circulating glutamine using engineered L-asparaginase variants such as JZP-458 shows promise in glutamine-addicted cancers.^178^ These agents reduce plasma glutamine levels, starving tumors while sparing normal cells. Phase II trials in ALL demonstrate efficacy with reduced hypersensitivity compared to native asparaginase.^179^ Mechanistically, highly expressed GLS1 aggregates into micron-scale rod-like structures under glutamine starvation that could lead to apoptosis.^180^ In glutamine depletion, the glutamine-dependent synthesis of asparagine is also inhibited, which leads to the limitation of mitochondrial genome-encoded protein synthesis and the destruction of mitochondrial respiratory chain complexes, which eventually leads to a large accumulation of ROS and apoptosis.

Given the metabolic plasticity of tumors, monotherapy with glutamine pathway inhibitors frequently triggers adaptive resistance mechanisms, necessitating rational combination strategies.^21,44,181^ One major axis combines glutamine inhibition with conventional cancer therapies to overcome resistance. As noted, GLS inhibitors radiosensitize multiple cancers, including prostate, cervical, and sarcoma.^102,109,182,183^ Combining GLS inhibitors with chemotherapy, such as gemcitabine in pancreatic cancer or 5-FU in HCC, has proven effective in preclinical models by exacerbating oxidative stress.^110,156^ A second critical strategy addresses the immune-modulatory consequences of glutamine inhibition. While initially promoting antitumor immunity, prolonged glutamine blockade can upregulate PD-L1 on cancer cells via mechanisms such as ROS/EGFR/ERK signaling, leading to immunosuppression.^144^ This has spurred the development of combination therapies and novel agents that simultaneously target metabolism and immune checkpoints. Nanoplatforms codelivering CB-839 or V9302 with PD-L1/PD-1 blockers have shown superior efficacy in remodeling the tumor immune microenvironment.^184–186^ Prodrugs such as JHU083 (a DON prodrug) and HB023 (a conjugate of a glutamine antagonist and the PD-L1 inhibitor JQ1) are designed to mitigate toxicity and counteract PD-L1 upregulation, thereby enhancing T-cell function.^144,187^

Dual metabolic inhibition, targeting both glycolysis and glutaminolysis, is a powerful approach to counteract compensatory pathways. Cancer cells deprived of glucose often increase glutamine utilization, and vice versa.^188,189^ Other nanocarriers codeliver glycolysis inhibitors (lonidamine, glucose oxidase) with GLS inhibitors (BPTES) to achieve synergistic tumor starvation.^189–191^ This concept extends to targeting glutamine metabolism alongside other metabolic vulnerabilities, such as combining GLS inhibitors with LDHA or MCT1 inhibition in neuroblastoma or with one-carbon metabolism inhibition in anaplastic thyroid cancer.^155,192,193^ In pancreatic cancer, combining the glutamine antagonist DON (or its prodrug DRP-104^194^) with MEK/ERK pathway inhibition addresses compensatory survival signaling and significantly improves outcomes.^195^ The natural compound Osmundacetone exerts antitumor effects in NSCLC by inhibiting the glutamine/glutamate/α-KG axis and OXPHOS.^196^

The clinical translation of glutamine metabolism inhibitors faces challenges, including patient stratification, on-target toxicity, and adaptive resistance. Metabolic imaging using PET tracers such as 11C-glutamine offers a noninvasive way to visualize glutamine-avid tumors and potentially monitor response.^197^ Molecular biomarkers such as PI3K pathway mutations, low GOT2 expression, or a B-cell activation gene signature (“B-score”) have been associated with sensitivity to GLS inhibition in specific contexts.^109,158,198^ Furthermore, natural compounds and traditional medicine formulations are being explored for their ability to modulate glutamine metabolism. Ginsenoside CK induces apoptosis in TNBC by downregulating GLS1.^199^ Jianpi Jiedu formula inhibits colorectal cancer liver metastasis by modulating glutamine metabolism via the YTHDF1/GID8 axis.^200^ The bile acid lithocholic acid suppresses gallbladder cancer by downregulating GLS and inducing ferroptosis.^201^ These diverse approaches highlight the breadth of ongoing research aimed at refining glutamine-targeted therapies through improved delivery systems, such as tumor-targeted nanoparticles and prodrugs, and intelligent combination regimens informed by a deep understanding of tumor-specific metabolic dependencies and immune context.^21,96,145^

## Immunomodulation by glutamine

### Modulation of glutamine metabolism in innate immune cells

**Fig. 3:**
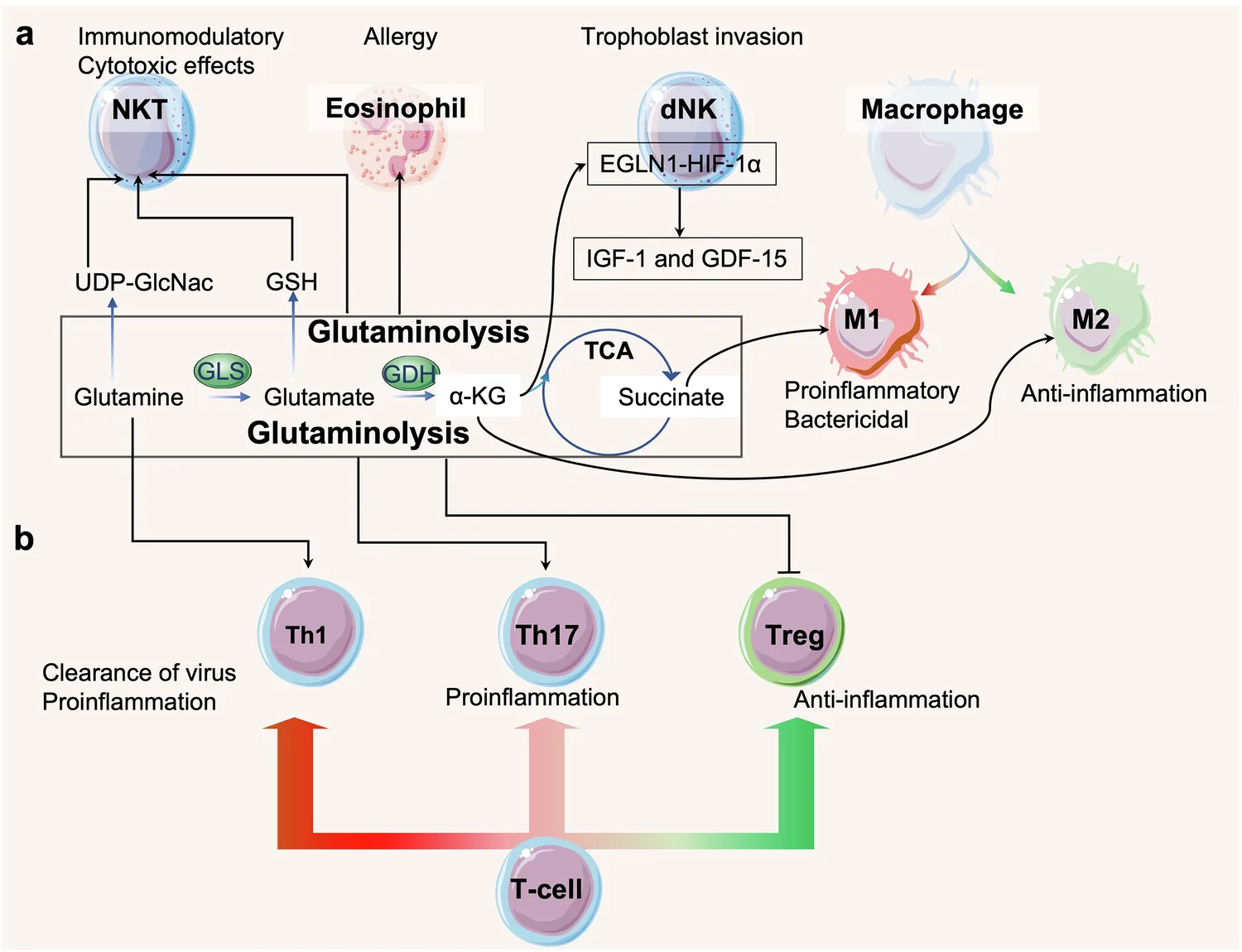
Glutamine metabolism and immunity. **a** Glutamine metabolism and innate immune cells. Glutamine promotes the function of NKT cells through glutaminolysis, GSH and HBP pathways. Glutaminolysis contributes to eosinophil expansion, activation, and infiltration. dNK depend on α-KG to promote the uterine implantation of trophoblast cells. Glutamine has an important effect on the polarization of macrophages, that α-KG promotes polarization to M1, and succinate promotes to M2. **b** Glutaminolysis is important for T cell differentiation. Glutamine is necessary for Th1 cell function. Inhibition of glutaminolysis could promote the differentiation of T-cell into Tregs. NKT natural killer T-cell, GSH glutathione, HBP Hexosamine Biosynthetic Pathway, dNK decidual NK, Th help T-cell, Treg regulatory T-cell

Glutamine orchestrates the function of innate immune cells as well (Fig. 3a). The metabolic plasticity of macrophages is profoundly influenced by glutamine availability. The metabolic fate of glutamine is decisive: glutaminolysis yielding α-KG promotes M2 polarization, while glutamine-dependent anaplerosis leading to succinate accumulation favors the M1 phenotype.^202,203^ This balance is crucial in metabolic disease, as evidenced by altered serum levels of glutamine, α-KG, and succinate in patients with obesity or diabetes.^202^ The context dependency of the effect of glutamine is further highlighted in neonatal gut injury, where prophylactic glutamine or glutamate administration suppresses inflammatory macrophage responses by restoring GDH activity and α-KG production, whereas supplementation after disease onset exacerbates inflammation by favoring succinate metabolism.^204^

Additionally, glutamine metabolism is a key interface in host‒interactions and cancer immunology. In tuberculosis, pharmacological inhibition of glutamine metabolism with JHU083 exerts dual antibacterial and host-directed immunomodulatory activities. It enhances the antibacterial function of macrophages by promoting nitric oxide production and reprograms the lung myeloid compartment, increasing proinflammatory cell infiltration while reducing immunosuppressive populations, leading to improved bacterial control and survival.^205^ Conversely, intracellular pathogens such as *Mycobacterium abscessus* can rewire infected macrophages to increase OXPHOS, and intriguingly, restricting mitochondrial oxidation of glutamine or fatty acids enhances bacterial growth, indicating that host glutamine oxidation is part of a protective antimicrobial response.^206^

As resident macrophages of the central nervous system (CNS), microglia are equally governed by glutamine metabolic pathways, particularly in the context of neurodegeneration and injury. GS has emerged as a critical regulator of microglial inflammatory responses. Inhibition of GS in microglia exacerbates the release of inflammatory mediators upon LPS activation, perturbs redox balance, decreases insulin-mediated glucose uptake, and increases neuronal death in coculture systems.^207^ In vivo, microglia-specific GS ablation amplifies neuroinflammation, and reduced GS expression correlates with experimental autoimmune encephalomyelitis.^207^ Following traumatic brain injury (TBI), acute proinflammatory microglial activation is associated with disrupted mitochondrial metabolism. The IRG1/itaconate pathway, a key immunometabolic axis, is implicated in recovery; supplementation with 4-octyl itaconate restores oxidative metabolism of glucose, glutamine, and fatty acids, attenuates inflammation, and improves long-term outcomes, indicating that supporting microglial bioenergetics through related metabolic pathways is therapeutic.^208^

The influence of glutamine extends to other innate immune cells, including eosinophils and innate-like lymphocytes, in diverse pathological settings. In allergic asthma, glutamine metabolism is extensively reprogrammed during eosinophil expansion and activation. Pharmacological inhibition of GLS1 with CB839 potently attenuates eosinophil expansion in vitro and reduces eosinophil infiltration, tissue pathology, and inflammation in vivo, identifying GLS1 as a promising target for eosinophilic asthma.^209^ Natural killer T (NKT) cells exhibit a distinct glutamine-addicted phenotype compared to conventional CD4+ T cells. Upon activation, they increase glutaminolysis to fuel the TCA cycle, GSH synthesis, and the HBP, which are all critical for their homeostasis, mitochondrial function, and cytokine production (IL-4 and IFNγ).^210^ In pregnancy, decidual NK (dNK) cells utilize glutaminolysis to generate α-KG, which downregulates the EGLN1-HIF-1α axis, promoting the secretion of factors such as IGF-1 and GDF-15 that support trophoblast invasion. Impairing this glutamine/α-KG metabolism in dNK cells leads to pregnancy loss in mice.^211^ Furthermore, in endometriosis, decreased hematopoietic cellular kinase (HCK) in peritoneal macrophages dysregulates glutamine metabolism and increases autophagy, leading to impaired phagocytic clearance of ectopic lesions; this defect can be rescued by glutamine-pretreated macrophages.^212^

Therapeutic modulation of glutamine availability or metabolism in innate immune cells presents a viable strategy across diseases. In cerebral malaria, administration of DON at late stages rapidly restores blood‒brain barrier (BBB) integrity, reduces brain swelling, and dampens pathogenic CD8+ T-cell function, rescuing mice from fatal disease.^213^ In liver ischemia/reperfusion injury, depletion of the gut microbiota reduces disease severity by increasing the availability of glutamine-derived α-KG in the portal circulation, which in turn promotes M2 macrophage polarization via enhanced OXPHOS.^214^ These findings collectively underscore that glutamine metabolism is a master regulator of innate immune cell fate and function. Its manipulation—through deprivation, supplementation, or enzymatic inhibition—holds significant potential for recalibrating immune responses in favor of tissue protection, pathogen clearance, or antitumor immunity, depending on the specific pathological and metabolic context.

### Fueling adaptive immunity

Glutamine metabolism is equally indispensable for the adaptive immune system, where it serves as a critical bioenergetic and biosynthetic substrate for T lymphocytes (Fig. 3b). Upon activation through the T-cell receptor (TCR) and costimulatory signals such as CD28, T cells undergo profound metabolic reprogramming to meet the demands of rapid clonal expansion, cytokine production, and effector function.^17,215^ This reprogramming entails a sharp increase not only in glycolysis but also in glutamine consumption, which rivals or exceeds that of glucose in rapidly dividing T cells.^215^ Glutamine is catabolized through glutaminolysis to generate ATP and provide nitrogen for nucleotide and amino acid synthesis. Disruption of this metabolic switch impairs T-cell activation and proliferation. For instance, in patients with CARMIL2 deficiency—an inborn error of immunity characterized by T-cell dysfunction—activation-induced metabolic reprogramming is severely disrupted, with transcriptomic and metabolomic profiling revealing deficits in mTOR signaling and glutamine metabolism.^216^ Notably, ex vivo glutamine supplementation can partially restore signaling and cytokine production in these patient-derived T cells, highlighting a direct therapeutic avenue targeting metabolism to correct immune dysfunction.^216^ The expression of glutamine transporters is upregulated upon T-cell activation, and their function is nonredundant for proper immune responses.^217–219^

The fate of activated CD4+ T helper (Th) cells into distinct functional subsets is intricately guided by metabolic cues, with glutamine metabolism playing a decisive role in steering differentiation toward inflammatory or regulatory phenotypes. Glutaminolysis is particularly crucial for the differentiation of Th1 and Th17 cells, which are drivers of many autoimmune and inflammatory conditions. In oral lichen planus, an autoimmune disease, SLC1A5-mediated glutamine uptake in T cells promotes Th1 differentiation through a ROS-EGR1-PAC1 signaling axis that modulates STAT3 phosphorylation.^218^ Similarly, during influenza infection, CD4+ T-cell-intrinsic arginase 1 (Arg1) acts as an unexpected rheostat for the Th1 response lifecycle; its loss perturbs glutamine metabolism, accelerating Th1 effector kinetics and improving viral clearance with reduced lung pathology.^220^ The balance between proinflammatory Th17 cells and anti-inflammatory Tregs is also metabolically sensitive. Glutamine metabolism supports Th17 differentiation, and its inhibition can tilt the balance toward Tregs.^221–223^ For example, the natural compound celastrol, used traditionally for autoimmune diseases, attenuates Th17 differentiation by downregulating GLS1 expression through an HDAC3/FOXO3-dependent pathway, thereby inhibiting glutaminolysis.^224^ This principle is demonstrated therapeutically in autoimmune hepatitis, where pharmacological blockade of glutamine metabolism using the GLS antagonist DON or its prodrug JHU083 suppresses the activation and differentiation of pathogenic Th1 and Th17 cells, ameliorating disease.^219^ The mechanistic underpinning involves the downregulation of the amino acid transporter SLC7A5 and the subsequent dampening of mTOR signaling, a central integrator of metabolic and immune signals.^219^

Within the TME, a fierce metabolic competition for nutrients such as glutamine occurs between rapidly proliferating cancer cells and tumor-infiltrating lymphocytes. However, the relationship is complex. In CRC^225^ and cervical squamous cell carcinoma,^226^ targeting the mitochondrial glutamate transporter SLC25A22 enhances CD8+ T-cell cytotoxicity and sensitizes tumors to anti-PD-1 therapy. However, glutamine deprivation can also promote autophagy-dependent degradation of the interferon-gamma receptor (IFNGR1) in cancer cells, rendering them resistant to IFN-γ signaling and anti-PD-1 therapy; V9302 counterintuitively enhances immunotherapy efficacy in this context by impairing lysosomal activity and stabilizing IFNGR1, independent of its effect on glutamine deprivation.^227^ These findings underscore that the timing, selectivity, and combination of glutamine metabolic interventions are critical for achieving immunostimulatory outcomes.

Beyond autoimmunity and cancer, the principles of glutamine-dependent T-cell regulation inform approaches in transplantation and tolerance. Alloreactive T cells driving graft-versus-host disease (GVHD) utilize glutamine metabolism alongside glycolysis and fatty acid oxidation.^17^ The fundamental link between T-cell metabolism and function suggests that modulating glutamine pathways could influence alloreactive responses. Furthermore, the stability and function of Tregs, which are essential for inducing transplantation tolerance,^228^ are influenced by metabolic pathways. Although the provided literature does not detail glutamine’s role in Tregs specifically,^229^ the broader context of immunometabolism^222^ and the critical balance between effector and regulatory subsets^221^ imply that metabolic manipulation could be leveraged to promote Treg-mediated tolerance, representing a promising area for future investigation alongside cellular therapy approaches.^228,230^ Collectively, the evidence solidifies glutamine metabolism as a central integrator of T-cell fate, whose targeted modulation holds immense potential to enhance antitumor immunity, suppress pathological autoimmunity, and optimize next-generation adoptive cell therapies.

### Clinical applications in immunonutrition and cell-based therapies

Building on the fundamental principles of glutamine-dependent immune cell regulation, translating these insights into clinical practice manifests primarily through two interconnected strategies: the systemic administration of glutamine as an immunonutrient and the metabolic engineering of therapeutic immune cells. Immunonutrition, defined as the provision of specific nutrients above dietary levels to modulate immune function and inflammation,^231^ has been extensively investigated, with glutamine being a cornerstone component.^5,232^ In surgical oncology, perioperative glutamine supplementation is associated with improved clinical outcomes.

In mixed medical-surgical critically ill populations, the evidence is contradictory. Some trials and meta-analyses in specific subgroups, such as severe acute pancreatitis (SAP), suggest benefits. A meta-analysis of 30 RCTs in SAP found that glutamine supplementation was associated with lower mortality, shorter hospital stays, fewer complications, and improved immune and organ function markers.^233^ Another meta-analysis focusing on parenteral immunonutrition (including glutamine and omega-3 fatty acids) in acute pancreatitis also reported reductions in infectious complications, mortality, and hospital stay.^234^ Conversely, other large trials in heterogeneous ICU populations have raised safety concerns.^235^ In chronic inflammatory and immune-mediated conditions, glutamine supplementation shows variable efficacy, often related to its role in gut barrier function and mucosal immunity.^236^ However, experimental and smaller clinical studies suggest potential mechanisms of benefit. An experimental study in rats found that a combination of arginine, glutamine, and β-hydroxy β-methylbutyrate (HMB) produced coordinated anti-inflammatory and metabolic benefits.^237^ Furthermore, a case‒control study in COVID-19 patients with respiratory infection reported that short-term glutamine supplementation reduced serum levels of inflammatory cytokines (IL-1β, TNF-α, hs-CRP) and increased appetite,^238^ aligning with hypotheses that glutamine deficiency may predispose patients to severe COVID-19.^239^

## Glutamine in metabolic and cardiovascular diseases

### Links to insulin signaling, glucose homeostasis, and diabetic complications

**Fig. 4:**
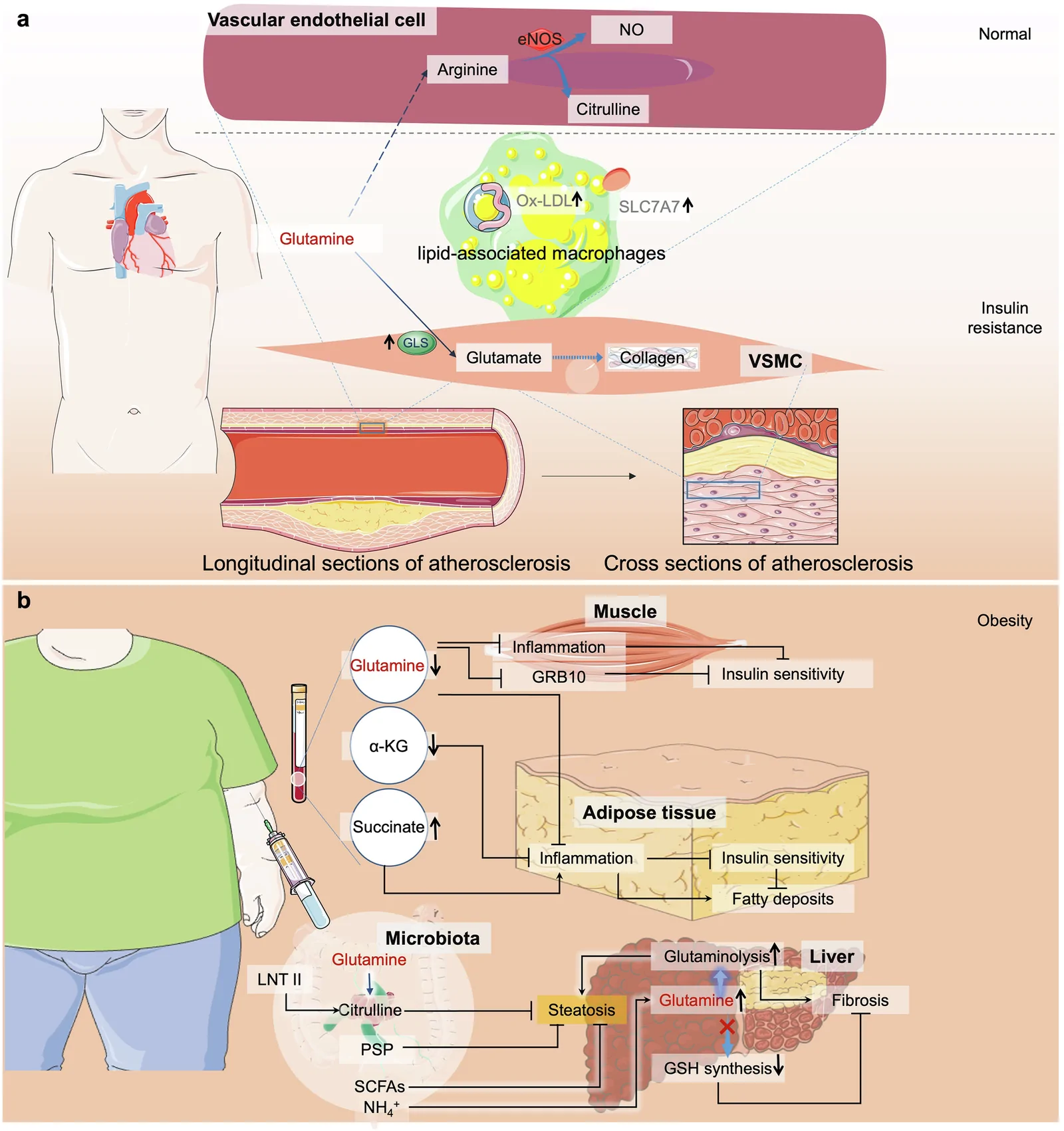
Glutamine metabolism and chronic metabolic diseases. **a** Dual role of glutamine metabolism in cardiovascular disease. Under normal conditions, vascular endothelial cells sustain functional citrulline–arginine–eNOS signaling to preserve endothelial integrity. Upon IR, Ox-LDL triggers dysfunction in endothelial cells, lipid-associated macrophages and VSMCs, accompanied by upregulated SLC7A7. Impaired arginine/eNOS signaling and hyperglutaminosis promote excessive collagen accumulation, vascular wall remodeling, and the formation of atherosclerotic plaques. **b** The serum levels of glutamine and akg are decreased and succinate levels are increased in obese individuals, which together play a role in promoting a long-term chronic inflammatory state and driving IR in muscle and adipose tissue. Citrulline produced by gut microbiota metabolizing glutamine can inhibit hepatic steatosis, and LNT II could promote citrulline synthesis. SCFAs and ammonia produced by microbiota affect hepatic glutamine metabolism. eNOS endothelial nitric oxide synthase, Ox-LDL oxidized low-density lipoprotein, NO nitric oxide, VSMC vascular smooth muscle cell, IR insulin resistance, LNT II Lacto-N-triose II, SCFAs short-chain fatty acid

In diabetes mellitus (DM), abnormal glutamine metabolism is closely related to islet β-cell dysfunction, insulin resistance (IR) and chronic inflammation. Glutamine is not a stimulator of insulin secretion but has an amplifying effect on insulin secretion in β-cells, and deprivation leads to insulin secretory dysfunction.^240–243^ Glutamine may influence systemic insulin sensitivity and the pathogenesis of diabetic complications, positioning it as a significant biomarker and a potential therapeutic node within the broader landscape of cardiometabolic disease (Fig. 4a).^244,245^ Plasma metabolomic profiling in individuals at high risk for T2DM has revealed that the glutamine/glutamate ratio is associated with incident diabetes, although this association may be attenuated upon adjustment for clinical covariates.^246^ More specifically, circulating glutamine levels correlate inversely with BMI and the homeostatic model assessment of IR (HOMA-IR) in men, suggesting a direct link between glutamine availability and systemic insulin sensitivity.^56^ This relationship is further underscored by genetic evidence; the diabetes-associated gene *TCF7L2*, the strongest common genetic risk factor for T2DM, is crucial for maintaining hepatic glutamine homeostasis.^242,247^

The molecular mechanisms through which glutamine influences insulin signaling and glucose metabolism are multifaceted. In skeletal muscle, a primary site of insulin-mediated glucose disposal, glutamine levels directly regulate the inflammatory state. Experimental reduction of glutamine in human myotubes and in diet-induced obese mice exacerbates the expression of inflammatory markers, while systemic glutamine supplementation in obese mice reduces skeletal muscle inflammation, downregulates the insulin signaling inhibitor GRB10, and ultimately improves whole-body insulin sensitivity and glucose homeostasis.^56^ In adipose tissue, chronic oral glutamine supplementation in diet-induced obese rats induces a paradoxical, tissue-specific IR via the HBP. This localized effect in adipocytes is associated with reduced adipose mass and a concomitant improvement in systemic insulin action, hepatic glucose production, and muscle glucose uptake, suggesting that glutamine may reprogram fuel partitioning to improve metabolic flexibility.^248^ Furthermore, glutamine treatment enhanced postprandial insulin response, stimulated an increase in plasma glucagon-like peptide (GLP)-1 concentrations, and limited the extent of postprandial glycemia.^249^ Mechanistically, glutamine exerts its secretagogue effect on GLP-1 by increasing the cytosolic Ca²⁺ concentration and cAMP in gut L cells.^249^

The dysregulation of glutamine metabolism actively contributes to the development of diabetic complications, particularly cardiovascular disease (CVD) and diabetic kidney disease (DKD). A genetic link has been established between a coronary heart disease (CHD) risk locus (rs10911021) specific to T2DM and glutamine synthesis (Fig. 4a). The risk allele is associated with lower expression of GS in endothelial cells, leading to impairments in the γ-glutamyl cycle, reduced GSH-to-glutamate ratio, and impaired detoxification of the atherogenic compound methylglyoxal. Notably, glutamine supplementation in vitro completely prevented the methylglyoxal accumulation caused by GS downregulation, suggesting a targeted nutritional strategy for at-risk individuals.^250^

Therapeutic modulation of glutamine availability or its metabolic pathways presents a promising, although complex, strategy for improving metabolic health. Clinical and preclinical interventions demonstrate potential benefits. Oral glutamine supplementation in overweight and obese humans reduces waist circumference and serum inflammatory markers such as lipopolysaccharide.^248^ Intravenous glutamine administration following bariatric surgery in obese mice improves postoperative glucose tolerance and attenuates adipose tissue inflammation.^251^ Probiotic and synbiotic approaches that elevate circulating glutamine levels show efficacy; treatment with *Lactiplantibacillus plantarum* 84-3, which elevates serum L-glutamine, improves glucose homeostasis in diabetic rats by activating the hepatic AMPK/PPARγ pathway.^252^ Similarly, a synbiotic combination of *Bacillus subtilis* and L-alanyl-L-glutamine significantly improved fasting glucose, postprandial glycemic response, and HbA1c in a real-life study of prediabetic subjects.^253^ Furthermore, the metabolic effects of commonly prescribed drugs intersect with glutamine pathways. The antidiabetic drug metformin, beyond its glucocentric actions, modulates amino acid homeostasis by suppressing cellular amino acid uptake, leading to a selective accumulation of branched-chain amino acids and glutamine in plasma, which may contribute to its therapeutic mechanisms.^254^ Conversely, the SGLT2 inhibitor class, used for T2DM and heart failure, can have off-target effects on glutamine metabolism. Canagliflozin, uniquely among gliflozins, inhibits mitochondrial GDH and complex I at clinically relevant concentrations, disrupting glutamine anaplerosis and posing a potential risk for cytotoxicity in proliferating renal tubular cells, which may explain its associated acute kidney injury risk.^255^ Nutritional interventions targeting wound healing in diabetic foot ulcers have included combinations of arginine, glutamine, and β-hydroxy-β-methylbutyrate, although current evidence from randomized trials remains uncertain regarding their efficacy for improving ulcer healing.^256^ Collectively, these findings cement glutamine as a critical metabolic nexus whose homeostasis is intricately linked to insulin action, whose dysregulation propagates diabetic complications, and whose pathways offer actionable targets for dietary, microbial, and pharmacological intervention in cardiometabolic disease.^56,202^

### Roles in lipid metabolism, hepatic steatosis, and obesity-associated inflammation

Glutamine’s role as a metabolic nexus extends profoundly into the regulation of lipid homeostasis, the pathogenesis of hepatic steatosis, and the chronic low-grade inflammation characteristic of obesity. In states of metabolic dysfunction, circulating glutamine levels are frequently altered^257^; patients with obesity or T2DM exhibit decreased serum concentrations of glutamine and its downstream metabolite α-KG, alongside increased levels of succinate (Fig. 4b).^202^ This metabolic signature suggests a rewiring of glutamine pathways that contributes to disease pathology. In MASLD, hepatic glutaminolysis is markedly increased. PET imaging using l-[5-11C]glutamine ([11C]Gln) demonstrates significantly elevated hepatic uptake in MASLD mice, which is attenuated upon therapeutic inhibition of GLS1.^258^ This increased avidity for glutamine is driven by activated, glutamine-addicted hepatic stellate cells (HSCs), the primary fibrogenic cells in the liver. During MASLD fibrosis progression, HSCs upregulate the glutamine transporter SLC1A5 and the kidney-type GLS1, shifting the hepatic glutamine metabolic landscape.^259^ The resulting increase in the serum glutamate-to-glutamine ratio correlates with fibrosis severity and myofibroblast content, positioning glutaminolysis as both a diagnostic marker and a therapeutic target in active hepatic scarring.^259^ Abnormal glutamine metabolism reduces GSH synthesis and weakens liver antioxidant capacity. The GSH level in the liver tissue of MAFLD patients is significantly reduced, accompanied by the accumulation of ROS, which aggravates mitochondrial dysfunction and lipid peroxidation.^260^

The interplay between glutamine and lipid metabolism in the liver is complex and context dependent. In a rat model of fructose-induced MASLD, supplementation with specific amino acids under isonitrogenous conditions revealed that while arginine and glutamine were ineffective, citrulline attenuated hepatic fat accumulation and hypertriglyceridemia.^261^ This suggests that the benefits of nitrogenous precursors may not be interchangeable and that glutamine’s impact on hepatic lipids may be modulated by other metabolic pathways. Conversely, in HCC under glutamine deprivation, tumor cells activate lipophagy to mobilize cholesterol from lipid droplets, which subsequently inhibits de novo cholesterol synthesis as a survival mechanism to maintain redox balance.^262^ This metabolic plasticity underscores glutamine’s role in coordinating lipid and redox metabolism. Furthermore, interventions that modulate the gut-liver axis can influence hepatic steatosis through glutamine-related pathways. For instance, Lacto-N-triose II (LNT II) ameliorates high-fat diet-induced MASLD in mice by enhancing a microbiota-dependent glutamine-citrulline synthesis pathway, with serum citrulline levels negatively correlating with disease progression in human cohorts (Fig. 4b).^263^ Similarly, peanut skin procyanidins (PSPs) improve hepatic lipid metabolism in obese mice, an effect linked to increased hepatic glutamine levels and remodeled gut microbiota.^264^ Short-chain fatty acids (SCFAs) and ammonia produced by gut microbiota affect liver glutamine metabolism through the portal vein. The imbalance of intestinal flora in patients with MAFLD—characterized by reduced *Bacteroidetes* and elevated *Firmicutes*—promotes the entry of endotoxin (lipopolysaccharide) into the bloodstream. Endotoxin activates hepatic Kupffer cells, triggering the release of inflammatory factors and inhibiting GS activity. The resulting increase in glucose levels enhances hepatocyte proliferation and survival.^265,266^

In obesity, white adipose tissue becomes a site of chronic inflammation, characterized by macrophage infiltration and a shift toward a pro-inflammatory M1 phenotype. Glutamine metabolism is a key regulator of macrophage polarization.^202^ The observed decrease in serum glutamine and α-KG in obesity may thus create a milieu conducive to sustained adipose tissue inflammation. Dietary glutamine supplementation in high-fat diet-fed obese mice counteracts these alterations, attenuating body weight gain, fat mass, and adipocyte hypertrophy.^267^ This supplementation reduces macrophage infiltration and the expression of inflammatory cytokines in adipose tissue while also shifting adipose-derived mesenchymal stem cells toward a less hypermetabolic, quiescent state.^267^ The anti-inflammatory effects of glutamine are further demonstrated in obese mice undergoing bariatric surgery, where postoperative intravenous glutamine administration improves glucose tolerance and downregulates the expression of inflammatory cytokines and macrophage markers in adipose depots.^251^

The therapeutic targeting of glutamine metabolism in obesity and fatty liver disease holds promise but requires careful consideration of metabolic context and potential off-target effects. Inhibitors of GLS, such as BPTES and CB-839, have shown efficacy in reducing hepatic glutaminolysis and improving liver damage in MASLD models.^258,259^ However, the metabolic dependencies of different cell types can vary. For instance, while inhibiting GLS may curb HSC activation, the unique reliance of certain tissues on glutamine metabolism necessitates caution. Canagliflozin exemplifies an unintended metabolic disruption, disrupting glutamine anaplerosis and posing a potential risk for cytotoxicity in proliferating renal tubular cells.^255^ Therefore, strategies that selectively target glutamine metabolism in specific pathological cell populations, such as fibrogenic HSCs or proinflammatory macrophages, or those that employ glutamine supplementation to restore beneficial metabolic and immune functions may offer more refined therapeutic avenues for treating lipid metabolism disorders, hepatic steatosis, and obesity-associated inflammation.^202,259,267^

### Impact on vascular function, atherosclerosis, and cardiac metabolism

The intricate relationship between glutamine metabolism and cardiovascular health extends beyond its roles in hepatic steatosis and obesity-associated inflammation, critically influencing vascular cell behavior, atherosclerotic plaque progression, and cardiac function. Epidemiological and clinical metabolomic studies consistently highlight glutamine and its metabolite glutamate as significant players in CVD risk. In a large case-cohort study from the PREDIMED trial, higher baseline plasma glutamate levels were associated with a 43% increased risk of composite CVD and an 81% increased risk of stroke, whereas a higher glutamine-to-glutamate ratio was associated with a 25% and 44% decreased risk for these outcomes, respectively.^268^ These findings are corroborated by prospective cohort studies showing that higher dietary intake of glutamine and a higher dietary glutamine-to-glutamate ratio are inversely associated with total and cardiovascular mortality.^269^ This suggests that a metabolic imbalance favoring glutamate over glutamine may be a harbinger of vascular pathology. The clinical relevance is underscored by the finding that participants with high baseline glutamate obtained greater cardiovascular benefits from a Mediterranean diet intervention.^268^ Mechanistically, this imbalance may reflect underlying disruptions in critical metabolic pathways. In patients scheduled for coronary artery bypass grafting, both plasma and urinary glutamine levels were significantly lower in the high cardiovascular risk group, and a pro-atherosclerotic stimulus decreased secreted glutamine from human aortic vascular smooth muscle cells (HA-VSMCs).^270^ These observations position glutamine and glutamate homeostasis as a central metabolic nexus in vascular health.

At the cellular level, glutamine metabolism directly orchestrates vascular cell phenotype and function, contributing to vascular remodeling and stiffness. In VSMCs, glutamine dose-dependently promotes proliferation, migration, and a phenotypic switch from a contractile to a synthetic state, characterized by decreased expression of markers such as MYH11 and calponin and increased expression of collagen I and vimentin (Fig. 4a).^271^ This glutamine-driven phenotypic modulation, which can be attenuated by the GLS inhibitor BPTES, operates through a novel pathway involving the downregulation of miR-143 and consequent upregulation of its target, THY1 (Thy-1 cell surface antigen).^272^ Such synthetic, proliferative VSMCs are key contributors to neointima formation and arterial stiffening. Vascular stiffness, a hallmark of pathologies such as pulmonary hypertension (PH), is driven by excessive collagen deposition by activated fibroblasts. Research shows that in pulmonary arterial adventitial fibroblasts, the transcriptional coactivators YAP/TAZ, activated by inflammation and mechanical stress, drive glutamine and serine catabolism to fuel the biosynthesis of proline and glycine, the primary amino acids in collagen.^273^ Dietary or pharmacologic intervention targeting this anabolic demand reduced vascular stiffening and improved cardiovascular function in PH models, directly linking glutamine metabolism to the structural pathology of vessels.^273^ In the context of radiation therapy, a known risk factor for delayed CVD, irradiation-induced metabolic alterations in vascular cells, including perturbations in glutamine metabolism, are implicated in driving cellular senescence and chronic inflammation, perpetuating vascular damage.^274^ Endothelial cell function is equally sensitive to glutamine metabolism. Oxidative stress rapidly modulates endothelial glutamine catabolism via the redox-sensitive inhibition of the enzyme nitrilase-like 2 ω-amidase (NIT2), which converts α-ketoglutaramate (α-KGM) to α-KG; impairment of this pathway elevates α-KGM, disrupts angiogenesis, and promotes endothelial senescence.^275^ Conversely, in pulmonary arterial hypertension, pathological endothelial proliferation is supported by a NOX1-Sp1-CXCL12 axis that induces GLS activity alongside glucose metabolism.^276^ Thus, glutamine metabolism is a critical regulator of both endothelial and smooth muscle cell fate in the vasculature.

The progression of atherosclerosis and arterial calcification, the principal pathological substrates of most cardiovascular events, is profoundly influenced by glutamine flux and its enzymatic regulation.^277^ Population-based studies such as the MESA cohort have identified the plasma glutamine-to-glutamate ratio as an independent risk factor for carotid plaque progression.^278^ This clinical observation is mechanistically supported by studies highlighting the role of specific glutamine-metabolizing enzymes. GLS1 is upregulated in calcified arteries, and its activity correlates with arterial calcification progression in chronic kidney disease patients.^279^ Glutamate, the product of GLS1, accelerates osteogenic reprogramming of VSMCs by activating N-methyl-D-aspartate receptors (NMDAR), triggering calcium influx and β-catenin nuclear translocation, a pathway that can be mitigated by GLS1 inhibition.^279^ In contrast, GLS2 appears to have a protective role. Mice deficient in *Gls2* developed accelerated atherosclerosis and catastrophic cardiac events, while *Gls2* overexpression was protective.^278^ This protection was linked to GLS2’s role in maintaining systemic glutamine homeostasis, thereby preserving the extracellular matrix composition and preventing excessive vessel stiffness in atherosclerotic plaques.^278^ Beyond the arterial wall, glutamine metabolism within plaque macrophages is pivotal. Single-cell RNA sequencing studies of atherosclerotic mice and human plaques identified *Slc7a7*, a glutamine transporter, as a marker of lipid-associated macrophages; its knockout inhibited oxidized LDL uptake and dampened the foam cell gene signature, suggesting that glutamine signaling is critical for this key atherogenic event (Fig. 4a).^280–282^ Furthermore, dietary or metabolite interventions can modulate atherosclerotic risk. For example, in rats, acrylamide exposure induced cardiometabolic toxicity associated with disruptions in alanine, aspartate, glutamate, and D-glutamine/D-glutamate metabolism pathways, which were ameliorated by epigallocatechin gallate supplementation.^283^ Therefore, targeting glutamine uptake or metabolism in specific vascular and immune cell populations presents a promising therapeutic strategy for atherosclerosis.

In cardiac metabolism and the setting of heart failure, glutamine influences both myocardial health and systemic complications.^284^ Glutamine is known to induce antioxidant proteins such as heme oxygenase-1 and heat shock proteins, which can mitigate myocardial infarction and ischemia‒reperfusion injury.^14,285^ Metabolomic insights from the Framingham Heart Study revealed that the association between an ideal cardiovascular health (CVH) score and incident heart failure was partially mediated by a set of metabolites, including glutamine, implicating pathways of alanine, glutamine, and glutamate metabolism in heart failure pathogenesis.^286^ In chronic stable heart failure, a condition characterized by skeletal muscle wasting and exercise intolerance, combined supplementation with L-alanyl-L-glutamine and fish oil for three months did not improve exercise performance but significantly increased lean body mass and improved quality of life.^287^ This suggests a role for glutamine in counteracting the catabolic state associated with heart failure, even if functional capacity is not immediately enhanced. The vascular complications of other systemic diseases also involve glutamine pathways. In sickle cell disease, an FDA-approved therapy with L-glutamine aims to reduce vaso-occlusive crises, although real-world uptake remains low.^288^ Interestingly, hepatic involvement in sickle cell disease often presents with portosinusoidal vascular disorder, characterized by aberrant sinusoidal staining and decreased zone 3 GS reactivity, linking local hepatic glutamine metabolism dysfunction to microvascular pathology.^289^ Furthermore, during embryonic development, maternal metabolic dysregulation affecting glutamine metabolism can have profound consequences. Gestational inhibition of GLS or knockdown of *Cps1* (involved in the urea cycle utilizing glutamine-derived nitrogen) in mice increased the incidence of congenital heart disease in offspring, a defect rescued by carbamylglutamate supplementation, highlighting an essential role for glutamine-derived metabolites in epigenetic regulation of cardiac morphogenesis.^290^ SLC1A5 protects against aortic aneurysms and dissection through glutaminolysis -epigenetic coordination of vascular smooth muscle cell (VSMC) homeostasis.^291^

The intersection of glutamine metabolism with IR and metabolic syndrome forms a critical pathway linking obesity and diabetes to accelerated cardiovascular disease. Insulin signaling in endothelial cells normally promotes nitric oxide (NO) production, supporting vasodilation and vascular health (Fig. 4a). In IR, this pathway is impaired, contributing to endothelial dysfunction, a key early step in atherogenesis.^292,293^ The role of glutamine in this context is dual-edged. On one hand, glutamine can serve as an L-arginine precursor to support NO synthesis, suggesting potential vasoprotective effects.^14^ On the other hand, the metabolic disturbances of IR may alter glutamine flux in vascular cells. While the direct causal link to glutamine metabolism requires further elucidation, the associated metabolic stress can disrupt amino acid homeostasis. Elevated uric acid, often seen in metabolic syndrome, interacts with IR to synergistically increase arterial stiffness, as measured by pulse wave velocity.^294^ Given that hyperuricemia in rat models is associated with downregulated serum glutamate and glutamine levels and disrupted gut microbiota nitrogen cycling,^295^ a connection between purine metabolism, amino acid balance, and vascular health is plausible. Thus, the perturbed glutamine-glutamate homeostasis observed in cardiovascular diseases likely both contributes to and is exacerbated by the underlying insulin-resistant, proinflammatory milieu of metabolic syndrome, creating a vicious cycle that promotes atherosclerosis and vascular damage.^278,292^

## Organ-specific fibrosis and injury

### Hepatic fibrosis

The progression of chronic liver diseases toward organ failure is intrinsically linked to the fibrogenic process, a dynamic and integrated response to persistent parenchymal injury.^296,297^ Central to this fibrogenic cascade is the activation of hepatic stellate cells (HSCs). While the inflammatory milieu and cytokines, such as TGF-β, that initiate HSC activation are well documented,^298^ recent research has unequivocally established that metabolic reprogramming, particularly amino acid metabolism, is not merely a consequence but a fundamental driver of their profibrotic phenotype. Within this metabolic landscape, glutamine metabolism emerges as a critical nexus, intimately connecting the bioenergetic and biosynthetic demands of activated HSCs with the broader hepatic dysregulation of ammonia homeostasis, thereby creating a self-perpetuating cycle that fuels fibrogenesis.^259,299^

Metabolic rewiring in activated HSCs supports their high proliferative and synthetic rates. Recent work has identified lectin-like oxidized low-density lipoprotein receptor-1 (LOX-1) as a key upstream regulator that exacerbates liver fibrosis by promoting HSC activation through reprogramming glutamine metabolism.^299^ The LOX-1/OCT1 (organic cation transporter 1) axis mediates the enzyme switching from GLS2 to GLS1, establishing a direct link between a pathological receptor and metabolic reprogramming in fibrogenesis.^299^ Furthermore, lactate dehydrogenase (LDH) inhibition has been shown to impede glutamine metabolism in HSCs through a lactic acid/HDAC/histone acetylation/c-Myc signaling pathway, highlighting the intricate interplay between glycolytic and glutaminolytic fluxes in maintaining the activated state.^300^

Concurrent with and functionally linked to the reprogramming of glutamine metabolism in HSCs is a profound disruption of hepatic ammonia homeostasis. The liver is the primary site for ammonia detoxification via the urea cycle in periportal hepatocytes and via glutamine synthesis catalyzed by GS in a small perivenous hepatocyte population. In fibrosis, this meticulous zonation is lost.^259,301^ Hyperammonaemia is not merely a complication but an active contributor to the fibrogenic milieu. It drives the liver-muscle axis in cirrhosis, leading to sarcopenia via mechanisms that include cataplerosis of α-KG in muscle, further intertwining nitrogen metabolism with systemic metabolic dysfunction.^302^ Within the liver, ammonia itself can influence fibrogenesis. Research indicates that LOX-1 rewires ammonia metabolism by regulating the glutamine metabolism-urea cycle interface to drive fibrosis progression.^299^ Furthermore, in the context of MASLD, disrupted RNA splicing leads to activation of reductive carboxylation of glutamine for lipogenesis, a process that causes hepatic ammonia accumulation, thereby initiating hepatic fibrosis; notably, ammonia clearance was shown to block this fibrogenesis.^303^ The detoxification of ammonia via glutamine synthesis becomes crucial. Adipose-derived stromal cell exosomes ameliorate hepatic fibrosis by upregulating hepatocellular GS and remodeling-associated ammonia metabolism, an effect abolished by a GS inhibitor, underscoring the therapeutic importance of this pathway.^301^ Similarly, in alcohol-related liver disease (ALD), a soluble fiber-rich diet enriches *Bacteroides acidifaciens*, which activates intestinal FXR-FGF15 signaling to promote hepatocyte expression of ornithine aminotransferase (OAT); this facilitates ornithine metabolism into glutamate, providing substrate for ammonia detoxification via glutamine synthesis and ameliorating injury.^304^ The gut-liver axis is paramount in ammonia production, with colonic urease-producing bacteria being a major source. Inhibiting this microbial urease activity with agents such as 2-octynohydroxamic acid reduces ammonemia and cerebellar glutamine levels in bile duct ligated rats, presenting a novel therapeutic avenue for hepatic encephalopathy and potentially impacting the fibrotic environment.^305^ This aligns with findings that alcohol-associated dysbiosis involves downregulated microbial glutamate/glutamine biosynthesis pathways.^306^ The recognition of glutamine metabolism and ammonia handling as core pathways in fibrogenesis opens promising therapeutic frontiers.

### Pulmonary and renal fibrosis

The paradigm of metabolic reprogramming extends to critically underpin the pathogenesis of fibrotic diseases in the lungs and kidneys. In idiopathic pulmonary fibrosis, the differentiation of lung fibroblasts into collagen-producing myofibroblasts is driven by TGF-β and is intrinsically linked to a rewiring of glutamine metabolism.^107^ This metabolic shift is not merely correlative but functionally essential; glutamine and its conversion to glutamate by GLS are strictly required for TGF-β-induced collagen protein synthesis in lung fibroblasts.^107^ Intriguingly, this requirement is dissociated from canonical bioenergetic roles. The metabolism of glutamate to α-KG for oxidation in the mitochondria is dispensable for collagen production, as inhibition of glutaminolysis does not affect cellular oxygen consumption and knockdown of oxoglutarate dehydrogenase fails to impair fibroblast collagen synthesis.^107^ Instead, the critical function of glutamine metabolism lies in fueling anabolic biosynthesis. Glutamate is utilized by specific enzymes to generate the amino acid building blocks of collagen: phosphoserine aminotransferase 1 (PSAT1) is required for de novo glycine production, and aldehyde dehydrogenase 18A1 (ALDH18A1)/Δ1-pyrroline-5-carboxylate synthetase (P5CS) is required for de novo proline production.^107^ Accordingly, TGF-β treatment increases cellular concentrations of glycine and proline, positioning glutamine catabolism as a direct supplier of precursors for ECM construction, thereby revealing amino acid biosynthesis pathways as novel therapeutic targets for fibrotic diseases.^107,307^

The regulation of this profibrotic metabolic circuit involves both intracellular and extracellular mediators. Recent proteomic and single-cell analyses identified cathepsin K (CTSK) as a novel regulator that accumulates in fibroblasts within fibrotic lungs.^308^ Recombinant CTSK exacerbates collagen accumulation and disease progression in murine models of pulmonary fibrosis. Mechanistically, CTSK undergoes endocytosis by sorting nexin 9 (SNX9), which engages TGF-β1-induced SMAD3 activation to drive the upregulation of GAC (GLS1 isoform) and subsequent glutamine enrichment in fibroblasts, thereby promoting collagen synthesis.^308^ This pathway has clinical relevance, as serum CTSK levels in patients with pulmonary fibrosis positively correlate with circulating glutamine levels and poor prognosis, suggesting its potential as a predictive biomarker.^308^ Beyond precursor supply, glutaminolysis also exerts epigenetic control over the fibrotic phenotype. In IPF fibroblasts, enhanced glutaminolysis and the resulting metabolite α-KG, a cofactor for histone demethylases, modulate the levels of the repressive histone mark H3K27me3 at the promoters of key profibrotic genes such as *COL3A1*.^309^ Deprivation of glutamine or silencing of GLS1 alters this epigenetic landscape and suppresses the pro-fibrotic gene expression profile and cell phenotype, effects only partially rescued by exogenous α-KG supplementation, indicating both α-KG-dependent and -independent mechanisms.^309^ This is corroborated in vivo, where interventions that reduce glutaminolysis markedly attenuate fibrotic markers in a murine model of lung fibrosis.^309^

In the kidney, the activation and proliferation of fibroblasts are equally central to the progression of renal interstitial fibrosis (RIF), and glutamine metabolism emerges as a critical facilitator of this process.^310^ Patients with severe renal fibrosis exhibit elevated serum glutamine levels and increased expression of kidney GS, suggesting dysregulated glutamine flux.^310^ Experimentally, deprivation of glutamine metabolism in vitro and in vivo robustly inhibits fibroblast activation and ameliorates renal fibrosis. The mechanism extends beyond mere precursor supply to the regulation of cellular energetics and organelle dynamics. Glutamine metabolism is crucial for maintaining mitochondrial function and morphology in renal fibroblasts, and the effects are partly dependent on the metabolic intermediate α-KG. Furthermore, glutamine deprivation upregulates mitochondrial fission through the mTOR/MTFP1/DRP1 pathway, linking glutamine catabolism to the control of mitochondrial architecture, a process essential for supporting the bioenergetic and biosynthetic demands of activated fibroblasts.^310–312^

A distinct but related pathological process involving aberrant epithelial growth and stromal expansion is observed in polycystic kidney disease (PKD), where glutamine dependency is a prominent metabolic vulnerability. Cyst-lining epithelial cells in autosomal-dominant polycystic kidney disease (ADPKD) exhibit upregulation of GLS1 and coordinated metabolic rewiring that includes increased glycolysis and preferential use of glutamine to fuel the TCA cycle and sustain fatty acid synthesis.^313,314^ This renders PKD cells exquisitely sensitive to glutamine deprivation or inhibition of its metabolism. Pharmacological inhibition of GLS1 with compounds such as CB-839 blocks forskolin-induced cyst growth in vitro and can slow disease progression in certain mouse models of ADPKD, such as the Aqp2-Cre; Pkd1fl/fl model, where it also inhibits pro-proliferative mTOR and MEK signaling.^313^ However, the efficacy is model dependent, as CB-839 failed to slow cyst growth in a Pkhd1-Cre; Pkd1^fl/fl^ model, highlighting potential compensatory metabolic adaptations that can bypass GLS1 inhibition.^313^ This metabolic plasticity is further evidenced by the role of ASNS, an enzyme that utilizes glutamine-derived nitrogen. In PKD, an ATF4-ASNS axis is upregulated, driving glutamine-dependent de novo pyrimidine synthesis to support cystic epithelial proliferation.^315^ Silencing of *ASNS* with antisense oligonucleotides in murine PKD models leads to a drastic reduction in kidney volume and rescue of renal function, and combining ASNS inhibition with a glycolytic inhibitor (2-deoxyglucose) yields even greater efficacy, revealing novel combinatorial metabolic vulnerabilities.^315^ The dependency on glutamine is also reinforced by genetic studies; coablation of *Lkb1* and *Tsc1* in the kidney results in aggressive PKD, and both *Lkb1*-deficient and *Pkd1*-deficient cells show a marked dependence on glutamine for growth, requiring it for NEAA and GSH metabolism.^316^ Consequently, inhibition of glutamine metabolism significantly reduces cyst progression in these genetic models, solidifying glutaminolysis as a key therapeutic target in PKD.^316^

The therapeutic implications of targeting glutamine metabolism in fibrosis are promising yet require careful consideration of context and delivery. In peritoneal dialysis, an RCT demonstrated that supplementing dialysis fluid with alanyl-glutamine for 8 weeks improved biomarkers of peritoneal membrane integrity and immune competence, suggesting a protective role against peritoneal membrane fibrosis and inflammation.^317^ However, the pleiotropic roles of glutamine as a nitrogen carrier, immune modulator, and neurotransmitter precursor necessitate a nuanced approach to its therapeutic manipulation, as both deficiency and excess can be pathological, as observed in critical illness.^318^ The development of advanced delivery systems, such as self-assembling nanomedicines designed to codeliver glutamine transport inhibitors with other therapeutics, represents an innovative strategy to achieve targeted metabolic disruption within fibrotic tissues.^319^

### Intestinal barrier function and inflammatory bowel diseases

**Fig. 5:**
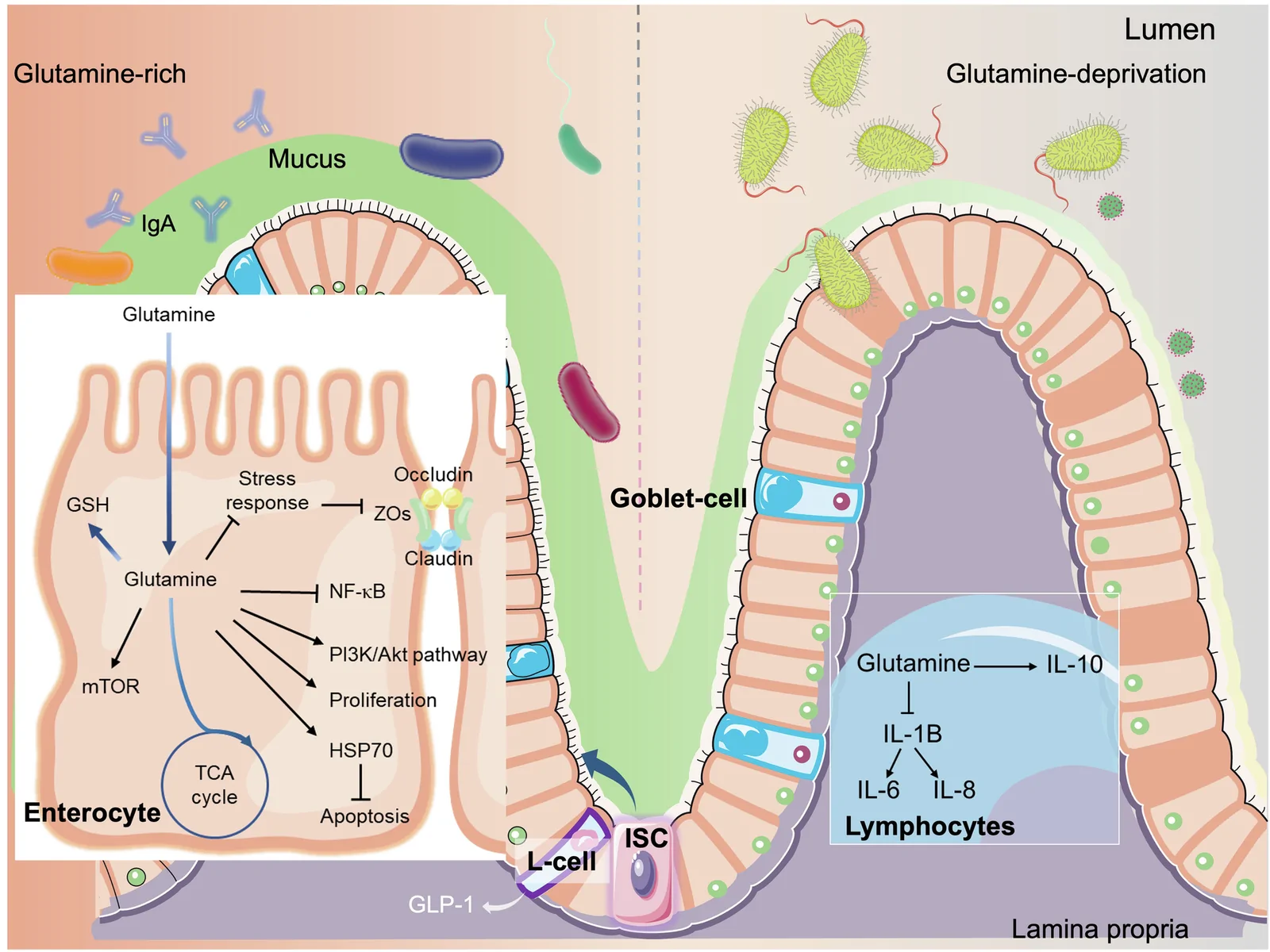
Protective effects of glutamine on the intestinal mucosa. Glutamine protects intestinal health by sustaining the balance of the microbiome. Glutamine increases enterocytes and goblet cells by promoting the differentiation of intestinal stem cells (ISCs). Glutamine can promote the secretion of GLP-1 by L-cell, which is of great significance for the sense of metabolic balance of the body. Glutamine increases the expression of tight junction proteins in enterocytes and promotes the secretion of mucins and IgA by goblet cells, which enhance the immune barrier of the intestinal mucosa. Glutamine also promoted the secretion of the anti-inflammatory cytokine IL-10 and inhibited the secretion of proinflammatory IL-1B by immune cells, thereby minimizing the inflammatory response

The centrality of glutamine metabolism serves as a primary respiratory fuel for enterocytes and a crucial regulator of intestinal barrier integrity (Fig. 5). Here, glutamine emerges not merely as a metabolic substrate but also as a functional linchpin connecting mucosal homeostasis, microbial ecology, and inflammatory signaling.^320–322^ Glutamine promotes the differentiation of intestinal stem cells and proliferation of epithelial cells.^323^ The intestinal epithelium exhibits a high demand for glutamine to maintain its rapid turnover and functional integrity. Metabolic flux analyses in human intestinal epithelial cells (IECs) reveal a complex interplay between glucose, glutamine, and fatty acid oxidation, with perturbations—such as exposure to environmental toxins such as perfluorooctanoic acid (PFOA)—inducing a metabolic rewiring that enhances glutaminolysis and reductive carboxylation to support biomass synthesis and potentially contribute to cellular stress and dysfunction.^321^ In addition, glutamine is utilized by goblet cells to produce mucus and by plasma cells to produce IgA, hence providing various protective functions.^324,325^ This dependency is exploited therapeutically, as evidenced by studies showing that enteral nutrition supplemented with glutamine significantly improves nutritional status, serum protein levels, and mucosal expression of the tight junction protein occludin in rat models following ileal pouch-anal anastomosis, a common surgical intervention for ulcerative colitis (UC).^322^ In a small, randomized, double-blind, placebo-controlled, 8-week trial, oral dietary glutamine supplementation significantly and safely reduced all IBS-related primary endpoints in patients with IBS and hyperpermeability of the intestine following intestinal infections.^326^

The reinforcement of the intestinal barrier by glutamine is mediated through multiple mechanisms. Glutamine supplementation has been shown to protect against increased villus height, reduced crypt depth, enhanced expression of tight junction proteins, and a modulated inflammatory response with elevated anti-inflammatory interleukin-10 (IL-10).^320,327,328^ This direct effect on epithelial structure is complemented by glutamine’s role in modulating systemic and local inflammation. Intravenous glutamine administration was shown to attenuate the proinflammatory response to LPS.^329^ This immunomodulatory capacity is crucial in IBD, where uncontrolled inflammation drives tissue damage. Clinically, a dietary supplement combining specific probiotic strains with L-glutamine and biotin administered to UC patients led to significant improvements in body composition parameters, including increased muscle mass and a normalized extracellular water ratio, which were strongly correlated with an enhanced disease-specific quality of life.^330^ Furthermore, a retrospective comparative study in patients with external duodenal fistula demonstrated that low-dose (10 g/day) oral glutamine supplementation was associated with a higher rate of spontaneous fistula closure and a significantly reduced hospital stay, highlighting its role in promoting mucosal healing in a challenging clinical scenario.^331^

Glutamine supplementation may improve intestinal immunity through intestinal microbial metabolites.^332^ The gut microbiota intricately regulates glutamine metabolism through microbial metabolites, signaling pathways, and host–microbe interactions.^333,334^ SCFAs from the breakdown of dietary fiber by gut microbes can affect the metabolism of glutamine. For example, butyrate, as a basic energy source in intestinal epithelial cells, can upregulate the expression of GS and inhibit the activity of GLS through a variety of pathways, ultimately increasing the total amount of glutamine that can enter the circulation.^335–337^ Membrane vesicles derived from probiotics, including *Lactobacillus*, modulate macrophage activity by targeting the GS/mTORC1 signaling pathway.^338,339^ Leng et al.^340^ found that probiotics prevent severe intestinal barrier dysfunction by activating intestinal microbiota-mediated biosynthesis of glutamic-glutamine organisms.

Dietary glutamine can be used by intestinal bacteria, such as *B. ovatus*, to affect the abundance and metabolites of intestinal flora, thereby affecting or promoting human health.^341–343^ Specific microbial populations, such as *Akkermansia* and *Bacteroidaceae*, have been associated with the modulation of glutamine levels in the gut, influencing metabolic profiles and potentially providing protective effects against metabolic disorders.^344,345^ Several studies in small samples and animal models have found that glutamine supplementation combined with probiotics can reduce inflammation and alleviate gastrointestinal diseases,^346,347^ significantly reduced infection rates and shorten intensive care unit stays for patients with traumatic brain injuries.^348^ The integrity of the gut barrier, assessed via plasma tight junction proteins such as Claudin-5, has been strongly associated with microbial metabolic pathways involving D-glutamine and D-glutamate metabolism, underscoring direct metabolic crosstalk.^349^ Certain bacterial species can directly influence host glutamine metabolism to benefit the barrier. For instance, *Cetobacterium somerae*, a bacterium enriched by a plant-based protein diet, was found to promote goblet cell differentiation and enhance intestinal barrier function in a fish model. This effect was mechanistically linked to the bacterium’s ability to elevate intestinal glutamine levels, which subsequently suppressed the Notch signaling pathway, a key regulator of secretory cell fate.^350^ Conversely, gut dysbiosis associated with conditions such as major depressive disorder (MDD) features a reduction in SCFA-producing genera such as *Collinsella* and is correlated with elevated plasma markers of gut barrier dysfunction (Claudin-5, zonulin) and systemic inflammation, with these pathways again implicating perturbations in glutamate and glutamine metabolism.^349,351^ In summary, dietary glutamine modulates the composition of the gut microbiota, which in turn influences glutamine metabolism and ultimately impacts overall health.

This microbiota-glutamine axis also has significant diagnostic and systemic implications. Plasma citrulline, a nonprotein amino acid derived almost exclusively from intestinal glutamine metabolism in enterocytes, is an established biomarker for functional enterocyte mass.^352^ Its production is highly sensitive to intestinal health, as demonstrated in exercise studies where the postglutamine bolus rise in plasma citrulline was blunted following strenuous, dehydrating exercise—a state associated with increased intestinal injury markers such as iFABP.^352^ Importantly, interventions that bolster this pathway, such as supplementation with the prebiotic lacto-N-triose II, can enhance gut microbiota-dependent citrulline synthesis, which correlates with improved metabolic parameters in liver disease models, illustrating a gut-liver axis mediated by glutamine-derived metabolites.^263^

At present, research on the relationship between microorganisms and metabolism is still in the initial stage, and the model animals and small sample tests are not enough to elucidate the mechanism, the conditions for clinical benefit and the duration of clinical benefit, which is worthy of further study.

## Glutamine in neurological and psychiatric disorders

### The glutamate/GABA-glutamine cycle

**Fig. 6:**
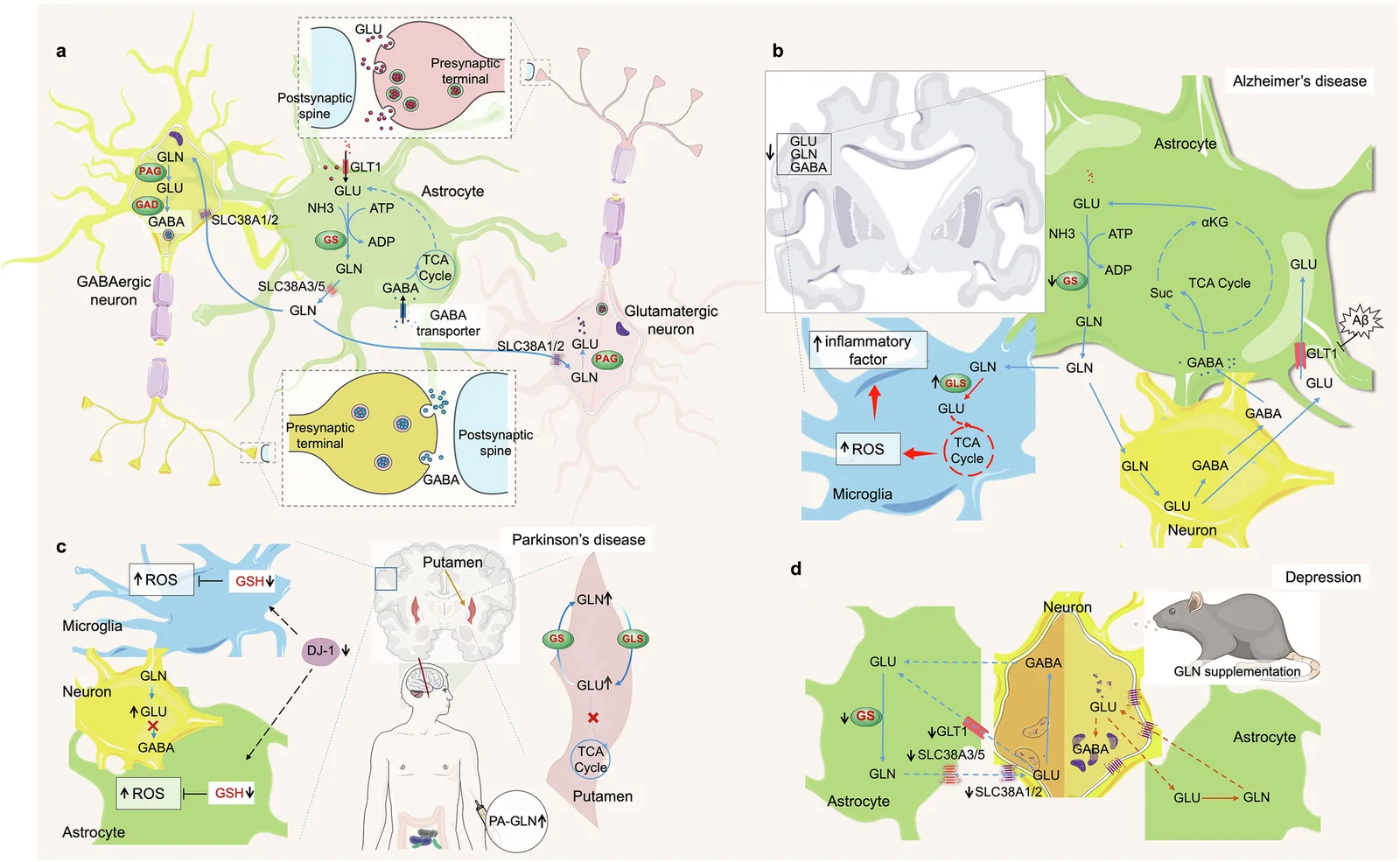
Glutamine (GLN) homeostasis is disrupted in Alzheimer’s disease and depression. **a** Glutamine is central to brain function through the glutamate/GABA-glutamine cycle, where glutamate (GLU) (a key excitatory neurotransmitter) is released into the synapse and then removed by astrocytes. Inside astrocytes, glutamate is converted to glutamine by the enzyme glutamine synthetase (GS). Glutamine is then transported back to neurons, where it is converted back to glutamate by phosphate-activated glutaminase (PAG) to replenish the synaptic pool, ensuring continued neurotransmission and proper cognitive function. Apart from glutamate recycling, synaptic GABA is transported into astrocytes and sequentially degraded by GABA transaminase and succinic semialdehyde dehydrogenase. Metabolic intermediates derived from GABA support astrocytic glutamine synthesis. **b** Hampered astrocyte glutamine synthesis and hyperactive glutaminolysis in Alzheimer’s disease. Glutamate, glutamine and GABA levels were all significantly reduced in cerebral cortical tissue. Thick red arrows: upregulated pathways; Thin blue arrows: downregulated pathways. **c** Elevated glutamate, decreased GSH and GABA, aggravated oxidative damage and reduced energy in Parkinson’s disease. Phenylacetyl-glutamine (PA-GLN), which is involved in the metabolism of intestinal flora, is increased in the peripheral blood. The reduction in DJ-1 affects the synthesis of GSH within microglia and astrocytes, leading to oxidative damage. **d** Glutamate and glutamine transporters are reduced in depression, and glutamine supplementation increases their expression. Mitochondrial dysfunction leads to reduced mitochondrial energy in depression. Glutamine supplementation induces extensive mitophagy activation, which is important for the maintenance of mitochondrial function. GABA γ-aminobutyric acid, GAD glutamate decarboxylase, GDH glutamate dehydrogenase, GS glutamine synthetase, GSH glutathione, Suc succinate

Glutamine is the most abundant free amino acid in the CNS and is the direct precursor of the neurotransmitters glutamate and GABA (Fig. 6).^353^ Neurons release glutamate (or GABA) for synaptic signaling, which is subsequently taken up by perisynaptic astrocytes, converted to glutamine via GS, and shuttled back to neurons to replenish neurotransmitter pools (Fig. 6a).^354–356^ This cycle is critically dependent on the transport systems. Glutamine is transported out of astrocytes via SLC38A3/5 and into neuronal cells via SLC38A1/2. The neurotransmitters glutamate and GABA released from neuronal cells pass through GLT1 and GABA transporters, respectively, into astrocytes for glutamine synthesis.^357^ Recent genetic evidence has fundamentally challenged the long-held dogma that brain glutamine is exclusively synthesized locally, demonstrating that SLC38A3, expressed at the BBB vascular endothelium, is essential for mediating blood-to-brain influx of glutamine.^358^ Endothelial-specific deletion of *Slc38a3* in mice lowered brain glutamine levels, impaired the transfer of glutamine carbons to glutamate and GABA, and led to postnatal progressive microcephaly, seizures, and behavioral deficits—a phenotype that was rescued by glutamine supplementation, establishing blood-derived glutamine as a crucial substrate for neurotransmitter synthesis and synaptogenesis.^358^ This highlights a critical dependency on systemic glutamine availability, with disruptions in its transport manifesting as severe neurodevelopmental disorders.

Within the parenchyma, the glutamate/GABA-glutamine cycle is intimately coupled to astrocytic bioenergetics.^359^ Astrocytes not only take up synaptic glutamate but also metabolize it, with nitrogen being incorporated into glutamine and the carbon skeleton entering the TCA cycle. This metabolic integration is governed by complex signaling networks. For instance, the Wnt coreceptor LRP6 on astrocytes acts as a metabolic switch, whose absence shifts glucose metabolism via an mTOR-AKT axis toward increased catabolism of glutamine and branched-chain amino acids (BCAAs), potentially exhausting precursors for neurotransmitter recycling and priming the brain for cognitive decline, as seen in late-onset AD.^360,361^ This cycle not only maintains neurotransmitter homeostasis but also prevents excessive accumulation of glutamate in the synaptic cleft (excitotoxicity).^362^ The cycle is also paramount for inhibitory neurotransmission, as astrocytes regulate GABAergic signaling through uptake, metabolism, and recycling via the GABA-glutamine cycle, supplying neurons with glutamine as the primary precursor for GABA synthesis (Fig. 6a).^356^

### Neurodegenerative diseases

The critical role of glutamine metabolism within the glutamate/GABA-glutamine cycle becomes disrupted in neurodegenerative disorders. Alterations in glutamine synthesis, transport, and utilization are observed in AD and Parkinson’s disease (PD), reflecting and contributing to the complex pathophysiology involving excitotoxicity, metabolic failure, and chronic neuroinflammation.^355,362,363^ Advanced imaging and metabolic profiling now consistently identify glutamine and glutamate imbalances as early and progressive features, offering both mechanistic insights and potential therapeutic entry points.^364–366^

#### Alzheimer’s disease

In AD, a central failure emerges within the astrocytic compartment of the glutamate-glutamine cycle (Fig. 6b). Emerging evidence implicates dysregulated glutamine metabolism as a critical contributor to AD pathogenesis, influencing excitotoxicity, oxidative stress, and neuronal energy failure. In patients with AD, GS activity was significantly decreased, especially in the hippocampal and cortical regions. GLT1, the carrier of glutamate uptake by astrocytes, was also inhibited by amyloid-β (Aβ).^367,368^ This decrease will affect the recycling and reuse of glutamate, leading to the accumulation of glutamate in the synaptic cleft, inducing excitotoxicity and causing neuronal damage.^362,369^ This disruption likely contributes to the well-documented imbalance between excitation and inhibition in the AD brain. Supporting the translational relevance of these findings, in animal experiments, increasing the expression of GS in the brain or exogenous supplementation of glutamine can improve learning and memory ability and reduce Aβ deposition and tau protein hyperphosphorylation.^370^ A large-scale Mendelian randomization study identified that genetically predicted higher blood glutamine levels are associated with a decreased risk of AD, positioning glutamine as a potential protective factor and therapeutic target with a favorable side-effect profile.^371^ The metabolic support role of astrocytes is further compromised in AD, a deficit that can be partially overcome by alternative substrates such as decanoic acid, which efficiently label the astrocytic glutamine pool.^372–374^ Immunomodulatory interventions, such as glatiramer acetate in old AD mice, have been shown to restore homeostatic GS expression, linking immune modulation to astrocyte metabolic function.^375^ Pharmacologically, the drug riluzole improves cognitive function and neurotransmitter homeostasis in AD mice by upregulating key proteins in the glutamate-glutamine cycle, including GS and excitatory amino acid transporters, as revealed by combined CEST imaging and MRS.^376^ Studies at the cellular level confirmed that sulbactam protected neurons from the dual neurotoxicity of Aβ and glutamate loading by upregulating GLT1 expression.^368^ Natural compounds such as galantamine and lycorine also show distinct abilities to restore glutamine homeostasis in neuronal cells exposed to Aβ toxicity.^377^ On the other hand, excessive supplementation with glutamine may inhibit GS expression and activity, which suggests that supplementation with glutamine may not be a preventive measure for AD.^378^

Neuroinflammation, a hallmark of AD, is intricately linked to glutamine metabolism, particularly within microglia (Fig. 6b). The level of oxidative stress is generally increased in the brain tissue of AD patients, while the level of GSH is significantly decreased, which is closely related to the insufficient supply of glutamine.^379^ The activated astrocytes and microglia in the pathogenesis of AD will change the expression and distribution of GS, further aggravating the neuroinflammatory response.^380^ Activation of the NLRP3 inflammasome in microglia is a key driver of pathology, and this process is regulated by glutaminolysis.^370,380,381^ GLS activity is upregulated and the TCA cycle is weakened in the microglia of AD patients, resulting in decreased ATP synthesis, lactic acid accumulation, and reduced mitochondrial membrane potential.^382–386^ Under AD conditions, increased microglial glutamine metabolism fuels the TCA cycle, sustaining mTORC1 signaling, suppressing mitophagy, and promoting ROS accumulation, which collectively facilitate NLRP3 inflammasome activation.^380^ Conversely, chronic inhibition of NLRP3 enhances glutamine- and glutamate-related metabolism in microglia, increases the expression of the glutamate transporter SLC1A3, and boosts mitochondrial activity and Aβ clearance capacity.^370^ This suggests a bidirectional relationship where inflammatory signaling shapes the metabolic phenotype and vice versa. The broader landscape of AD-associated neuroinflammation involves complex, dysfunctional crosstalk between activated microglia and reactive astrocytes, both of which undergo phenotypic transitions that can be neurotoxic.^381,387–389^ Astrocytic dysfunction, including loss of fatty acid degradation capacity and subsequent lipid droplet accumulation, can drive neuroinflammation and neurodegeneration through mechanisms involving STAT3 acetylation and impaired neuronal support.^390^ Therapeutic strategies are increasingly targeting this inflammation, with compounds such as dimethyl fumarate (activating Nrf2), spermidine (inducing autophagy), naringenin (inhibiting MAPK), and luteolin (inhibiting ER stress) showing efficacy in reducing neuroinflammation and pathology in AD models by modulating glial cell function.^391–394^ The gut microbiota also emerges as a key modulator of this neuroinflammatory axis.^395^

#### Parkinson’s disease

Parkinson’s disease (PD) pathophysiology also involves perturbations in glutamine-related pathways, which are evident at both the systemic and molecular levels.^396,397^ Studies have shown elevated levels of glutamine and glutamate in the putamen, a part of the striatum, ipsilateral (same side) to the more affected side of the body in early-stage PD patients (Fig. 6c).^398,399^ Comprehensive plasma metabolomics in PD patients has identified a metabolite panel for discrimination that includes phenylacetyl-glutamine, a gut microbiota-derived compound, alongside alterations in fatty acids and bile acids.^400^ This highlights a potential intersection between peripheral metabolism, the gut-brain axis, and PD pathogenesis. At the cellular level, the excitatory effects of glutamate are enhanced due to damage to dopaminergic neurons, which may lead to excitotoxicity, damaging further neurons.^401,402^ Disruptions in the glutamate-glutamine cycle can contribute to this excitotoxicity and further aggravate neurological damage in PD.^398^ Loss of the PD-associated protein DJ-1 reduces the availability of precursors for GSH synthesis, which may lead to neurons being more susceptible to oxidative damage.^403–405^ Furthermore, DJ-1 deficiency in microglia promotes a proinflammatory state, creating a vulnerable environment for dopaminergic neurons.^403^ Insufficient energy supply may lead to apoptosis of neurons due to impairment of mitochondrial function.^406^ Disorders in glutamine metabolism may exacerbate this problem, affecting energy metabolism in the brain and then accelerating the progression of PD.^16^ The central proteinopathy of PD involves α-synuclein, whose aggregation into amyloid fibrils is a defining feature. The atomic structure of a pathogenic α-synuclein fibril reveals a “glutamine ladder” among other stabilizing interactions, suggesting that the glutamine residues in the protein sequence contribute to the robust propagation of the fibril form, a property shared by some early-onset PD mutants.^407^ Dietary approaches such as the ketogenic diet are proposed to benefit PD, partly through mechanisms that may include increasing the conversion of glutamine to the inhibitory neurotransmitter GABA.^408^

Across these neurodegenerative disorders, the interplay between glutamine metabolism and neuroinflammatory processes is a recurring theme, extending to other conditions such as amyotrophic lateral sclerosis (ALS) and multiple sclerosis (MS). In ALS, dipeptide repeat proteins from the C9orf72 expansion, rich in arginine, undergo liquid‒liquid phase separation and disrupt RNA metabolism and stress granule dynamics, while failure of RNA editing at a glutamine/arginine site in GluA2 mRNA contributes to excitotoxic motor neuron death.^409^ In progressive MS, longitudinal MRS studies show consistent and significant decreases in brain glutamate and glutamine concentrations over time, suggesting their potential as sensitive biomarkers of disease progression.^366^ These observations across the spectrum of neurodegeneration underscore that glutamine is not merely a metabolic intermediate but a dynamic modulator of cellular states, immune responses and proteostasis. Consequently, therapeutic strategies are evolving to target this nexus, from direct supplementation or restriction to modulating its enzymatic conversion, transport or its role in supporting specific cell types within the diseased brain microenvironment.

### Psychiatric disorders

#### Depression

The dysregulation of the glutamate-glutamine cycle, extending beyond classical neurodegenerative diseases, is a cardinal feature of major psychiatric disorders, where it interfaces with stress pathways, immune activation, and synaptic plasticity. In MDD, metabolomic profiling consistently identified perturbations in glutamatergic metabolites (Fig. 6d). The level of glutamine in brain areas such as the prefrontal cortex, anterior cingulate cortex and hippocampus of depressed patients has changed.^410^ It has been found that the expression of system A transporters (SLC38A1/2), system N transporters (SLC38A3/5), and GLT1 is altered in the brains of depressed patients.^411,412^ The abnormal expression of these transporters may lead to an imbalance in glutamine distribution between neurons and glial cells, affecting glutamine metabolism and neurotransmitter circulation. Studies have found that glutamatergic neurotransmission is enhanced in the brains of patients with depression, while GABAergic neurotransmission is weakened.^413^ This imbalance may lead to neural network dysfunction and then trigger depressive symptoms. In addition, abnormal glutamine metabolism may also affect the synthesis and metabolism of monoamine neurotransmitters, such as serotonin, norepinephrine, and dopamine, the dysregulation of which is an important pathological basis of depression.^414^ Plasma analyses reveal that the glutamine/glutamate ratio changes following treatment with selective serotonin reuptake inhibitors (SSRIs), specifically in responders, suggesting that normalization of glutamatergic tone may be linked to therapeutic efficacy.^415^ Broader metabolomic studies in treatment-naïve MDD patients corroborate this, showing decreased serum levels of glutamine among other metabolites, with these alterations persisting posttreatment and holding potential diagnostic value.^416^ This biochemical signature is not confined to peripheral compartments; proton magnetic resonance spectroscopy (1H-MRS) studies in the dorsolateral prefrontal cortex (DLPFC) of young adults from the general population have found that higher glutamine levels and glutamine/glutamate ratios correlate with neuroticism, a core personality trait and risk factor for mood and anxiety disorders, independent of clinical diagnosis.^417^ BDNF levels in the hippocampus and prefrontal cortex are reduced in patients with depression, which is associated with disorders of glutamine synthesis.^418,419^ In addition, abnormal glutamine metabolism may affect synaptic plasticity and neural network function by affecting the mTOR signaling pathway, leading to reduced synaptic protein synthesis.^43^ Studies have found decreased ATP production and impaired mitochondrial function in the brains of patients with depression, and these changes are related to disordered glutamine metabolism.^420^ Glutamine metabolism may also play a protective role in depression by regulating the antioxidant defense system through the nuclear factor erythroid 2-related factor 2 (Nrf2) pathway.^421,422^ Abnormal glutamine metabolism is closely related to neuroinflammation, which may be a new breakthrough direction for the management of depression.^423^ The central role of astrocytic GS in this pathology is becoming increasingly clear. Evidence indicates that hypoactivity of GS, potentially mediated by peroxynitrite-induced tyrosine nitration of the enzyme under chronic stress, is a key driver of depressive-like behaviors.^424^ Notably, interventions that enhance GS activity, such as supplementation with tyrosine or the dipeptide tyrosyl-glutamine, which facilitates tyrosine denaturation of GS, exert significant antidepressant effects in animal models.^424^ This GS-centric pathophysiology is further complicated by its interaction with systemic inflammation and the gut-brain axis. In adolescents with MDD, elevated plasma tight junction protein Claudin-5 is strongly linked to pathways of D-glutamine and D-glutamate metabolism, connecting gut barrier dysfunction and associated inflammation to central glutamatergic dysregulation.^349^ This connection is reinforced by findings that treatments such as hyperbaric oxygen or traditional Chinese medicine formulas ameliorate depression-like behaviors in rodents by remodeling gut microbiota and correcting perturbations in D-glutamate and D-glutamine metabolism.^351,425^ Furthermore, Mendelian randomization studies provide evidence for a causal chain where elevated BMI leads to lower circulating glutamine levels, which in turn contributes to the risk of developing MDD and specific depressive symptoms such as anhedonia.^426^ Supplementing with glutamine may help to increase glutamate levels and potentially alleviate depressive symptoms.^427^ However, more research is needed to confirm these findings and understand the optimal use of glutamine supplementation for depression.

#### Schizophrenia

In schizophrenia, the focus on dopaminergic dysfunction is now integrally complemented by the glutamatergic hypothesis, supported by both central and peripheral metabolic evidence. Preclinical models, such as ketamine-induced schizophrenia in rats, demonstrate a pathology characterized by decreased brain glutamine content and altered activities of key enzymes such as GS and GLS, indicating a profound disruption in glutamate recycling and homeostasis.^428^ Recent targeted metabolomics of peripheral blood in early-onset schizophrenia patients identified glutamine as one of several differentially expressed neurotransmitters, with its levels showing a positive correlation with the severity of positive symptoms and the total score on the Positive and Negative Syndrome Scale (PANSS).^429^ This suggests that peripheral glutaminergic signatures may reflect central pathological processes and could aid in diagnosis. The therapeutic potential of modulating this axis is highlighted by the neuroprotective effect of a plant-derived flavonoid against ketamine-induced glutamatergic dysregulation, which restored glutamine levels and enzyme activities comparably to the antipsychotic clozapine.^430^ Beyond the primary symptomatology, cognitive impairment is a core feature of schizophrenia. The involvement of neuronal nicotinic acetylcholine receptors (nAChRs), which are functionally interlinked with glutamatergic signaling, is recognized as crucial for cognitive function.^431^

Above evidences suggest that glutamine metabolism is a key link between energy homeostasis, neurotransmission, and neuroinflammation in the pathophysiology of neuropsychiatric disorders. Bidirectional communication with the gut microbiome adds another layer of complexity and therapeutic opportunity. Current strategies are evolving from generalized supplementation to precise interventions aimed at specific molecular targets, such as GS activation or GLS inhibition, or cellular populations, such as microglia.

## Clinical applications: nutritional support and critical care

### Evidence in surgical, trauma, and burn patients

The exploration of glutamine’s role transitions from the complex pathophysiology of neuropsychiatric disorders to the high-stakes arena of acute surgical and traumatic stress, where metabolic demands surge and systemic homeostasis is profoundly challenged. In these settings, glutamine has been extensively investigated as a therapeutic nutrient to modulate the hypermetabolic and catabolic response, support immune function, and ultimately improve clinical recovery. The evidence, however, presents a nuanced and sometimes contradictory picture, heavily influenced by patient population, timing, route of administration, and underlying pathology. In major gastrointestinal surgeries, particularly for cancer, perioperative glutamine supplementation has been associated with tangible benefits. A meta-analysis focusing on CRC patients after radical surgery concluded that glutamine significantly improved both humoral (IgA, IgM, IgG) and cellular (increased CD4+ and CD4+/CD8+ ratio) immune parameters.^432^ Clinically, this immunomodulation translated into a reduced risk of surgical site infection (RR = 0.48) and anastomotic leakage (RR = 0.23), alongside a significant shortening of hospital stay.^432^ These findings are supported by a large propensity score-matched study in CRC surgery, where postoperative parenteral glutamine supplementation was associated with a markedly lower incidence of overall complications (14.9% vs. 36.8%) and infectious complications (10.5% vs. 28.9%), as well as faster recovery of intestinal function and shorter hospitalization.^433^ The benefits may extend beyond infection control to mitigating sarcopenia, a key determinant of surgical outcome. In gastric adenocarcinoma patients undergoing gastrectomy, perioperative glutamine supplementation (parenteral plus oral) was significantly associated with the restoration of psoas muscle mass, countering the postoperative atrophy typically observed.^434^ Preoperative administration also appears impactful; in gastric cancer patients, preoperative glutamine (either oral or intravenous) significantly reduced the rate of serious surgical complications according to the Clavien‒Dindo classification, although it did not universally improve laboratory nutritional markers or platelet phagocytic activity.^435^

The narrative becomes more complex in the context of severe burns, a condition epitomizing hypermetabolism and glutamine depletion. Earlier small trials and mechanistic rationales suggested strong benefits, but large-scale, rigorous RCTs have tempered enthusiasm.^436^ The large-scale REDOX trial found no mortality benefit from glutamine supplementation and increased mortality in patients with multiorgan failure, raising safety concerns.^437^ The same term initiates another landmark RE-ENERGIZE trial, a double-blind, placebo-controlled study in 1200 patients with severe burns, which found that enteral glutamine supplementation did neither reduce the time to discharge alive from the hospital (median 40 vs. 38 days) nor affect 6-month mortality.^438^ A subsequent systematic review with trial sequential meta-analysis of ten RCTs in severely burned adults corroborated these findings, concluding that glutamine supplementation, regardless of route, does not improve mortality or infectious complications.^439^ This review noted a significant subgroup effect where single-center RCTs showed benefit but multicenter RCTs did not, and trial sequential analysis indicated that the former results were likely type 1 errors.^439^ The disconnect between promising preclinical models and null clinical trials is stark. For instance, mouse models of burn injury demonstrate that early enteral nutrition supplemented with glutamine effectively attenuates systemic and intestinal inflammatory responses, such as reducing serum TNF-α and IL-6.^440^ This underscores the challenge of translating controlled animal physiology to heterogeneous critically ill human populations, where factors such as timing, dose and concomitant therapies may dilute effect signals.

In polytrauma patients, another cohort with intense metabolic stress, glutamine’s role may be more supportive of metabolic homeostasis than directly altering hard clinical endpoints. A randomized study in critically ill polytrauma patients showed that parenteral glutamine dipeptide supplementation (0.5 g/kg/day) was associated with a lower incidence of hyperglycemic episodes and a reduced mean daily insulin requirement compared to standard nutrition, suggesting an improvement in glucose-insulin dynamics.^441^ This metabolic modulation could be particularly valuable in mitigating the IR characteristic of critical illness. Support for glutamine in specific, complex surgical scenarios comes from research on intestinal recovery and fistula management. In a rodent model of ileal pouch-anal anastomosis (a procedure performed in ulcerative colitis), enteral nutrition combined with glutamine improved nutritional status markers (total protein, albumin) more than enteral nutrition alone.^322^ Clinically, a retrospective comparative study with propensity score matching in patients with external duodenal fistula found that low-dose oral glutamine (10 g/day) promoted spontaneous fistula closure and was associated with a significantly shorter median hospital stay.^331^

The choice of nutritional support strategy itself can influence amino acid availability in unexpected ways. A substudy of an RCT in major rectal surgery patients found that despite early enteral nutrition leading to better clinical outcomes (reduced ileus, anastomotic leakage) than early parenteral nutrition, plasma levels of glutamine and arginine were actually lower in the enteral group.^442^ This challenges the simplistic assumption that higher plasma concentrations of conditionally EAAs directly equate to better clinical results, highlighting the complexity of whole-body metabolism and organ-specific utilization.

In orthopedic trauma and elective joint replacement, often involving older adults at risk of muscle loss, glutamine is frequently part of combined nutritional regimens. A systematic review on total hip and knee replacements noted limited but suggestive evidence that supplements containing amino acids such as glutamine, often combined with HMB and arginine, may help reduce muscle atrophy and accelerate functional recovery.^443^ A focused RCT in older adults with minor trauma tested an HMB complex (containing HMB, glutamine, and arginine) against a glutamine complex, finding no significant effect of the HMB formulation on muscle cross-sectional area or physical function outcomes at two weeks; however, elevated urinary titin, a biomarker of muscle injury, on day 3 correlated with poorer subsequent physical function, suggesting a role for such biomarkers in prognostication.^444^

Collectively, the evidence in surgical, trauma, and burn patients paints a picture of context-dependent utility. In elective or semielective major abdominal cancer surgery, the data robustly support perioperative glutamine supplementation to enhance immune competence, reduce infectious and noninfectious complications, and support muscle mass. In the chaotic metabolic storm of severe burns, large, methodologically sound trials have failed to demonstrate a mortality or major morbidity benefit, leading to a reevaluation of its routine use in this population. In polytrauma, benefits manifest in improved metabolic control. The ongoing challenge is to move beyond uniform supplementation strategies toward precision applications, identifying patient subphenotypes—based on the severity of depletion, genetic determinants of metabolism, or biomarker profiles such as heat shock protein 70 (HSP-70) or titin—that are most likely to derive meaningful clinical benefit from glutamine or its pharmacologic analogs.

### Use in critical illness and sepsis

An updated meta-analysis focusing specifically on enteral glutamine therapy in critically ill adults found no effect on hospital mortality, ICU length of stay, or infectious complications, although it did not show a reduction in overall hospital length of stay.^445^ The reasons behind these neutral or negative outcomes in broad ICU populations, contrasted with positive signals in conditions such as SAP, are a subject of intense investigation. One prominent hypothesis centers on the concept of a catabolic phenotype. A reanalysis of the large REDOXS trial, which found increased mortality with glutamine and antioxidant supplementation, identified the urea-to-creatinine ratio as a signature of persistent catabolism.^446^ This study suggested that the harmful effect of glutamine was mediated through this pathway, as glutamine administration provided an exogenous nitrogen load that exacerbated the catabolic state, leading to increased urea production. Mediation analysis indicated that the urea-to-creatinine ratio mediated the risk of death associated with glutamine randomization.^446^ This finding posits that in a subset of patients with severe, ongoing catabolism, glutamine supplementation may be metabolically detrimental rather than supportive.

Further complexity arises from patient subphenotypes. A post hoc analysis of the MetaPlus trial, which compared immune-modulating (with glutamine, fish oil, antioxidants) high-protein enteral nutrition to standard high-protein nutrition, found that the harmful effect on 6-month mortality was confined specifically to medical ICU patients.^235^ In this subgroup, the early increase in the plasma ratio of omega-3 fatty acids, rather than glutamine itself, was identified as a mediator of the negative treatment effect.^235^ This underscores that glutamine is rarely administered in isolation in clinical trials; it is typically one component of a multinutrient immunonutrition formula, making it challenging to disentangle its individual effects. Furthermore, the impact on specific organ systems or patient-centered outcomes beyond mortality is mixed. In critically ill children, glutamine supplementation contributed to maintaining elevated levels of HSP-70, a cytoprotective chaperone, longer than standard nutrition, although it did not significantly influence interleukin levels or duration of stay.^447^ Regarding the pervasive problem of ICU-acquired muscle wasting, a systematic review found that evidence for enteral amino acid supplements, including glutamine, to attenuate skeletal muscle loss is limited and highly heterogeneous, with no clear consistent benefit demonstrated.^448^

In summary, the role of glutamine in critical illness and sepsis epitomizes the principle that metabolic therapy must be precisely tailored to the patient’s underlying physiological state. While a strong pathophysiological rationale and positive meta-analytic evidence support its use in defined conditions such as severe acute pancreatitis and possibly in modulating inflammation in viral illnesses such as COVID-19, broad application in general ICU populations is not supported and may be harmful for certain subphenotypes, particularly medical patients with a pronounced catabolic signature. The dissociation between plasma glutamine levels and outcomes, the mediation of harm through catabolic pathways such as elevated urea-to-creatinine ratio, and the subpopulation-specific effects all argue against routine supplementation.^235,446,449^ The future of glutamine in critical care likely lies not in universal administration but in a precision medicine approach, potentially guided by dynamic metabolic biomarkers—such as glutamate levels, urea-to-creatinine ratio, or CRP thresholds—to identify patients who are truly deficient and capable of utilizing exogenous glutamine without exacerbating detrimental metabolic pathways.^318,446^ Until such biomarkers are validated and integrated into practice, current guidelines that caution against their routine use in critical illness are prudent, reserving consideration for specific clinical contexts where trial evidence is favorable.^450,451^

### Role in cancer therapy support

Building upon the nuanced role of glutamine in critical illness, its application in supportive cancer care presents a more defined therapeutic paradigm. Here, the goal is not to broadly counteract a systemic catabolic state but to specifically protect and nourish normal tissues under assault from cytotoxic therapies, mitigate treatment-limiting toxicities, and counteract cancer-associated wasting without interfering with the antitumor efficacy of chemotherapy or radiation.^452,453^ The rationale is grounded in the high demand of rapidly proliferating mucosal cells and immune cells for glutamine as a primary fuel and nitrogen donor, needs that are exacerbated during the metabolic stress of cancer treatment.^232,454^ Clinical research has therefore focused on three primary domains: the prevention and management of therapy-induced mucositis, the attenuation of cancer cachexia, and the reduction of other treatment-related complications, with a consistent emphasis on ensuring that supplementation does not promote tumor growth or impair survival outcomes.^452,455^

The most extensively studied application is the prevention and mitigation of mucosal injury, particularly oral mucositis and radiation-induced esophagitis, which are common dose-limiting toxicities of radiotherapy and certain chemotherapeutic regimens. Evidence supports that oral or topical glutamine can reduce the severity of these lesions. In patients with head and neck cancer undergoing chemoradiotherapy, supplementation with glutamine has been shown to delay the onset of oral mucositis and significantly reduce the incidence of severe (grade 3-4) mucositis.^455,456^ Similarly, in locally advanced non-small cell lung cancer patients treated with chemoradiotherapy, prophylactic oral glutamine significantly lowered the rate of severe acute radiation-induced esophagitis.^457^ The proposed mechanism involves glutamine serving as a crucial substrate for enterocyte and mucosal cell proliferation and repair, while its combination with disaccharides such as sucrose may enhance its topical uptake and protective effect on the mucosa.^454^ Importantly, these studies and others have consistently reported that glutamine supplementation does not adversely affect tumor control or survival. An RCT in head and neck cancer patients found no significant difference in 5-year overall survival or progression-free survival between the glutamine and placebo groups, confirming that amino acids do not compromise the antitumor effect of concurrent chemoradiotherapy.^455^ Glutamine supplementation has been identified as a potential dietary intervention to arrest melanoma tumor growth and sensitize tumors to targeted therapies through epigenetic reprogramming.^458^ These safety profiles are paramount for its adoption in clinical support protocols.

Beyond direct mucosal protection, glutamine is a key component of immunonutrition formulas, which are designed to modulate the immune and inflammatory response in the perioperative or peritherapeutic period for cancer patients. Systematic reviews and meta-analyses have demonstrated that perioperative immunonutrition containing glutamine, often combined with arginine and omega-3 fatty acids, significantly reduces postoperative infectious and total complication rates in patients undergoing surgery for gastrointestinal or head and neck cancers.^459^ This effect is attributed to the modulation of specific immune and inflammatory pathways, counteracting the immunosuppression and cytokine dysregulation commonly seen in malnourished cancer patients.^231,232^ For instance, a network meta-analysis in colorectal cancer patients indicated that glutamine supplementation was particularly effective in decreasing proinflammatory tumor necrosis factor-α levels and ranked highest in reducing hospital length of stay and wound infection incidence.^460^ In gastric cancer surgery patients, perioperative parenteral glutamine supplementation showed a positive, dose-dependent effect on the recovery of serum albumin levels, a marker of nutritional status and inflammatory control, although it did not directly alter surgical outcomes in that study.^461^ These findings underscore glutamine’s role in ameliorating the surgical stress response and improving postoperative recovery within enhanced recovery after surgery (ERAS) protocols, including in complex scenarios such as pancreatic cancer surgery.^459^

Cancer cachexia, a multifactorial syndrome characterized by progressive weight loss and muscle wasting, remains a major challenge that impairs treatment tolerance and survival. While glutamine monotherapy is not a cure for cachexia, its metabolic role intersects with this condition. Altered plasma amino acid profiles, including disturbances in glutamine metabolism, are evident in cachectic cancer patients.^462^ Glutamine supplementation aims to support protein synthesis and mitigate muscle catabolism. Clinical observations indicate that glutamine supplementation can help maintain body weight during treatment, as seen in lung cancer patients where the glutamine-free group experienced significantly higher weight loss, a factor that nearly reached significance as a poor prognosticator for overall survival.^457^ Metabolic dysregulation in cachexia may also inform risk prediction; although not specific to glutamine, metabolomic analyses reveal that perturbations in energy metabolism pathways, potentially linked to the mitochondrial use of substrates, can predict continued weight loss in cachectic patients.^462^ This suggests a complex interplay where glutamine metabolism is part of a broader altered metabolic landscape in cachexia.

The utility of glutamine extends to managing specific chemotherapy toxicities. Preclinical models indicate a protective role against chemotherapy-induced genomic damage.^463^ In the clinical management of hematological malignancies, the profound depletion of plasma glutamine is a known consequence of L-asparaginase therapy, an enzyme used to treat ALL that hydrolyzes both asparagine and glutamine.^464^ This iatrogenic glutaminolysis underscores the metabolic vulnerability induced by some therapies and highlights the need for careful metabolic monitoring, even if glutamine supplementation in this context is not standard practice due to theoretical concerns of blunting therapeutic efficacy.^452,464^

Finally, the intersection of glutamine biology with cancer therapy is an inspiring novel therapeutic strategy beyond nutritional support. The addiction of many tumors to glutamine and the overexpression of glutamine transporters such as SLC1A5 have been exploited for targeted drug delivery. Conjugating chemotherapeutic agents such as doxorubicin to glutamine-modified carriers such as β-cyclodextrin facilitates selective uptake into TNBC cells via SLC1A5, enhancing tumor-specific cytotoxicity while minimizing off-target effects on normal tissues.^465^ Furthermore, natural compounds such as epigallocatechin gallate are being investigated for their ability to suppress tumor-promoting metabolic reprogramming, including glutamine metabolism, thereby potentially sensitizing cancers to treatment. These approaches reflect a paradigm where understanding glutamine metabolism informs not only supportive care but also the development of direct anticancer strategies that exploit this metabolic vulnerability.

## Conclusion and future perspective

The preceding exploration of glutamine’s multifaceted roles underscores its position as a metabolic nexus that profoundly influences human physiology and the pathogenesis of a diverse spectrum of diseases. As the most abundant circulating amino acid, glutamine’s functions extend far beyond its classical role as a protein building block, encompassing critical contributions to bioenergetics, biosynthesis, redox homeostasis, and cellular signaling.^4,5,16^ Its metabolism is dynamically regulated and exhibits remarkable tissue-specific and context-dependent plasticity, a feature that underpins its involvement in health but also its exploitation in pathological states. The burgeoning field of glutamine biology, fueled by advances in metabolomics, molecular profiling and metabolic imaging, has transitioned from descriptive observation to a mechanistic understanding that reveals glutamine metabolism not merely as a passive fuel source but as an active driver and a vulnerable dependency in numerous conditions, from cancer and autoimmunity to fibrosis and neurodegeneration. The central challenge and opportunity now lie in translating this profound basic science knowledge into effective clinical strategies that can modulate glutamine metabolism for therapeutic benefit, a task that requires navigating its inherent complexity, heterogeneity and double-edged effects within integrated biological systems.

Reflecting on the key themes elucidated in this review, the reprogramming of glutamine metabolism stands as a hallmark of cancer, supporting rapid proliferation, survival under stress, and interaction with the TME.^78^ However, this dependency is not universal but highly heterogeneous, influenced by tissue of origin, oncogenic drivers, and nutrient availability.^466^ This heterogeneity is a primary reason why monotherapies targeting glutamine metabolism, such as GLS inhibitors, have faced significant challenges in the clinic, often eliciting adaptive resistance mechanisms that allow cancer cells to circumvent the blockade.^44,181,467^ Similarly, in immune-mediated diseases, glutamine serves as a critical modulator of T cell fate, influencing the balance between proinflammatory Th17 cells and Tregs, with clear implications for autoimmune disorders, psoriasis, and neuroinflammation.^215,222,468^ Beyond oncology and immunology, dysregulated glutamine metabolism contributes to the pathogenesis of cardiometabolic diseases, influencing insulin signaling, vascular function, and fibrotic processes in the liver, lungs, and kidneys.^14,307^ In the nervous system, the glutamate-glutamine cycle is fundamental for neurotransmission and neuronal health, with disruptions implicated in neurodegenerative and psychiatric disorders.^354^ This pervasive involvement across organ systems and disease states highlights glutamine as a central metabolic node but also forewarns of the potential for on-target, off-tissue toxicities when attempting systemic pharmacological inhibition.

The current clinical landscape regarding glutamine modulation is dichotomous, reflecting its dual nature as both a nutrient and a pathological driver. On one hand, glutamine supplementation has established itself in supportive care protocols. Evidence supports its use in reducing the severity and duration of chemotherapy- and radiotherapy-induced mucositis, improving outcomes in surgical and critically ill patients by modulating inflammation and supporting gut barrier function, and as a component of immunonutrition formulas in gastrointestinal malignancies.^231,454,460^ On the other hand, the therapeutic inhibition of glutamine metabolism, particularly in oncology, remains an area of intense investigation but with more limited clinical success to date. While numerous preclinical studies have validated enzymes like GLS and transporters as targets, and compounds like CB-839 have advanced to clinical trials, the results have underscored the complexities of targeting a metabolic pathway that is also crucial for immune cell function.^21,83,469^ This has led to a paradigm shift from standalone inhibition to rational combination strategies. These include pairing glutamine metabolism inhibitors with other targeted therapies, chemotherapy, immunotherapy, or other metabolic interventions to overcome adaptive resistance and leverage synthetic lethality.^181^

Looking ahead, advancements in basic science and its translation into clinical practice will depend on progress across several key frontiers. First, the embrace of metabolic heterogeneity demands the development of robust predictive biomarkers to enable patient stratification. Identifying individuals who could benefit from targeted therapy or dietary supplementation is essential. Advanced diagnostic tools will be critical here. Metabolic imaging using tracers such as 18F-FGln and liquid biopsy approaches offers a noninvasive method to visualize and quantify glutamine flux in tumors in vivo, potentially guiding patient selection and monitoring therapeutic response.^470–473^

Second, therapeutic innovation must extend beyond simple enzyme inhibition to address the dynamic adaptability of metabolic networks. The emerging concept of a hormetic response to glutamine stress underscores the need for strategies that preempt or cotarget these resistance mechanisms.^467^ Nanomedicine and prodrug strategies aimed at improving the bioavailability, tumor specificity, and delivery efficiency of metabolic inhibitors or combining them with agents that alter the nutrient composition of the TME, such as ketogenic metabolic therapy, represent exciting technological avenues to enhance efficacy and reduce toxicity.^474,475^

Finally, a truly integrated approach requires a holistic view that considers the host as a whole. The systemic effects of glutamine metabolism modulation—on systemic immunity, organ function, and even the gut microbiome—must be carefully evaluated.^476,477^ Nutritional status and dietary intake can significantly influence the baseline metabolism and therapeutic window for glutamine-targeting agents. Therefore, future clinical trials should incorporate detailed metabolic phenotyping, dietary assessment, and monitoring of immune parameters. By integrating insights from cancer biology, immunology, neuroscience and clinical nutrition—and harnessing advanced tools in imaging, omics and data science—knowledge graphs and artificial intelligence are positioned to unlock the full therapeutic potential of targeting the glutamine nexus. Although the journey from foundational discovery to clinical application will be complex, it offers the prospect of novel and effective strategies for addressing some of the most challenging human diseases.
